# From 2D to 3D Colorectal Cancer Patient-Derived Organoids (PDOs) and *In Vivo* Models: Copper(II) Complexes as Promising New Antitumor Agents

**DOI:** 10.1021/acs.jmedchem.6c00941

**Published:** 2026-07-31

**Authors:** Katarzyna Choroba, Sandra Cordeiro, Rita Sequeira, Barbara Machura, Karol Erfurt, Ana Guedes, Luís Mascarenhas-Lemos, Marília Cravo, Pedro V. Baptista, Alexandra R. Fernandes

**Affiliations:** † Institute of Chemistry, University of Silesia, Szkolna 9, 40-006 Katowice, Poland; ‡ Associate Laboratory i4HB - Institute for Health and Bioeconomy, NOVA School of Science and Technology, NOVA University Lisbon, 2819-516 Caparica, Portugal; § UCIBIO, Departamento de Ciências da Vida, NOVA School of Science and Technology, Campus de Caparica, 2829-516 Caparica, Portugal; ∥ Department of Chemical Organic Technology and Petrochemistry, 49569 Silesian University of Technology, Krzywoustego 4, 44-100 Gliwice, Poland; ⊥ Hospital Beatriz Ângelo, Avenida Carlos Teixeira 3, 2674-514 Loures, Portugal; # Hospital da Luz Learning Health, Luz Saúde, Avenida Lusíada 100, 1500-650 Lisboa, Portugal; ∇ 199396 Hospital da Luz, Avenida Lusíada 100, 1500-650 Lisboa, Portugal; ○ NOVA Medical School, Campo Mártires da Pátria 130, 1169-056 Lisboa, Portugal; ◆ Catolica Medical School, Palma de Cima, 1649-023 Lisboa, Portugal; ¶ Lisbon School of Medicine, Universidade de Lisboa, Avenida Professor Egas Moniz, 1649-028 Lisboa, Portugal

## Abstract

Two copper­(II) complexes, **(1)** [CuCl_2_(tmp-terpy)] and **(2)** {[CuCl­(tmp-terpy)]·PF_6_}_n_, (tmp-terpy-4′-(3,4,5-trimethoxy-phenyl)-2,2′:6′,2″-terpyridine) were designed, characterized, and evaluated for cytotoxicity in 2D human cell models (HCT116, HCT116-DoxR, and in normal primary fibroblasts), and 3D colorectal cancer (CRC) spheroids and patient-derived organoids (PDOs). Both complexes demonstrated potent antiproliferative effects, alone and in combination with the FOLFOX (FF), across all 3D models. Importantly, the cytotoxic effects of complex **1** were significantly more pronounced in tumor-derived PDOs than in normal tissue-derived PDOs. Complex **1** induced reactive oxygen species (ROS) production, triggering intrinsic apoptosis and autophagy, consistent with a multimodal anticancer mechanism. It also exerted a cytostatic effect, as shown by delayed cell-cycle progression, and antiangiogenic activity *in vivo* without systemic toxicity. Notably, cotreatment of complex **1** with FF significantly potentiated cytotoxicity in 3D tumor spheroids and PDOs tumor-derived-PDOs. [CuCl_2_(tmp-terpy)] alone or in combination with the FF regimen warrants further evaluation in advanced preclinical models.

## Graphical abstract

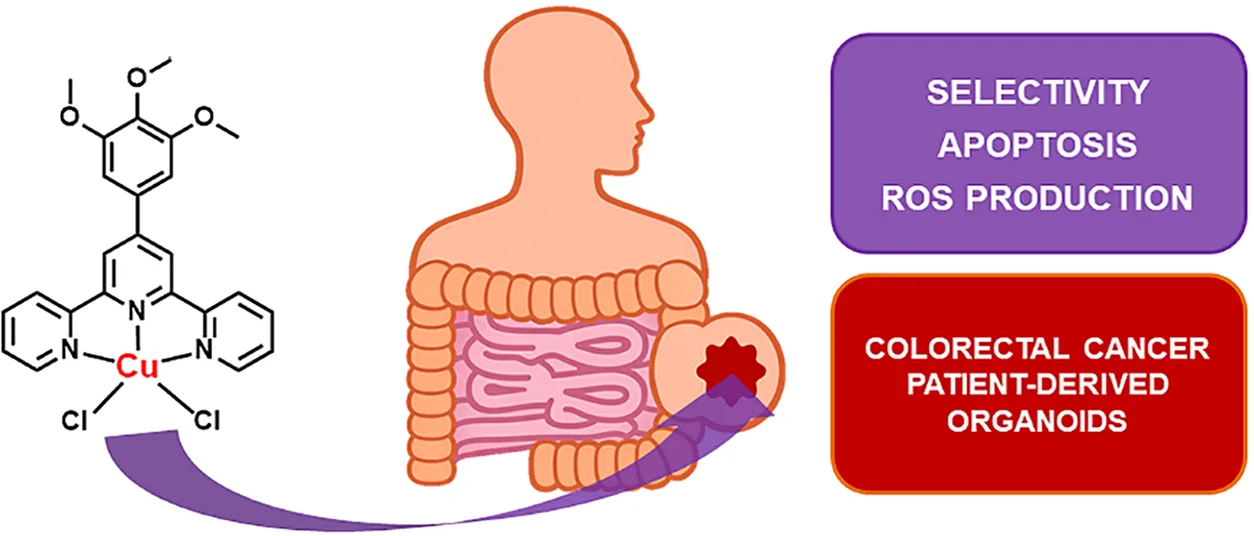

## Introduction

Colorectal cancer (CRC) is among the deadliest and most widespread cancer types worldwide, with nearly 2 million new cases and around 0.9 million deaths every year.^1,2^ When diagnosed in earlier stages, the primary treatment is surgery. However, when CRC is detected in advanced stages (III and IV), it is necessary to resort to chemotherapy. CRC’s chemotherapy includes both monotherapy and a combination of regimens, with 5-fluorouracil (5-FU) as the backbone of most treatments. Common clinical combinations include FOLFOX (FF; folinic acid (FA), 5-FU, and oxaliplatin (OX)), FOLFIRI (FA, 5-FU, and irinotecan), CAPOX (capecitabine and OX), and CAPIRI (capecitabine and irinotecan). These regimens are limited by systemic toxicity, modest response rates, poor tumor selectivity, and the development of acquired resistance. Consequently, there remains a strong need for novel agents with improved antiproliferative efficacy and superior therapeutic indexes.^3–9^

Our recent work revealed that 2,2′:6′,2″-terpyridine (terpy) derivatives and their transition metal complexes show strong potential against CRC.^10–13^ Owing to terpy’s excellent metal-chelating properties and the structural versatility enabled by efficient condensation reactions with 2-acetylpyridine and aldehydes, this platform offers significant opportunities for downstream development and preclinical evaluation.^14,15^ Overall, free ligands show markedly lower antiproliferative activity than their corresponding metal complexes, underscoring the crucial role of the metal center in biological interactions.^12,13^

Studies have shown that Au­(II) and Cu­(II) complexes of terpy ligands bearing 4-methoxyphenyl, 4-methoxynaphthyl, or 2-quinolinyl substituents exhibit higher cytotoxicity than both Pt­(II) terpy complexes and cisplatin (Cis). Moreover, for 2-quinolinyl-substituted terpy complexes, cytotoxicity follows the order Cu­(II) > Au­(II) > Pt­(II) and correlates with increased planarity of the complex, which enhances DNA double-helix destabilization.^12^ It was demonstrated that the complexes [Cu_2_Cl_2_(R-terpy)_2_]­(PF_6_)_2_ bearing 4-quinolinyl, 4-methoxy-1-naphthyl, 2-furanyl, or 2-pyridyl as R substituents cause cancer apoptosis and autophagy through elevated ROS levels, halt in cell-cycle progression, and senescence.^16–20^ These complexes also exhibit antimetastatic and antiangiogenic activities, cleave plasmid DNA, and interact with bovine serum albumin, while showing no *in vivo* toxicity.^11^

Three-dimensional (3D) spheroids more closely resemble *in vivo* tumors compared to cells growing in a two-dimensional (2D) monolayer.^2,21,22^ In contrast to monolayer cultures, in which cells are uniformly exposed to nutrients, oxygen, and therapeutic agents, the architectural complexity of the tumor microenvironment (TME), together with increasing tumor size, imposes critical diffusion limitations. These constraints particularly affect oxygen and nutrient transport, thereby promoting intratumoral heterogeneity, while simultaneously hindering effective drug penetration. This phenomenon has been linked to the development of multidrug resistance (MDR).^22,23^ Both intratumoral heterogeneity and MDR are strongly associated with loss of therapeutic response in CRC patients.^24^ 3D tumor spheroids, derived from drug-resistant cells, are crucial for the development of novel chemotherapy regimens that might circumvent MDR mechanisms.^25–27^ In recent years, substantial attempts have been directed toward reducing the reliance on *in vivo* murine preclinical studies, owing to ethical concerns and their limited ability to faithfully recapitulate the human disease context.^28^ Patient tissue–derived organoids (PDOs), generated from both normal and tumor tissues, provide a more physiologically relevant platform for drug screening, enabling the investigation of both inter- and intratumor heterogeneity, as well as the assessment of potential side effects using PDOs derived from normal tissue.^2,29^ Despite significant advances in the development of terpyridine-based metallodrugs for CRC therapy, their anticancer activity has not yet been evaluated in complex 3D models such as PDOs. Therefore, systematic and comprehensive studies are required to support the rational design of more effective CRC chemotherapeutics capable of overcoming MDR while exhibiting improved selectivity indices.

Improving our previous studies on the antiproliferative activity of transition metal complexes with 2,2′:6′,2″-terpy derivatives,^10–13,30–34^ we have developed two novel Cu­(II) coordination complexes with 4′-(3,4,5-trimethoxy-phenyl)-2,2′:6′,2″-terpyridine (tmp-terpy) (Scheme 1) and validated their potential using more complex *in vitro* models—3D tumor spheroids and PDOs, as well as, *in vivo* models. As we demonstrated in our latest work,^30^ the tmp-terpy ligand significantly enhanced the anticancer potential of terpyridyl Au­(III) and Pt­(II) complexes in CRC chemotherapy (Scheme S1). Considering the pivotal biological roles of Cu­(I) and Cu­(II) ions in enzymatic catalysis, electron-transfer processes, and the regulation of cellular redox homeostasis,^35–37^ we hypothesized that coordination of the tmp-terpy ligand to Cu­(II) centers could yield even more promising metal-based agents capable of effectively interacting with human colorectal carcinoma cell lines. The therapeutic potential of [CuCl_2_(tmp-terpy)] (**1**) and {[CuCl­(tmp-terpy)]·PF_6_}_n_ (**2**) against colorectal cancer 2D and 3D models, including patient-derived organoids, to *in vivo* models. To further elucidate the biological activity of the most promising Cu­(II) complex, a comprehensive set of *in vitro* and *in vivo* studies was conducted. Those included the evaluation of the induced cell death mechanisms, the ability to trigger reactive oxygen species (ROS) production, modifications in mitochondrial membrane potential, and the assessment of cytostatic activity and angiogenesis-modulatory effects. Overall, through this study, we have identified and thoroughly characterized one of the most effective terpyridyl copper-based metallodrugs for CRC therapy, with impressive cytotoxic activity against the CRC doxorubicin (Dox)-resistant (R) cell line, HCT116-DoxR. As far as we are aware, this is the first report on the use of CRC PDOs to validate the antitumor potential of Cu­(II) coordination complexes alone or in combination with CRC patients’ chemotherapeutic regimens.

**Scheme 1:**
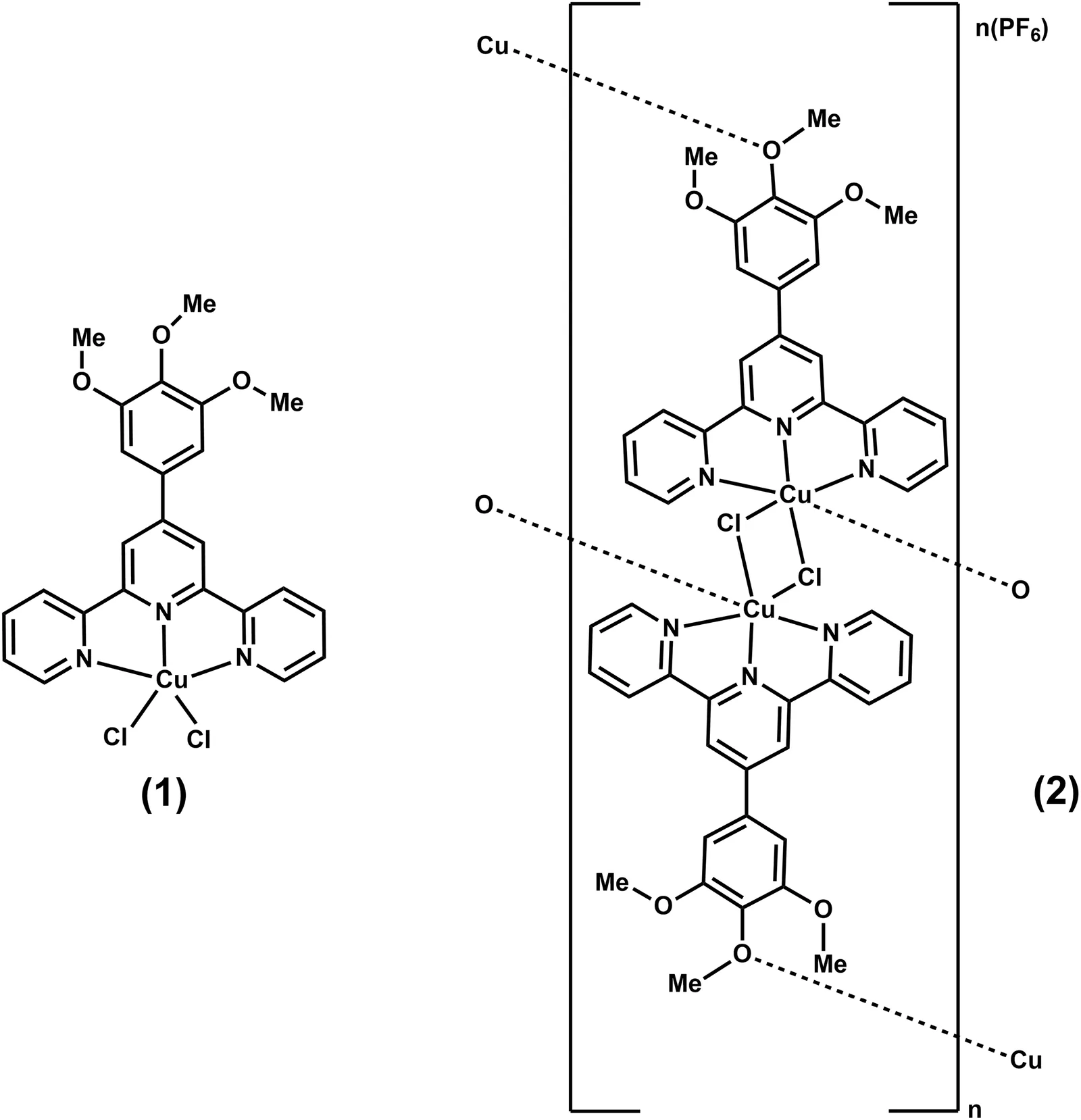
Copper­(II) Compounds Reported in This Study (**1** and **2**)

## Results and Discussion

### Preparation, Molecular Structure, and Solution Properties

The complexes [CuCl_2_(tmp-terpy)] (**1**) and {[CuCl­(tmp-terpy)]·PF_6_}_n_ (**2**) were prepared by the reaction of CuCl_2_ and 4′-(3,4,5-trimethoxy-phenyl)-2,2′:6′,2″-terpyridine (tmp-terpy) in 1:1 molar ratio, carried out in the methanol: chloroform, mixture (1:1) at room temperature for **1** and water:methanol mixture (1:1) in the presence of NH_4_PF_6_ at reflux in the case of **2**. For both compounds, X-ray quality monocrystals were obtained by the slow evaporation from acetonitrile:methanol solution at ambient conditions, and their crystal structures were determined using the X-ray diffraction technique (Tables S1–S6, Figure S1).

The molecular structure and crystal packing arrangement of **1** are shown in Figure 1. Five-coordinated molecules of [CuCl_2_(tmp-terpy)] (Figure 1A) show a strongly distorted square-based pyramidal geometry, as supported by the angular structural index τ_5_ ^38–40^ and SHAPE measure *S*_Q_(P) parameters^41,42^ (Table S7). The terpy-based ligand coordinates to the metal center in a tridentate mode (terpy-κ^3^N), and two chloride ions complement the coordination sphere of Cu­(II). An elongation of the Cu–Cl apical bond (2.3752(8) Å) relative to the Cu–Cl basal one (2.2960(8) Å) is rationalized by Jahn–Teller distortion, typical of square-pyramidal Cu­(II) complexes.^43^ In agreement with the tridentate terpy coordination mode,^44^ the Cu–N_central pyridine_ bond length (1.959(2) Å) is markedly shorter relative to those between the Cu­(II) ion and terminal pyridines (2.042(2) and 2.019(2) Å), and the angles N–Re–N are noticeably smaller than 90° (78.56(8) and 78.88(9)°). Ph-terpy moiety remains almost coplanar, with the twist angle between the least-squares of the phenyl and terpy planes of 4.80°, and dihedral angles between the central and distal pyridines of 2.77 and 4.29° (Table S8) The planar Ph-terpy domains of **1** are arranged in the layers (Figure 1B), stabilized by short intermolecular hydrogen bonds, π···π and C—H···π interactions (Tables S4 and S6). Typical of square-pyramidal Cu­(II) complexes,^10,11,33,45–47^ HR-MS spectrum of **1** with electrospray ionization shows the *m*/*z* signal corresponding to the [CuCl­(tmp-terpy)]^+^ ion (Figure S2).

**Figure 1:**
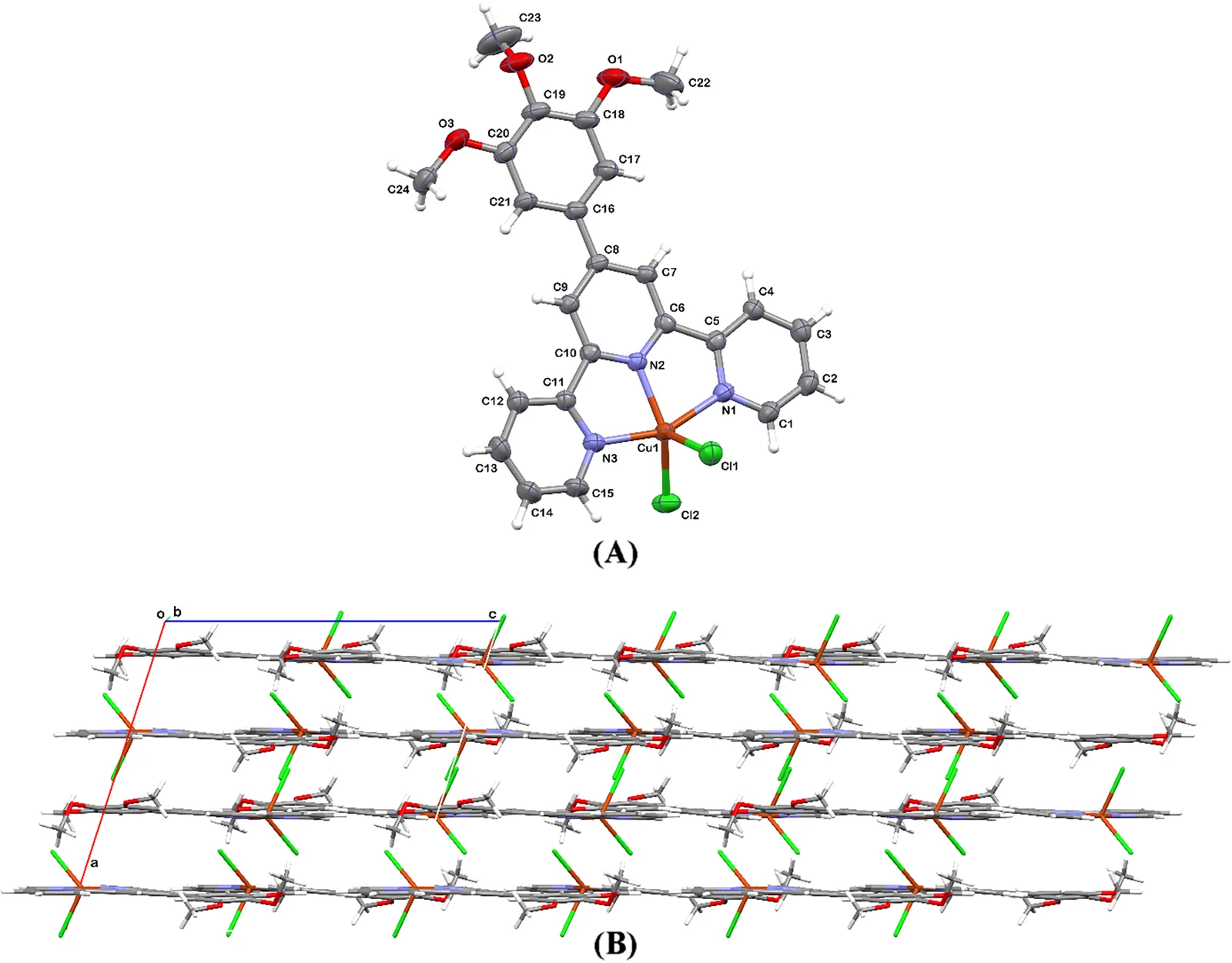
Molecular structure of **1** with thermal ellipsoids of non-hydrogen atoms given at 50% probability level (A); crystal packing arrangement of **1** along the *b* axis (B). CCDC number: 2494267.

In the crystal lattice of **2**, the mononuclear units [CuCl­(tmp-terpy)]^+^ are doubly bridged by two chloride ions to form dimeric motifs [Cu_2_Cl_2_(tmp-terpy)_2_]^2+^, counterbalanced by PF_6_^–^ ions. The coplanar Cu­(μ-Cl)_2_Cu core shows a rhomboidal geometry, with the Cu–Cl–Cu bridging angle of 88.68(3)°, Cu–Cl bond lengths of 2.2205(8) and 2.8563(11) Å, and intradimer separations of copper-to-copper and chloride-to-chloride of 3.577 and 3.658 Å, respectively. The coordination geometry around the Cu­(II) ions in [Cu_2_Cl_2_(tmp-terpy)_2_]^2+^ is further complemented by the oxygen atom from the *para*-methoxy group of the neighboring dimeric motifs [Cu_2_Cl_2_(tmp-terpy)_2_]^2+^. The Cu–O distance (2.656(2) Å) is indicative of a weak interaction between the central metal ion and the methoxy oxygen atom. Consequently, complex **2** displays a two-dimensional coordination network in a solid state, and the geometry of each copper­(II) center is best described as a distorted octahedron (Figure 2). The Cu–N bond lengths show a similar trend as in **1**, manifesting a noticeable elongation of Cu–N_distal pyridine_ bond lengths (2.030(3) and 2.017(3) Å) relative to Cu–N_central pyridine_ one (1.943(2) Å). HR-MS spectroscopy (Figure S2) with electrospray ionization of **2** in the positive ion mode did not confirm the dimer peak [Cu_2_Cl_2_(tmp-terpy)_2_]^2+^. By analogy to complex **1**, only the *m*/*z* signal corresponding to the isotopic distributions of [CuCl­(tmp-terpy)]^+^ ion is observed. The HR-MS spectrum in negative ion mode comprises only the *m*/*z* signals corresponding to the isotopic distributions of PF_6_^–^. The 1:1 electrolyte nature of ([CuCl­(tmp-terpy)]^+^PF_6_^–^) in solution, rather than 2:1 stoichiometry expected for [Cu_2_Cl_2_(R-terpy)_2_]^2+^(PF_6_^–^)_2_, was confirmed by conductivity measurements (Table S9). The H_2_O solution of complex **2** exhibited a molar conductivity of 107.9 S·cm^2^·mol^–1^, while in MeCN 125.9 S·cm^2^·mol^–1^, both values consistent with the range typical for 1:1 electrolytes.^48–50^

**Figure 2:**
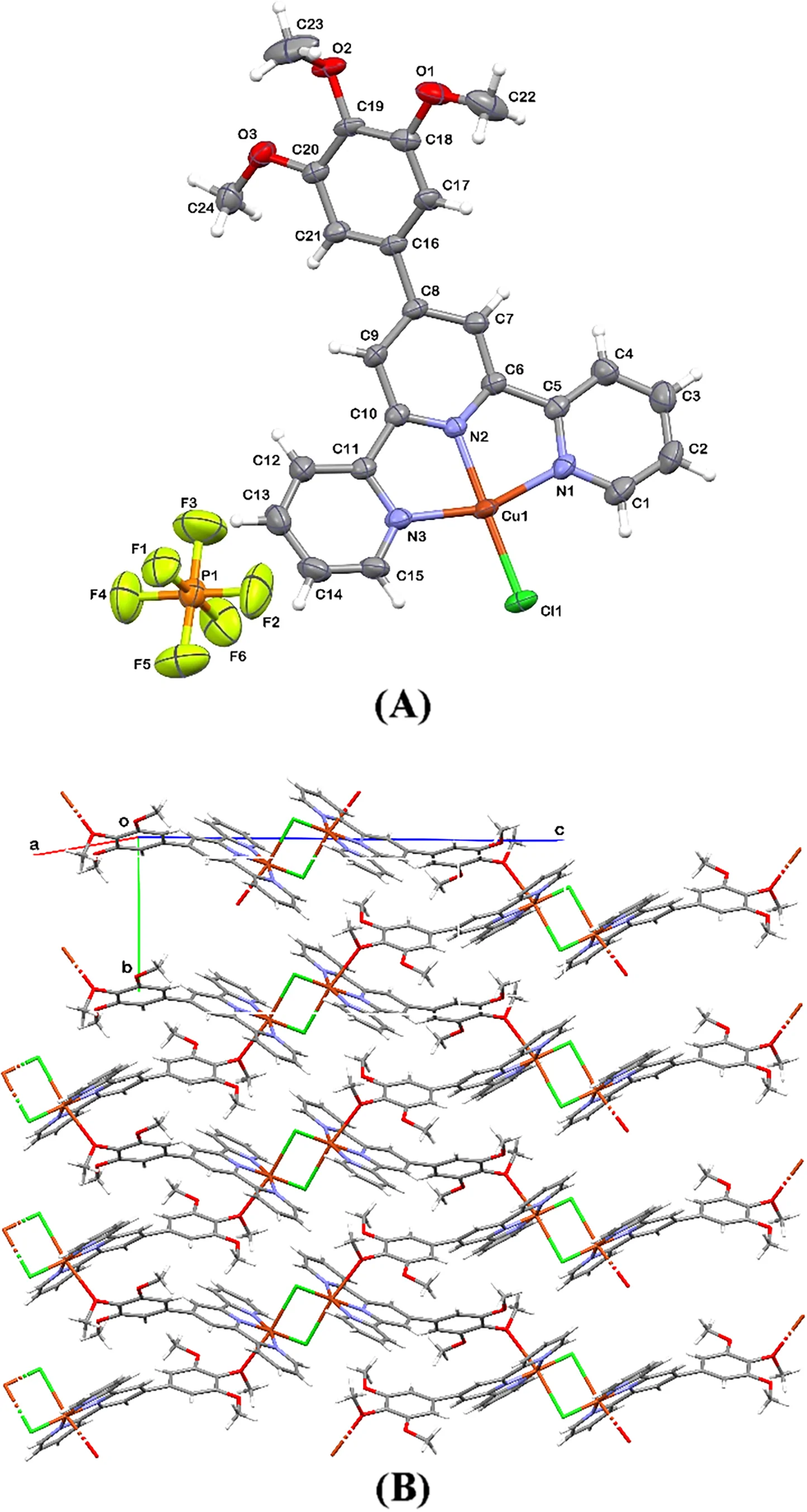
Asymmetric unit of structure **2**, with thermal ellipsoids of non-hydrogen atoms given at 50% probability level (A); two-dimensional coordination network of **2** (B). CCDC number: 2494268.

The successful coordination of the tmp-terpy ligand to Cu­(II) ion and formation of **1** and **2** complexes was also supported by elemental analysis and FT-IR spectroscopy (Figure S3). Upon coordination of tmp-terpy to the Cu­(II) ion, its characteristic ν_C=N_ and ν_C=C_ stretching modes are shifted to higher wavenumbers relative to the free ligands. In addition, the high purity of both compounds was evidenced by ultraperformance liquid chromatography (UPLC) with photodiode array UV–Vis spectroscopy (PDA UV–Vis) detection (Figure S4). The electronic absorption spectra of **1** and **2** recorded using the UPLC technique are in excellent agreement with those obtained for their acetonitrile solutions by UV–Vis spectroscopy (Figure S5). The absorptions in 200–400 range of **1** and **2** are dominated by π→π* and intraligand charge transfer (ILCT) transitions of the terpy-based ligand. The latter ones (ILCT) result due to the charge delocalization from the electron-rich 3,4,5-trimethoxy groups to the terpy acceptor moiety, and undergo a noticeable bathochromic shift in relation to the free ligand upon coordination to the Cu­(II) ion. A very weak and broad band of originating from overlapping d_*xy*_ → d_*x* ^2^–*y* ^2^_, d_*yz*_, d_*xz*_ → d_*x* ^2^–*y* ^2^_, and d_*z*_^2^ → d_*x* ^2^–*y* ^2^_ transitions occurs in the range 500–1000 nm, and it can be recoded in a more concentrated DMSO solution (1 mM, Figure S6).

### Lipophilicity and Stability Assays

The lipophilicity of **1** and **2**, expressed by partition coefficients logP, was evaluated using the classical shake-flash procedure in the two-phase system of immiscible solvents *n*-octanol–PBS buffer at room temperature.^51,52^ The partitioning of **1** and **2** between the aqueous environment and organic phase was monitored by UV–Vis spectroscopy. Both compounds are highly hydrophilic, with logP < 0. The higher lipophilic character was evidenced for **2**, with logP value of −0.88 ± 0.16. The logP value for compound **1** was −1.11 ± 0.13.

The stability of **1** and **2** was examined by recording the electronic spectra every 4 h over 48 h in MeCN, phosphate-buffered saline (PBS), and DMSO (Figure S7), which was used to prepare the stock solution for biological experiments. In all solvents, no changes in the spectral profiles of **1** and **2** were observed, confirming their stability in solution and biological media and thus justifying their use in biological studies.

### Biological Activity

#### Cell Viability Evaluation in 2D Models

The cytotoxic activity of complexes **1** and **2** in tumor (CRC cells, HCT116, and Dox-resistant CRC cells, HCT116-DoxR) and in nontumor (normal human primary dermal fibroblasts) cell lines was evaluated via the MTS assay, a colorimetric assay used to indirectly quantify the percentage (%) of viable cells, 48 h after the exposure of cells to increasing concentrations of complexes **1**, **2** (0.1–50 μM). As shown in Figure 3, cell viability declines in a dose-dependent manner. Concentration–viability data were used to calculate the relative IC_50_ values for each complex in each cell line, defined as the concentration that results in a 50% reduction in cell viability (Table 1). Moreover, cell viability was also compared for the FF therapeutic regimen (concentrations between 0.06 and 200 μM; FF regimen was prepared with the ratios 1 5-FU: 0.042 FA: 0.012 OX, and the concentrations are expressed based on 5-FU concentration) (Figure S8), Dox, and Cis (Figure S9).

**Figure 3:**
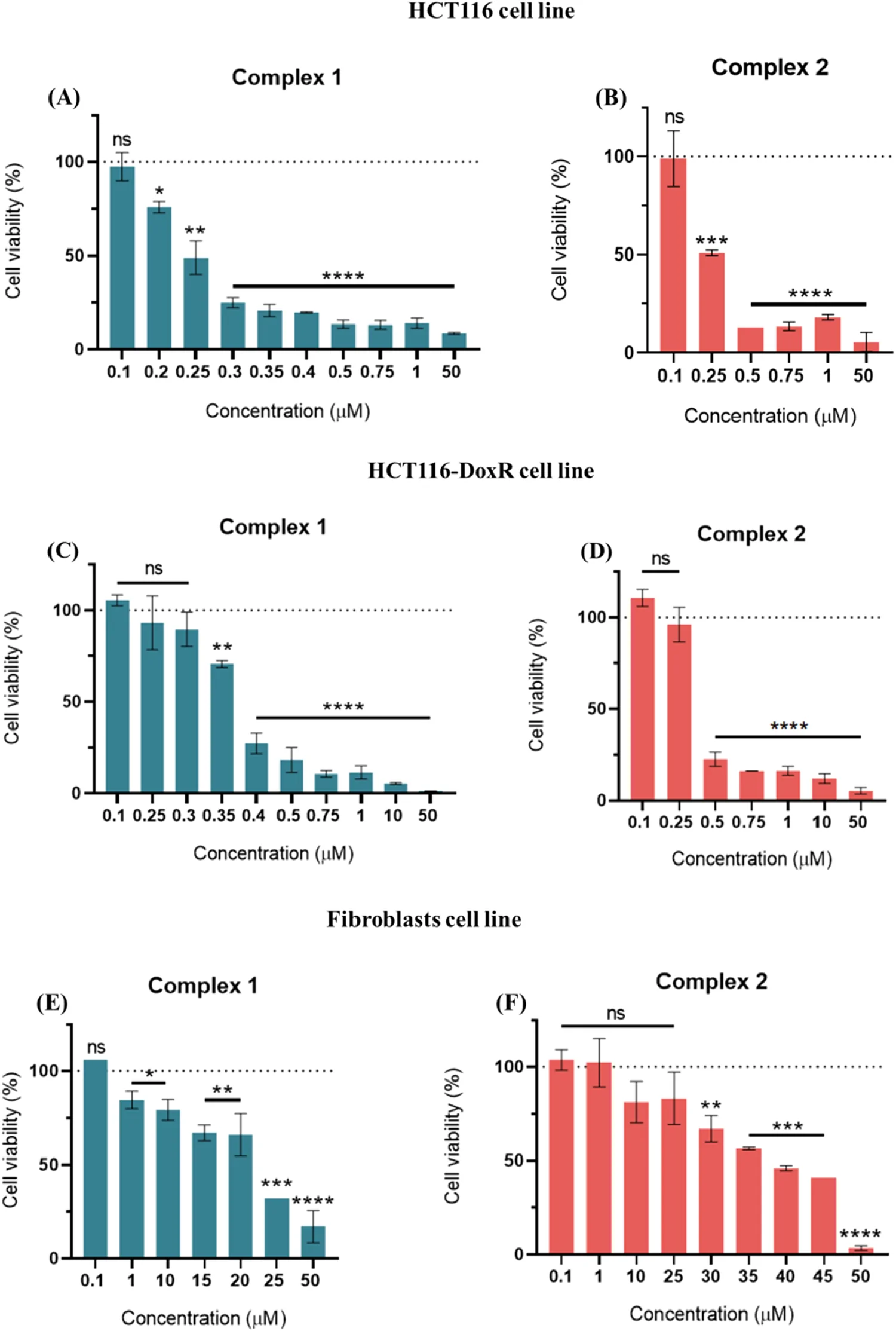
HCT116 (A and B), HCT116-DoxR (C and D) and fibroblasts (E and F) viability after exposure to several concentrations of copper complexes **1** and **2** for 48 h. The vehicle control, at the same final concentration used for the complexes, is represented as a dashed line. Data are presented as mean ± SEM from a minimum of three independent biological replicates. Statistical significance relative to the DMSO control was determined using one-way ANOVA (ns, not significant; *p < 0.05; **p < 0.01; ***p < 0.001; ****p < 0.0001).

**Table 1:** Relative IC_50_ Values Obtained for Each Complex and FF Chemotherapeutic Regimen in 2D Cultures of HCT116, HCT116-DoxR, and Fibroblasts^a^

complex	cell line	relative IC_50_ (μM)	SI
**1**	HCT116	0.24 ± 0.01	138.3
	HCT116-DoxR	0.35 ± 0.01	94.8
	fibroblasts	33.18 ± 0.1	
**2**	HCT116	0.23 ± 0.04	174.4
	HCT116-DoxR	0.33 ± 0.03	121.5
	fibroblasts	40.11 ± 0.14	
FF	HCT116	2.79 ± 0.08	>71.7
	HCT116-DoxR	1.35 ± 0.06	>148.1
	fibroblasts	>200	
cisplatin (Cis)	HCT116	15.60 ± 5.30	0.6
	HCT116-DoxR		
	fibroblasts	8.80 ± 2.90	
Dox	HCT116	0.50 ± 0.10	24.2
	HCT116-DoxR	>6 ^53^	>2
	fibroblasts	12.10 ± 0.20	

a The respective selectivity index (SI) after 48 h of exposure is also depicted. Values are expressed as the mean ± SEM from a minimum of three independent biological replicates. The reported values for FF reflect the IC_50_ of 5-FU, since it is the major component of FF. For the lasting FF components, their concentration was calculated based in the proportions: 1 (5-FU): 0.042 (FA): 0.012 (OX).

Complexes **1** and **2** exhibited notable cytotoxic activity, with low IC_50_ values (between 0.23 and 0.35 μM) in 2D cultures of CRC sensitive and resistant cell lines (Table 1). Although not statistically significant, the IC_50_ values of complex **1** and complex **2** are slightly higher for the HCT116-DoxR cell line (0.35 μM compared to 0.24 for complex **1** and 0.33 μM compared to 0.23 μM for complex **2**, respectively). This is an impressive cytotoxic activity against the resistant cell line, particularly if one considers its associated mechanism of resistance (PgP overexpression)^53^ that hampers the intracellular accumulation of the agent. Importantly, complexes’ IC_50_ values in both CRC cell lines are below the respective IC_50_ values presented for the antitumor drugs Cis, Dox, and FF^53^ (Table 1 and Figures S8 and S9), highlighting the significant potential for the application of both copper­(II) complexes as antiproliferative agents in CRC cell lines.

In novel drug design and validation, it is important to ensure that the complexes do not pose harm to normal cells (toxic side effects). This is measured through the comparison of the IC_50_ values in both tumor and healthy cell lines, such as normal primary fibroblasts (Table 1).^54^ This normal cell line was chosen due to its importance in tissues and in the TME. The selectivity index (SI), the ratio between of relative IC_50_ in fibroblasts and tumor cells, was calculated to determine the tumor specificity of each complex in each cell line (Table 1).

Both complexes presented remarkable SI values in both cancer cell lines. For HCT116 cells, the SI values were 138 for complex **1** and 174 for complex **2**, confirming strong preferential activity toward cancer cells. Similar trends were observed in the HCT116-DoxR cell line, with SI values of 95 for complex **1** and 122 for complex **2**, indicating that both complexes retain efficacy even in a drug-resistant background.

The pronounced antiproliferative potential of the newly synthesized Cu­(II) complexes against HCT116 and HCT116-DoxR cell lines is also supported by a comparative analysis of their IC_50_ and SI values with those previously reported for terpyridyl coordination compounds.^10–13,30,32,33^ As illustrated in Figure 4, coordination of tmp-terpy to Cu­(II) yields coordination compounds exhibiting anticancer activity comparable to that of [AuCl­(tmp-terpy)]­(PF_6_)_2_ and markedly enhanced relative to [PtCl­(tmp-terpy)]­(CF_3_SO_3_) and [MnCl_2_(tmp-terpy)].^30^ Within the family of terpyridyl Cu­(II) coordination compounds investigated as agents against colorectal cancer, the newly synthesized Cu­(II) complexes, together with [CuCl_2_(4′-(4-methoxynaphthalen-1-yl)-2,2′:6′,2″-terpyridine)][Bibr ref13] and [Cu_2_Cl_2_(4′-(quinolin-4-yl)-2,2′:6′,2″-terpyridine)_2_]­(PF_6_)_2_,^11^ exhibit significantly higher cytotoxic activities than other potent terpyridyl copper-based metallodrugs reported to date for colorectal cancer therapy.^10–13,32,33^

**Figure 4:**
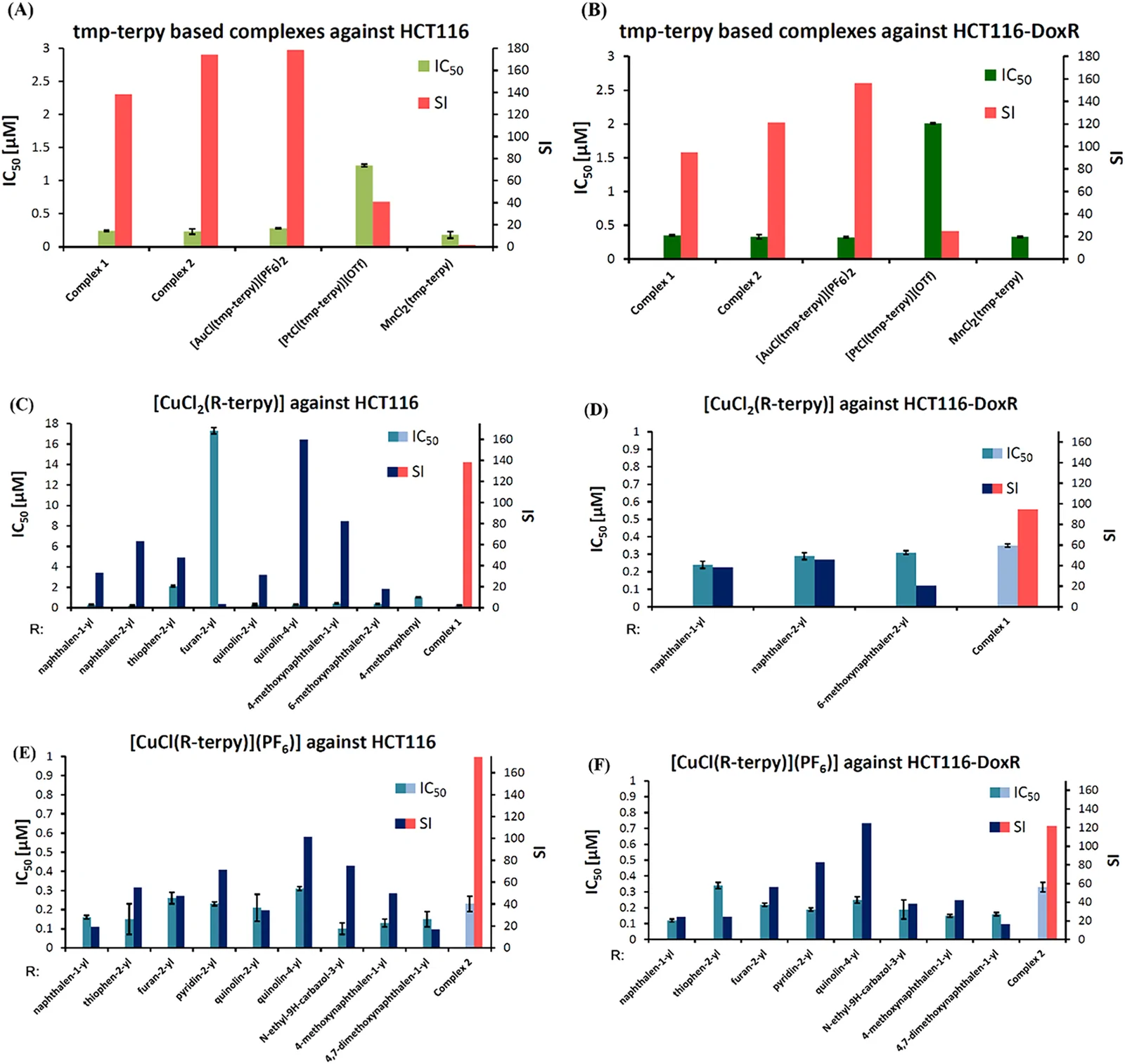
Comparison of IC_50_ and SI values of the tmp-terpy containing coordination complexes against HCT116 (A) and HCT116-DoxR (B). Comparison of IC_50_ and SI values of the Cu­(II) coordination complexes with 4′-R-2,2′:6′,2″-terpyridines (R-terpy) against HCT116 (C and E) and HCT116-DoxR (D and F).^10–13,30,32,33^

When compared with the controls Cis and Dox, the Cu­(II) complexes were also demonstrated to be far more selective than Cis and Dox in both studied cell lines. However, when compared with FF, Cu­(II) complexes are more selective for the HCT116 cell line but less selective in HCT116-DoxR cells (Table 1). Despite the difference in the SI values, with complex **2** showing a higher SI value, both Cu­(II) complexes exhibit similar IC_50_ values. We next evaluated the cytotoxic activity of both complexes in more complex 3D models.

#### 3D Spheroids Cell Viability Assays

To comparatively evaluate the cytotoxic activity of the copper­(II) complexes in 2D and 3D culture systems, CellTiter-Glo assays were conducted on 6-day-old HCT116 and HCT116-DoxR spheroids. Treatment with FF or the Cu­(II) compounds was performed for 48 h (Figures 5 and S10). Table 2 compares the IC_50_ values of the complexes in 2D and 3D cultures across both cell lines.

**Figure 5:**
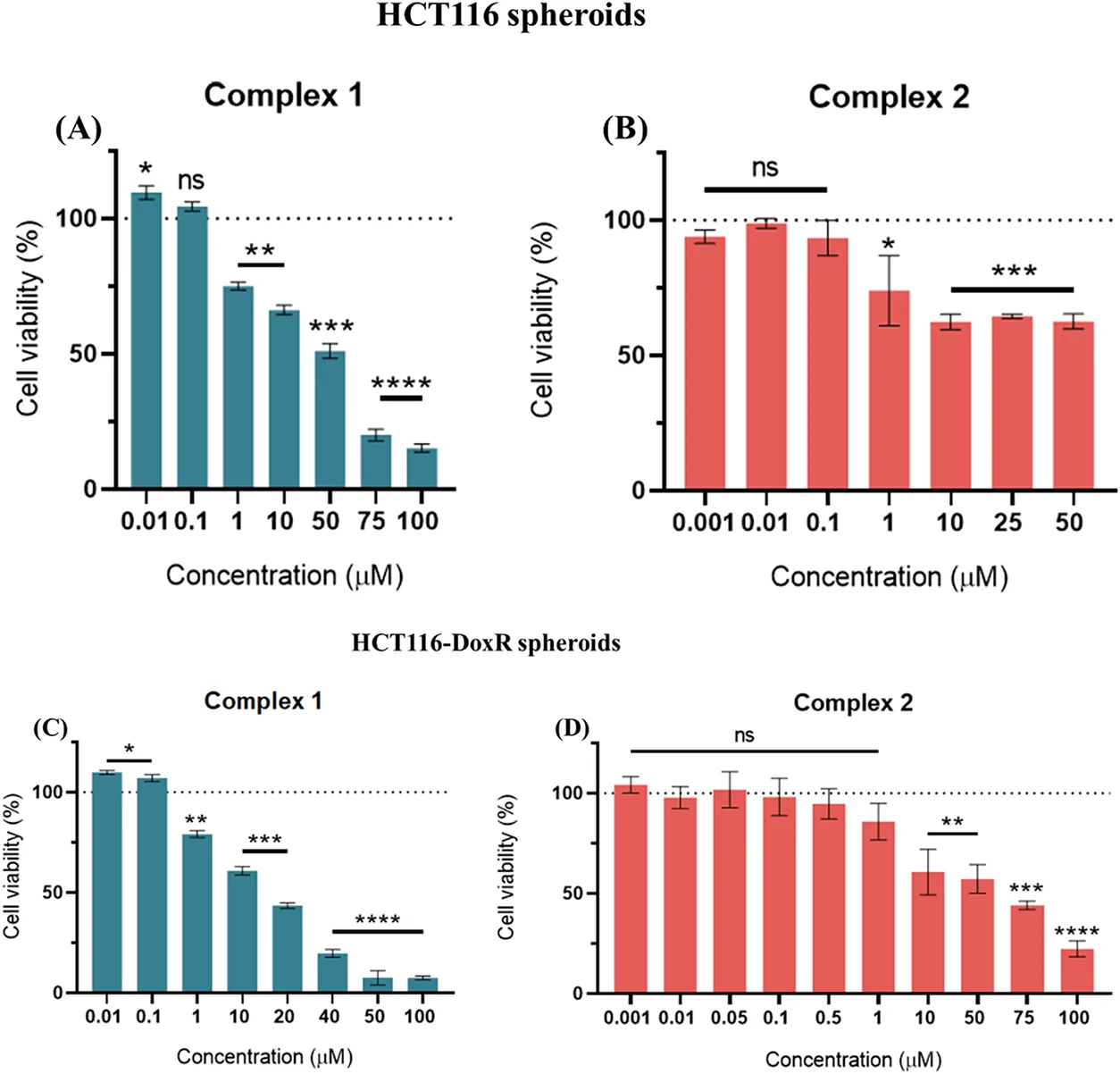
Cell Viability of 6-day-old HCT116 (A and B) and HCT116-DoxR (C and D) spheroids after exposure to different concentrations of complexes **1** and **2** for 48 h. The vehicle control, at the same final concentration used for the complexes, is represented as a dashed line. Data are presented as mean ± SEM from a minimum of three independent biological replicates. Statistical significance relative to the DMSO control was determined using one-way ANOVA (ns, not significant; *p < 0.05; **p < 0.01; ***p < 0.001; ****p < 0.0001).

**Table 2:** Comparison of the Relative IC_50_ Values Obtained for FF and Complexes **1** and **2** for 2D and 3D HCT116 and HCT116-DoxR Cell Lines^a^

		relative IC_50_ (μM)	
complex	cell line	2D	3D
**1**	HCT116	0.24 ± 0.01	17.48 ± 0.08
	**HCT116-DoxR**	**0.35 ± 0.01**	**5.78 ± 0.07**
**2**	HCT116	0.23 ± 0.04	>50
	HCT116-DoxR	0.33 ± 0.03	15.80 ± 0.11
FF	HCT116	2.79 ± 0.08	2302 ± 0.25
	HCT116-DoxR	1.35 ± 0.06	>1600

a IC_50_ values are expressed as the mean ± SEM from a minimum of three independent biological replicates. The reported values for FF reflect the IC_50_ of 5-FU since it is the major component of FF. For the lasting FF components, their concentration was calculated based on the proportions: 1 5-FU: 0.042 FA: 0.012 OX.

As shown in Table 2, IC_50_ values in 3D cultures were higher than those obtained in 2D monolayers, likely reflecting the lower diffusion and penetration of the complexes within the more complex spheroid architecture.^55^ Because diffusion toward the inner core is influenced by spheroid size, 6-day-old spheroids (750 μm) were used in all assays to ensure consistent and controlled dimensions across experiments.^25^

In the case of complex **1**, the IC_50_ in 3D spheroids increased by 73x in HCT116 and 17x in HCT116-DoxR spheroids compared with 2D cultures. In contrast, complex **2** showed an even more pronounced shift, with IC_50_ values at least 232x higher in HCT116 and 48x in HCT116-DoxR spheroids. Notably, although complex **2** exhibited slightly higher cytotoxicity in 2D cultures, particularly for HCT116-DoxR 3D spheroids, complex **1** displayed superior cytotoxic activity in this 3D tumor spheroid model.

These results (Table 2) agree with previous reports,^11^ which show that higher concentrations in 3D spheroids are required to attain the same cytotoxic effects in 2D cultures. Notably, within the family of terpyridyl Cu­(II) coordination compounds investigated with the use of HCT116-DoxR 3D models, complex **1** shows the lowest IC_50_ value (Table 3).

**Table 3:** IC_50_ Values in HCT116-DoxR 3D Models of the Cu­(II) Terpyridine Complexes

compound	IC_50_ (μM) HCT116-DoxR 3D model	ref.
**1**	**5.78 ± 0.07**	this work
**2**	15.80 ± 0.11	this work
CuCl_2_(4′-(naphthalen-1-yl)-2,2′:6′,2″-terpyridine)	10.10 ± 0.22	^10^
CuCl_2_(4′-(naphthalen-2-yl)-2,2′:6′,2″-terpyridine)	33.86 ± 0.40	^10^
[Cu_2_Cl_2_(4′-(furan-2-yl)-2,2′:6′,2″-terpyridine)_2_](PF_6_)_2_	13.97 ± 0.60	^11^
[Cu_2_Cl_2_(4′-(pyridin-2-yl)-2,2′:6′,2″-terpyridine)_2_](PF_6_)_2_	30.48 ± 0.19	^11^
[Cu_2_Cl_2_(4′-(quinolin-4-yl)-2,2′:6′,2″-terpyridine)_2_](PF_6_)_2_	15.25 ± 0.80	^11^
[Cu_2_Cl_2_(4′-(methoxynaphthalen-1-yl)-2,2′:6′,2″-terpyridine)_2_](PF_6_)_2_	6.35 ± 0.12	^11^

Moreover, complex **1** exhibits lower IC_50_ values in 3D models than commonly used regimens such as FF, suggesting its potential as an alternative therapeutic approach or even as a potential combination with the FF regimen. On the basis of its superior cytotoxic activity in both cell lines, and particularly in the HCT116-DoxR model cultured as 3D spheroids, complex **1** was selected for further studies. Owing to its greater clinical relevance in the context of resistance to drugs leading to therapy failure, the HCT116-DoxR cell line was selected for subsequent assays to evaluate whether the complex could circumvent Dox-associated efflux and resistance mechanisms and engage alternative pathways.

We next evaluated the cytotoxic effect of complex **1**, applied at its IC_50_ in combination with the FF regimen in HCT116-DoxR 3D tumor spheroids, and compared it to that of each treatment alone (Figure 6). To minimize toxicity to healthy cells and address limitations of current CRC regimens, we used FF at its IC_10,_ determined from preliminary dose–response data (Figure S10). At this concentration, we ensure a measurable biological effect while potentially enabling complementary mechanisms of action and highlighting possible synergistic effects with our compounds. Viability of HCT116-DoxR spheroids was assessed after 48 h of exposure to complex **1** (at its IC_50_), alone or in combination with FF at IC_10_ (Figure 6).

**Figure 6:**
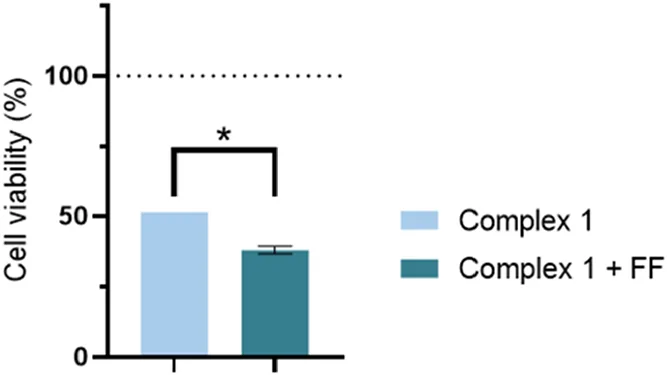
Cell Viability of 6-day-old HCT116-DoxR spheroids after exposure to complex **1** (IC_50_) and complex **1** (IC_50_) + FF (IC_10_) for 48 h. The vehicle control (DMSO or DMSO + glucose, since the complex and 5-FU are dissolved in DMSO and OX is dissolved in a 5% glucose solution), at the same final concentration used for the complexes, is represented as a dashed line. Data are presented as mean ± SEM from a minimum of two independent biological replicates. Statistical significance relative to DMSO control was determined using one-way ANOVA (*p < 0.05).

The combination of complex **1** with IC_10_ FF reduced cell viability by approximately 26% compared to that of complex **1** alone, suggesting an enhanced cytotoxic effect of the combined treatment (Figure 6). These findings support the hypothesis that novel metal complexes could be combined with established chemotherapeutic regimens or potentially replace specific components of these treatments, such as the platinum-based agent OX.

#### 3D Patient-Derived Organoid (PDO) Cell Viability Assays

Even though the 3D spheroids’ cell viability gives a good picture of the ability of the complexes to penetrate within these structures and their antiproliferative potential in more complex models, the spheroids used in this study are homotypic—composed of a single cell type—and derived from immortalized cell lines, which may not fully recapitulate patient heterogeneity.^2,22^ Therefore, the CellTiter-Glo assay was also performed on 4-day-old PDOs from normal and tumor tissues following 48 h of exposure to FF and Cu­(II) complexes (Figures 7 and S11). In this case, to gain a better understanding of both complex cytotoxicities in PDOs, we also included complex **2** in this analysis.

**Figure 7:**
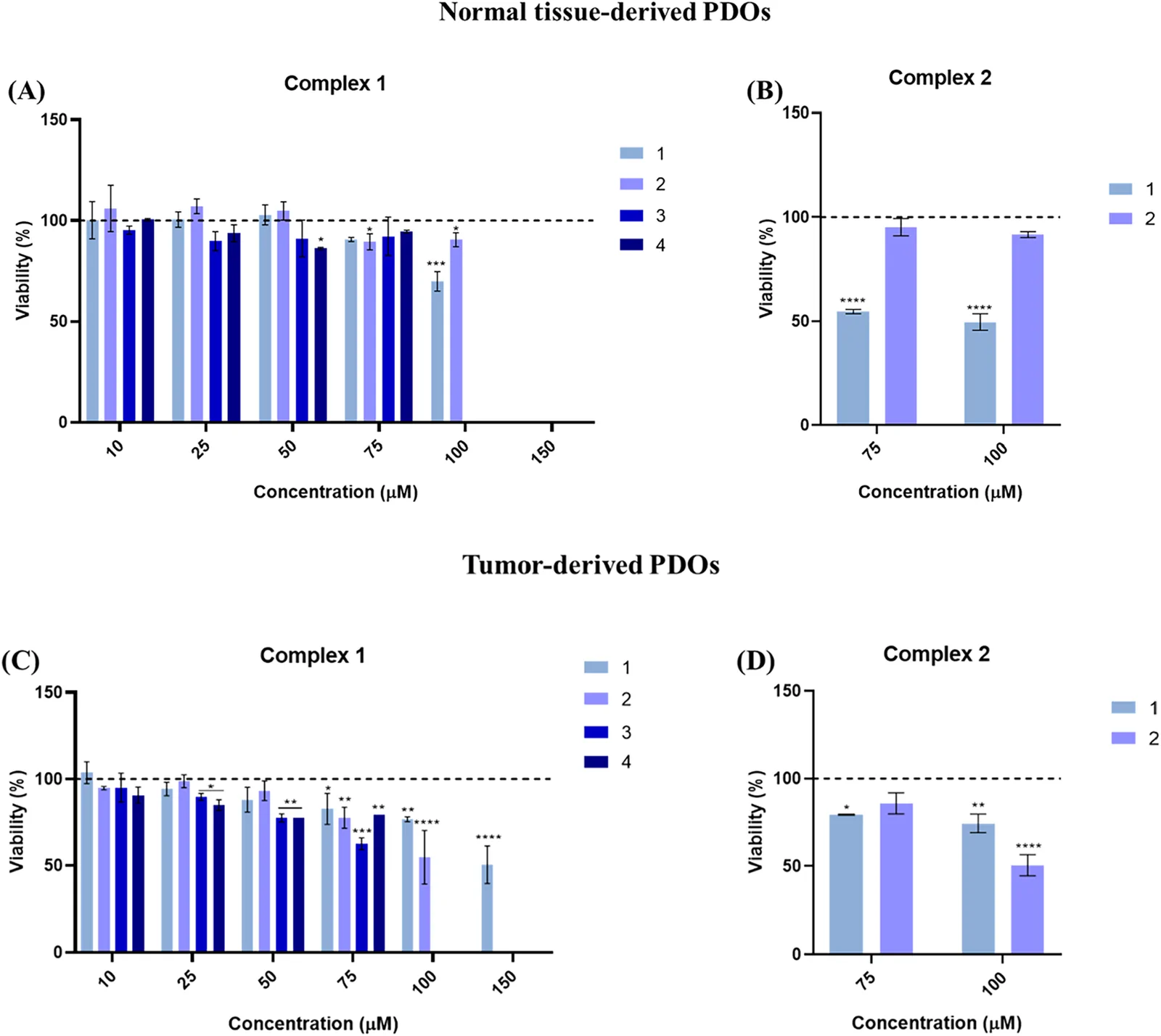
Cell Viability of four pairs of 4-day-old PDOs from normal tissue (A and B) and tumor tissue (C and D) after exposure to different concentrations of complexes **1** and **2** for 48 h. The vehicle control, DMSO, at the same final concentration used for the complexes, is represented as a dashed line. Data are presented as mean ± SEM from a minimum of three independent biological replicates. Statistical significance relative to DMSO control was determined using one-way ANOVA (*p < 0.05; **p < 0.01; ***p < 0.001; ****p < 0.0001).

First, it is important to note that PDOs were organized into four pairs: three pairs (pairs 1–3) derived from normal and tumor tissues from the same patient and one pair (pair 4) derived from normal and tumor tissues from different patients (see more details in Table S10). Overall, the concentration of complexes to obtain similar antiproliferative effects as those observed in 2D cultures and in 3D spheroids drastically increases (Figure 7), which may be attributed to the higher complexity of these models that pose limitations to the diffusion within a dense extracellular matrix (ECM) of Matrigel.^2,22,55,56^ When analyzing data from Figures 7 and S11, these PDO models recapitulate clinically observed phenomena, including heterogeneous patient responses to treatment arising from genetic and epigenetic variability.^29^ Such heterogeneous responses were evident upon treatment with complexes **1** and **2**, as well as with the FF regimen.

Regarding the cytotoxic effect of complex **1** in PDOs derived from normal tissues (Figure 7A), no significant reduction in viability was observed in any of the four pairs up to 75 μM (viability >90%), indicating that complex **1** is not cytotoxic to healthy intestinal cells at these concentrations. Remarkably, when tumor-derived PDOs are exposed to concentrations of complex **1** higher than or equal to 50 μM, a statistically significant decrease in cell viability is observed (Figure 7C). Collectively, these observations suggest that complex **1** may exhibit a therapeutic window, in which cell viability is reduced in tumor-derived PDOs while sparing PDOs derived from normal tissue. On the other hand, a concentration of complex **2** of 75 μM induces high cytotoxicity in PDOs 1 (derived from normal tissue), reducing its cell viability to 50% (Figure 7B). However, like complex **1**, complex **2** did not exhibit significant toxicity in normal PDOs 2. In tumor-derived PDOs, complex **2** exhibited effects comparable to those of complex **1** at concentrations above 50 μM (Figure 7D). Nevertheless, the cytotoxicity observed in organoids reduces the therapeutic window for this complex since it affects more healthy intestinal tissues than the tumor tissue. We then analyzed the effect of the FF regimen in PDOs. Except for pair 4 (which is not derived from the same patient), a strong reduction in viability was observed, demonstrating its antitumor potential; nevertheless, heterogeneous responses among individual patients were also observed (Figure S11). For instance, normal PDO 3 is much more sensitive to the FF regimen compared to the other normal PDOs.

Building on these results, the combination of complex **1** with FF (IC_10_) was also tested in PDOs. In this experiment, PDOs were treated with the IC_10_ of FF in combination with varying concentrations of complex **1** for 48 h, and cell viability was subsequently assessed (Figure 8).

**Figure 8:**
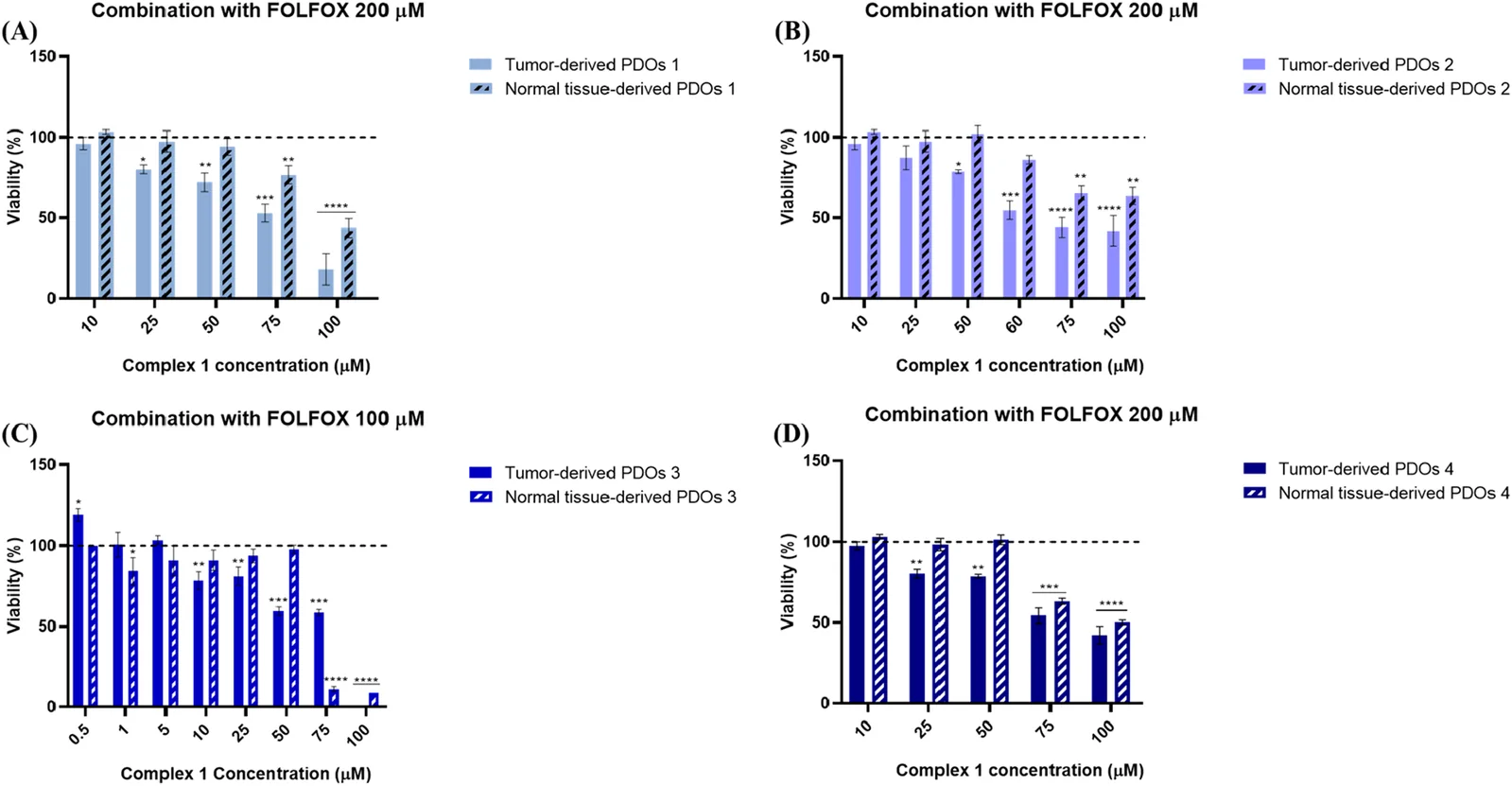
Cell Viability of 4-day-old pairs of PDOs derived from normal and tumor tissues (1 – A; 2 – B; 3 – C; and 4 – D) after exposure to complex **1** + FF (IC_10_) for 48 h. The vehicle control (DMSO + glucose, since the complex and 5-FU are dissolved in DMSO and OX is dissolved in a 5% glucose solution), at the same final concentration used for the complexes, is represented as a dashed line. Data are presented as mean ± SEM from a minimum of three independent biological replicates. Statistical significance relative to DMSO control was determined using one-way ANOVA (*p < 0.05; **p < 0.01; ***p < 0.001; ****p < 0.0001).

Combining FF at IC_10_ with complex **1** enhances the antitumor capability (Figure 8). Indeed, concentrations of 50–60 μM of complex **1** in combination with FF were sufficient to reduce tumor-derived PDOs’ viability, without significantly affecting normal PDOs (viability >80%). In contrast, when using complex **1** alone, concentrations of 100–150 μM were needed to achieve a comparable reduction in tumor-derived PDOs viability. It should be noted that combinations above 75 μM of complex **1** induced substantial cytotoxicity in normal PDOs (Figure 8).

These results emphasize the heterogeneous responses of PDOs derived from different patients, highlighting the need for more personalized treatment strategies rather than a uniform dosing or therapeutic approach – Precision Therapy. Indeed, patient 1 has a metastatic colorectal tumor that does not respond well to the FF regimen (Figure S11) or to complex **1** alone (Figure 7) but is more responsive to the combination scheme (Figure 8). For instance, in patient 3, with a grade 4 tumor (but no metastasis), a good response to the FF regimen and complex **1** individually, as well as to their combination, is observed (Figures 7 and 8 and S11).

Collectively, these findings highlight the potential benefit of using PDOs to assess the anticancer properties of novel complexes and the advantage of combinatory regimens of novel chemotherapeutic agents leveraging on established clinical regimens to improve selectivity and reduce toxicity.

Considering all of the results presented above, complex **1** emerged as the most promising of the two copper­(II) complexes and was therefore selected for further biological studies to elucidate its mechanism of action. Although 3D models are considered to be more physiologically relevant, subsequent biological assays were conducted using two-dimensional (2D) monolayer cultures due to methodological simplicity and experimental feasibility.

#### Evaluation of Apoptosis Induction

To gain insight into the mechanisms responsible for the complex’s cytotoxic activity, cell death pathways were first explored. Apoptosis in HCT116-DoxR 2D cultures was assessed by flow cytometry following dual staining with Alexa Fluor 488–Annexin V and PI. In viable cells, phosphatidylserine (PS) is confined to the inner leaflet of the plasma membrane; however, during early apoptosis, PS is translocated to the outer leaflet, where it binds Annexin V. Consequently, this method allows for the distinction of viable (Annexin V–/PI−), early apoptotic (Annexin V+/PI−), late apoptotic (Annexin V+/PI+), and necrotic (Annexin V–/PI+) cell populations.^54,57^

Figure 9 and Table S11 present the results obtained after 48 h of treating 2D HCT116-DoxR cells with the IC_50_ (in 2D) of complex **1** alone, or the IC_10_ (in 2D) of FF alone, or their combination. DMSO and the FF vehicles were used as negative controls, and 5 μM Cis was used as a positive control.

**Figure 9:**
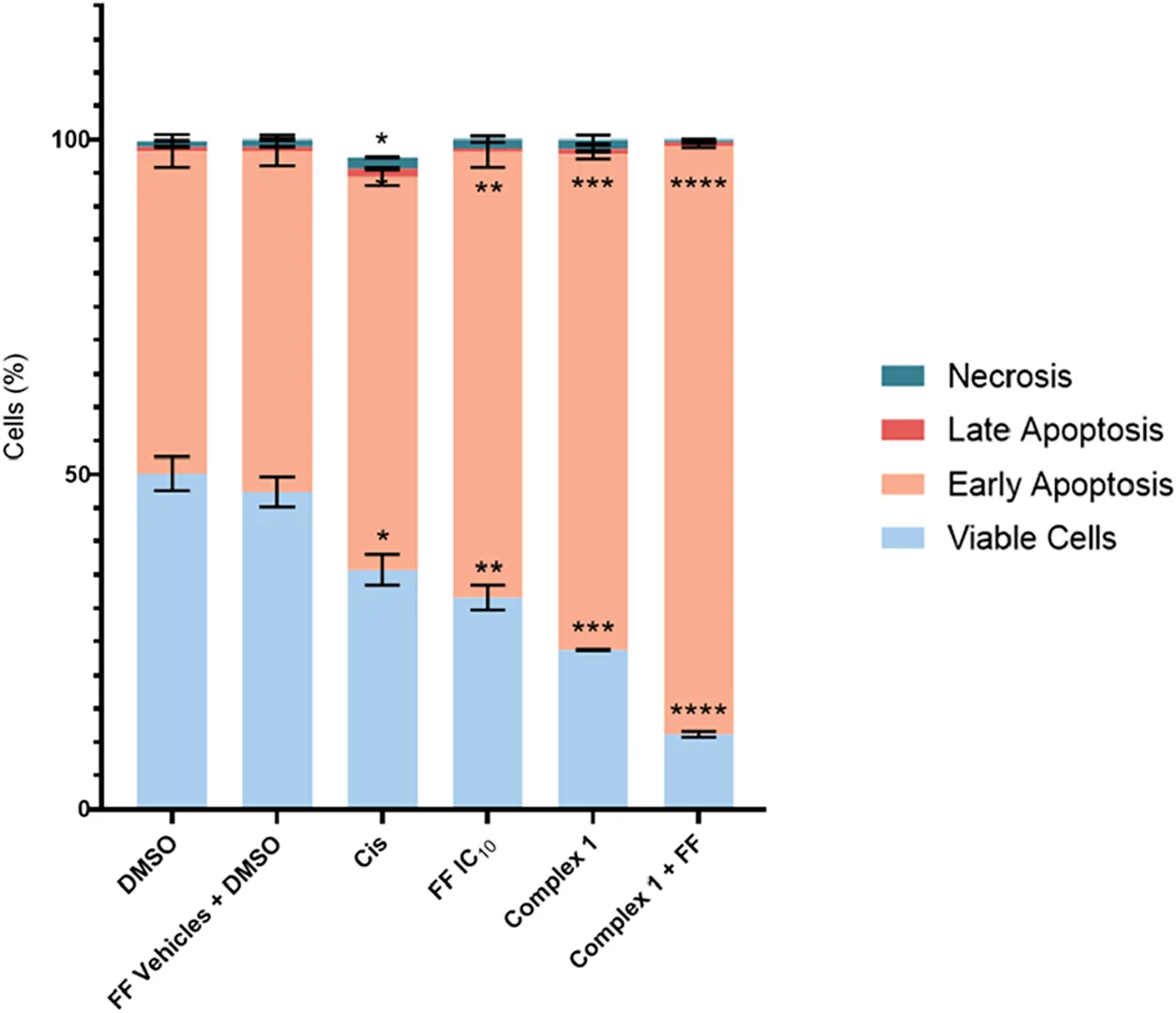
Percentage of viable (light blue bars), early apoptotic (coral bars), late apoptotic (red bars), and necrotic (dark blue bars) HCT116-DoxR cells cultured in 2D, after 48 h exposure to complex **1** (IC_50_ in 2D models), FF (IC_10_ in 2D models), and their respective combinations. DMSO and the corresponding FF vehicle controls were used as negative controls, while Cis (5 μM) served as a positive control. Data are presented as mean ± SEM from a minimum of two independent biological replicates. Statistical significance relative to the control was determined by one-way ANOVA (*p < 0.05; **p < 0.01; ***p < 0.001; ****p < 0.0001).

As shown in Figure 9 and Table S11, FF and Cis induced approximately 60% apoptosis in HCT116-DoxR cells, whereas complex **1**—either alone or combined with FF—resulted in markedly higher levels (70–90%). The DMSO control yielded ∼50% viability, 49% apoptosis, and 0.9% necrosis, and the mixture of FF vehicles with DMSO produced a similar profile (48% viability, 51% apoptosis, 1% necrosis). The viability obtained for the DMSO control was lower than expected based on Choroba et al.^54^ This discrepancy may arise from differences in passage numbers, which can influence basal apoptosis; however, values were always normalized to vehicle controls.

After normalization to the corresponding controls, Cis and FF increased apoptosis by ∼1.2× and 1.3×, respectively. By comparison, complex **1** induced a 1.5-fold increase relative to the DMSO control and 1.2- and 1.1-fold increases relative to cisplatin and FF, respectively. Notably, the combination of complex **1** and FF resulted in a 1.7-fold increase compared with the vehicle control, as well as 1.2- and 1.3-fold increases relative to complex **1** or FF alone, respectively. The complex and its combination with FF also induced low necrosis levels (1.31 and 0.34%, respectively), which were lower than the values observed for Cis control (1.72%) and FF alone (1.33%). These findings indicate that complex **1** robustly induces apoptotic cell death, consistent with previous reports on other copper complexes,^11,58^ and that cotreatment with FF further enhances this effect. The ability of both copper complexes and FF to promote apoptosis has been well documented in the literature.^59^

#### Mitochondrial Membrane Potential (ΔΨ_m_) Evaluation

To gain further insight into the apoptotic pathway triggered by the complex and its combination with FF, mitochondrial membrane potential (ΔΨ_m_) was analyzed in HCT116-DoxR cells grown in 2D. Changes in ΔΨ_m_ can be quantified and represent a key indicator of mitochondrial functional integrity. Thus, the fluorescent probe JC-1 (5,5′,6,6′-tetrachloro-1,1′,3,3′-tetraethylbenzimidazolylcarbocyanine iodide) was employed to monitor these modifications. In cells with preserved mitochondrial membrane potential (ΔΨ_m_), JC-1 accumulates within mitochondria as red-fluorescent aggregates, whereas loss of ΔΨ_m_ leads to the formation of green-fluorescent JC-1 monomers in the cytoplasm.^54^ Mitochondrial status was assessed by flow cytometry through the red/green fluorescence ratio, with ratios exceeding 1 as indicative of hyperpolarization, and ratios <1 as indicative of depolarization. As illustrated in Figure 10, cells were treated with the 2D IC_50_ of complex **1**, the 2D IC_10_ of FF, or their combination for 48 h before JC-1 staining.

**Figure 10:**
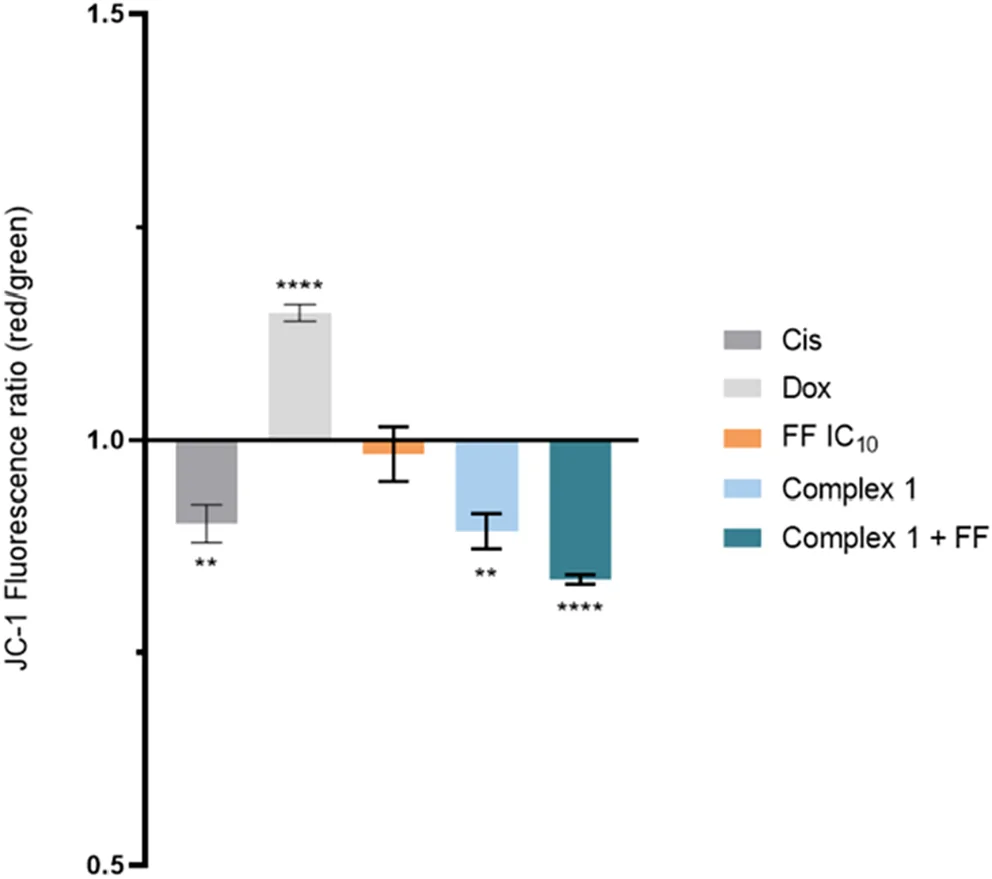
JC-1 fluorescence ratio (red/green) in HCT116-DoxR cells after 48 h exposure to complex **1** (2D IC_50_), FF (2D IC_10_), and their respective combination. Cis (5 μM) and Dox (6 μM) were used as positive controls, while DMSO and FF vehicle controls (at the same final concentration as the treatments) served as negative controls. Results were normalized to their respective vehicle controls. Data are presented as mean ± SEM from a minimum of two independent biological replicates. Statistical significance relative to the control was determined by one-way ANOVA (*p < 0.05; **p < 0.01; ****p < 0.0001).

All tested conditions show the induction of mitochondrial membrane depolarization, except for Dox, likely due to interference with its own fluorescence, as previously reported^11,60^ (Figure 10). Treatment with complex **1** decreased ΔΨ_m_, consistent with other studies,^61,62^ suggesting activation of the mitochondrial apoptotic pathway or both pathways (intrinsic and extrinsic) through BID cleavage.^63^ FF alone caused minimal depolarization, likely due to its low concentration and the fact that its components, 5-FU and OX, primarily target DNA.^64–66^ Notably, the combination of complex **1** with FF resulted in greater depolarization than the complex alone, indicating that FF potentiates its effect. Given that mitochondria are the primary source of cellular ATP and a major site of ROS production,^59^ evaluating ROS generation in the presence of these complexes is essential for further elucidating their mechanism of action.

#### Evaluation of ROS Production

Metal-based complexes can induce cytotoxic effects through the generation of ROS, which, when present at high intracellular levels, trigger apoptosis and autophagy.^67^ To assess the production of ROS through flow cytometry, the probe H_2_DCFDA was used. Once inside the cell, H_2_DCFDA (2′,7′-dichlorohydrofluorescein diacetate) is deacetylated by intracellular esterases to a nonfluorescent form, which is subsequently oxidized by ROS into the fluorescent molecule DCF (2′,7′-dichlorofluorescein), thereby enabling detection of intracellular ROS levels.^11^ Figure 11 shows the data obtained after 48 h of exposure of HCT116-DoxR cells to the 2D IC_50_ of complex **1**, the 2D IC_10_ of FF, or their combinat**i**on, followed by staining with H_2_DCFDA. Positive controls included 5 μM Cis, 6 μM Dox, and 50 μM H_2_O_2_, whereas DMSO and the FF vehicle acted as negative controls.

**Figure 11:**
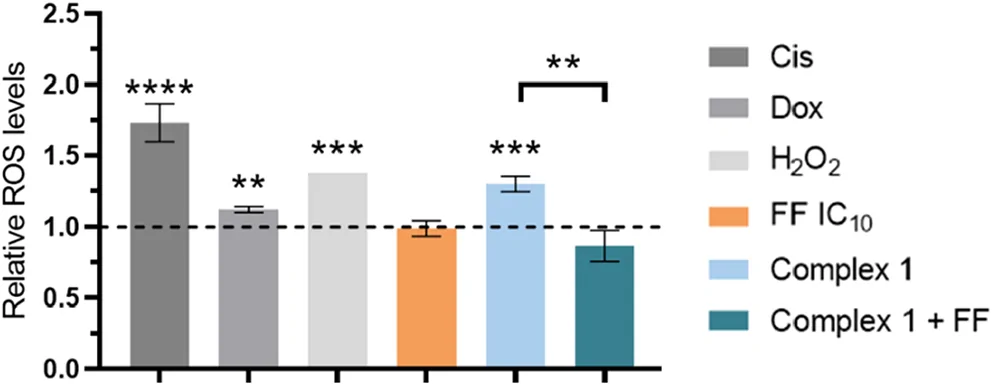
ROS production in HCT116-DoxR cells after 48 h exposure to complex **1** (2D IC_50_), FF (2D IC_10_), and the respective combination. Cis (5 μM), Dox (6 μM), and H_2_O_2_ (50 μM) were used as positive controls, while DMSO and FF vehicle controls (at the same final concentration as the treatments) served as negative controls. Results were normalized to their respective vehicle controls, represented by the dashed line. Data are presented as mean ± SEM from a minimum of two independent biological replicates. Statistical significance relative to the control was determined by one-way ANOVA (ns, not significant; **p < 0.01; ***p < 0.001; ****p < 0.0001).

Complex **1** alone can trigger ROS production by approximately 1.3-fold relative to the control (Figure 11). Although these values are lower than those observed for the cisplatin control (≈1.75-fold relative to the corresponding control), they are comparable to the levels of ROS production induced by the positive control H_2_O_2_ and exceed those elicited by doxorubicin (≈1.2-fold relative to the corresponding control). In contrast, the FF regimen can maintain ROS levels close to baseline, likely due to its low tested concentration, IC_10_. Notably, complex **1** induced ∼1.3-fold higher ROS levels than FF alone; however, their combination reduced ROS below basal levels (0.87-fold higher than the respective control) and resulted in lower ROS production than the complex alone. These findings may be the reflection of the distinct roles of its components, as OX and 5-FU can promote ROS generation, whereas FA primarily modulates 5-FU activity and has been associated with enhanced glutathione synthesis, potentially limiting oxidative stress.^68^ Given the low FA concentration used, this effect may be modest, and alternative explanations, such as probe interference or fluorescence quenching, cannot be excluded.^69^

Overall, the elevated ROS levels induced by complex **1** alone are in line with previous studies^11,60^ and may contribute to the activation of apoptotic pathways observed in Figures 9 and 10.

#### Autophagy Induction

To determine if the copper complex triggers additional cell death pathways beyond apoptosis, autophagy—a programmed cell death characterized by the sequestration of cellular components into double-membrane autophagosomes—was investigated.^70^ HCT116-DoxR cells underwent 48 h of treatment with either the 2D IC_50_ of complex **1**, the 2D IC_10_ of FF, or their combination. Negative controls consisted of DMSO and FF vehicles, while 5 μM Cis, 6 μM Dox, and 1.5 μM rapamycin were included as positive controls. Cells were subsequently stained with a green autophagosome fluorescent marker (ab139484, Abcam, Cambridge, UK). The results are presented in Figure 12.

**Figure 12:**
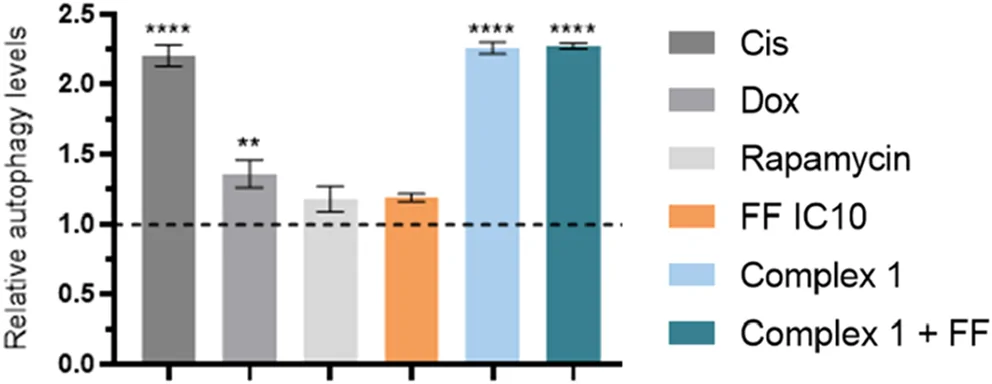
Autophagy induction in HCT116-DoxR cells after 48 h exposure to complex **1** (2D IC_50_), FF (2D IC_10_), and the respective combination. DMSO and FF vehicle controls (at the same final concentration as the treatments) were used as negative controls, while Cis (5 μM), Dox (6 μM), and rapamycin (1.5 μM) served as positive controls. Results were normalized to their respective vehicle controls, represented by the dashed line. Data are presented as mean ± SEM from a minimum of two independent biological replicates. Statistical significance relative to the control was determined by one-way ANOVA (ns, not significant; *p < 0.05; ***p < 0.001; ****p < 0.0001).

Data show autophagy induction by Cis and Dox (positive controls), whereas rapamycin did not, possibly due to the short incubation period (Figure 12). FF treatment produced a modest increase in autophagy (1.2-fold) relative to the DMSO vehicle. Although both 5-FU and OX have been reported to trigger autophagy in CRC cells,^71,72^ the low concentration used (IC_10_) likely explains the minimal effect, consistent with its inability to induce ROS (Figure 11). In contrast, complex **1** markedly increased autophagy by ∼2.3-fold relative to DMSO, in agreement with previous reports for other Cu­(II) complexes.^11^

The combination of complex **1** and FF did not show an increase in autophagy levels compared with complex **1** alone, suggesting that the potential synergistic effect is mostly observed in the capability to induce apoptosis (Figure 9). These findings indicate that complex **1** induces HCT116-DoxR cell death via both apoptosis and autophagy, which trigger ROS, consistent with mechanisms reported for other copper complexes,^12,73^ highlighting its ability to trigger multiple cytotoxic pathways in tumor cells. Nevertheless, despite the potential synergistic effect of their combination on cell viability (Figures 6, 7, and 8) and apoptosis (Figures 9 and 10), ROS and autophagy are not additionally promoted compared with complex **1** alone (Figures 11 and 12), which may reflect the attainment of a saturation threshold.

#### Cell-Cycle Progression

Many metallodrugs are capable of interfering with cell-cycle progression, often exerting cytostatic effects that contribute to cell death.^74^ Accordingly, the impact of complex **1** and its combination with FF on cell-cycle distribution was examined by flow cytometry following staining with PI, a DNA-intercalating dye, that allows quantification of DNA content and discrimination between the G_0_/G_1_, S, and G_2_/M phases. To ensure cell-cycle synchronization before treatment, HCT116-DoxR cells were subjected to a double thymidine block at the S phase, which arrests cells by inhibiting DNA synthesis.^11,54^ After synchronization, cells were exposed to the 2D IC_50_ of complex **1**, the 2D IC_10_ of FF, or their combination for 9, 18, and 24 h. DMSO and Dox (6 μM) were used as negative and positive controls, respectively.

As shown in Figure 13, Dox induced a transient delay at 9 h, with cells accumulating in G_2_/M, followed by partial recovery at 18 h and a return to G_2_/M by 24 h, which was expected, since the used cells are resistant to Dox. FF, even at IC_10_, produced a similar pattern, supporting its cytostatic activity in the HCT116-DoxR cells. Complex **1** induced a stronger and earlier cell-cycle blockade at 9 h, with a higher proportion of cells retained in the S phase compared with the DMSO control, indicating delayed cell-cycle progression. This effect persisted at 18 h, with cells showing a slight transition to G_2_/M and only partial recovery by 24 h. Relative to Dox, complex **1** induced a more pronounced and sustained delay in cell-cycle progression. When combined with FF, complex **1** continued to delay the cycle, preventing progression to G_2_/M at 9 h; however, the combination did not markedly enhance the effect of the complex alone, apart from a slightly stronger delay at 24 h, where cells remained predominantly in the G_2_/M phase (Figure 13). This shows that their combination and the complex alone display a cytostatic effect, delaying normal cell-cycle progression in both the S and G_2_/M phases, magnifying the advantages of the use of complex **1** either alone or in association with FF.

**Figure 13:**
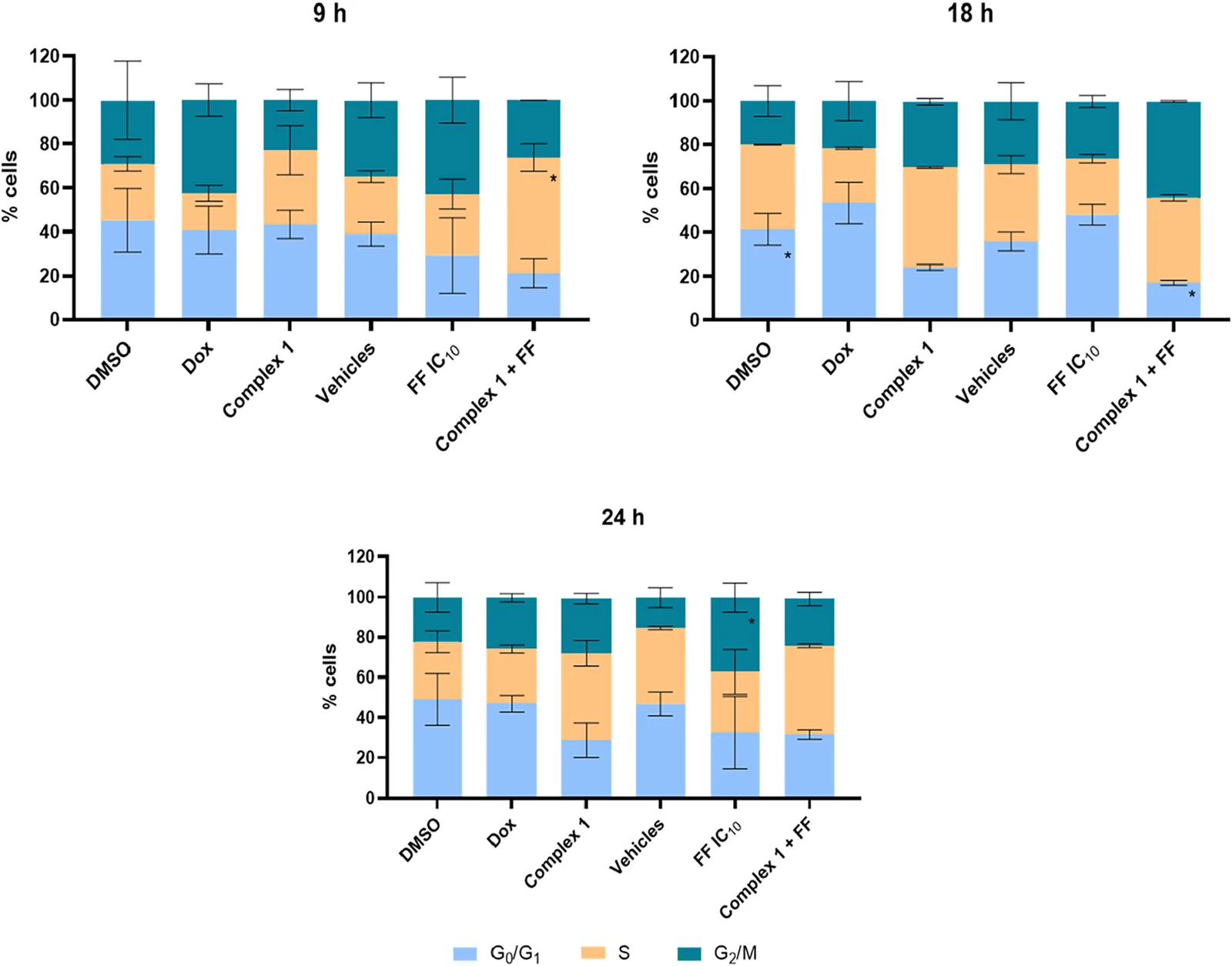
HCT116-DoxR cells’ cell-cycle distribution after 9, 18, and 24 h of treatment with complex **1** (2D IC_50_) and FF (2D IC_10_). DMSO and FF vehicle controls (at the same final concentration as the treatments) were used as negative controls, while Dox (6 μM) served as the positive control. Data are presented as mean ± SEM from a minimum of three independent biological replicates. Statistical significance relative to the control was determined by one-way ANOVA (*p < 0.05).

#### Antiangiogenic Capability

The capability to inhibit angiogenesis—the generation of new blood and lymphatic vessels from existing vasculature^75^—is a key feature in circumventing cancer development, as this approach reduces nutrient and oxygen supply, disables metabolic waste clearance, and hampers metastatic cell migration.^76^ To evaluate whether the Cu­(II) complex interferes with angiogenesis, the *ex-ovo* chorioallantoic membrane (CAM) assay was employed. Chick CAM is commonly used due to its dense network of capillaries and role in gas exchange.^77^ O-rings were positioned in vascularized areas of the membrane, followed by application of either the 3D IC_50_ of complex **1** or the 3D IC_10_ of FF, with DMSO in PBS as a negative control. Images were acquired at 0, 24, and 48 h postexposure (Figure 14).

**Figure 14:**
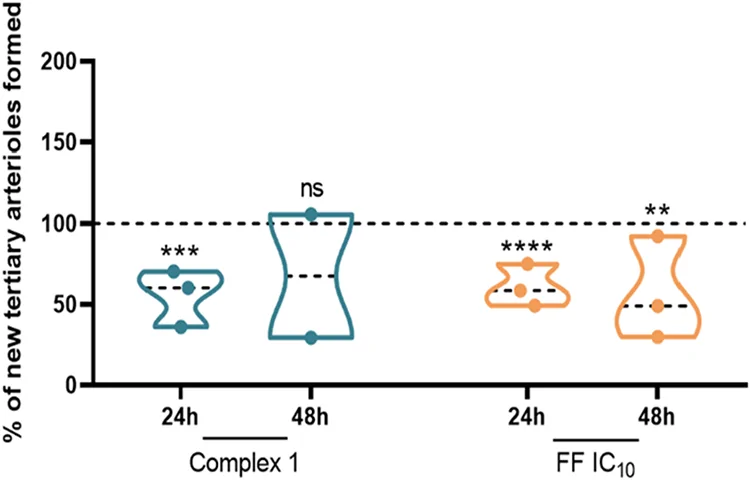
Formation of new tertiary arterioles (%) following chick embryo exposure to complex **1** (3D IC_50_) and FF (3D IC_10_) for 24 and 48 h. DMSO (at the same final concentration as the highest complex concentration) and FF vehicle controls were used as negative controls. Results were normalized to the number of tertiary arterioles observed at 0 h and to the corresponding DMSO-treated condition at each time point for the same embryo. The dashed line represents the DMSO control normalized to vessel counts at 0 h. Data are presented as mean ± SEM from a minimum of five independent biological replicates (embryos). Statistical significance relative to the control was determined by two-way ANOVA (ns, not significant; **p < 0.01; ***p < 0.001; ****p < 0.0001).

As shown in Figure 14, complex **1** exhibited an apparent antiangiogenic effect within the first 24 h, evidenced by an approximately 60% reduction in tertiary arterioles relative to the DMSO control. By 48 h, however, this effect was not maintained, and given that only single data points were available for this condition, these observations should be interpreted with caution. FF exhibited an antiangiogenic activity at both time points. Although the combination of FF and complex **1** was not tested, its results can be speculated. Since FF exhibits antiangiogenic properties throughout the 48 h tested and complex **1** demonstrates the same capacity, even though only at the 24 h time point, it might be expected that the association of both will have a higher antiangiogenic capacity at 24 h than complex **1** and FF alone and a similar effect to FF at 48 h.

Furthermore, it is worth noting that after 48 h of incubation, none of the biological replicates died, a possible indicator that neither the complex nor FF exerted detectable toxic effects *in vivo* in the chicken embryos. These results further enhance the antiproliferative/antitumor potential of complex **1** and its evaluation in more complex biological systems, with the potential for eventual clinical application either as a monotherapy or in combination with existing treatment regimens.

## Conclusions

This work presents the anticancer properties of two new Cu­(II) complexes with 4′-(3,4,5-trimethoxy-phenyl)-2,2′:6′,2″-terpyridine. Cytotoxicity evaluation showed that all complexes displayed lower IC_50_ values than Dox and FF, with complexes **1** and **2** exhibiting high SI values in both tumor cell lines. As SI values were comparable, subsequent studies focused on the clinically relevant HCT116-DoxR model to assess the capability of the complexes to overcome Dox-associated resistance mechanisms. When tested in 3D spheroids, IC_50_ values increased relative to 2D cultures, as expected due to the greater structural complexity of this model. The cytotoxicity of both complexes was further evaluated in tumor- and normal tissue–derived patient-derived organoid (PDOs) models from five different CRC patients. In these models, higher concentrations of both complexes were required to induce damage in tumor-derived PDOs without affecting normal tissue–derived PDOs, compared with the observed in 2D cultures and 3D spheroid models. Complex **1** was evaluated alone and in combination with FF (IC_10_) in 3D spheroids and tumor- and normal tissue-derived PDOs to explore potential combinatorial effects. Notably, although both complexes showed similar cytotoxicity in 2D, complex **1** demonstrated superior activity in 3D spheroids and tumor- and normal tissue-derived PDOs, and it was therefore selected for further biological exploitation. These results underscore the importance of incorporating more complex cellular models, such as 3D spheroids and PDOs, into drug development and validation.

Complex **1** alone or in combination with FF induced higher levels of apoptosis than Cis, with the combination producing an apoptotic response greater than that of complex **1** alone. Complex **1** also caused mitochondrial membrane depolarization, which was further enhanced by FF, suggesting potentiation of cytotoxicity and induction of the intrinsic apoptosis pathway. In addition, complex **1**, alone or in combination with FF, triggered autophagy (nevertheless, the combination strategy did not show a higher increase compared to that of complex **1** alone) and exhibited pronounced cytostatic activity, inducing S-phase arrest at the 9 h time point. In the case of complex **1** alone, both types of cell death are triggered by increased ROS production, while this effect was not observed in combination with FF. Finally, all tested conditions reduced angiogenesis in the CAM assay at 24 h, without detectable toxicity until the 48 h time point.

Overall, these results highlight the therapeutic promise of complex **1**, particularly in combination with the FF regimen in Dox-resistant CRC cells and support its further evaluation in advanced preclinical models. As a take-home message, our findings underscore the importance of incorporating PDOs into drug development and validation workflows. In future studies, inclusion of a large number of PDO pairs will be essential to establish robust correlations between patients’ molecular profiles and therapeutic responses. Such data sets could further enable the application of artificial intelligence-based approaches to predict individual patient responses, ultimately supporting more precise and personalized therapeutic strategies—Precision Oncology.

## Experimental Section

### Materials and Methods

All of the chemicals and solvents used for synthesis and spectroscopic measurements were obtained from Merck. The tmp-terpy (4′-(3,4,5-trimethoxy-phenyl)-2,2′:6′,2″-terpyridine) ligand^30,78–83^ was synthesized according to a modified Kröhnke condensation procedure.^82,83^ In short, 2-acetylpyridine (2.0 mmol, 0.24 g) and 3,4,5-trimethoxybenzaldehyde (1.0 mmol, 0.20 g) were refluxed in ethanol in the presence of aqueous ammonia. Copper­(II) chloride dihydrate and ammonium hexafluorophosphate were used as received. Both biologically evaluated complexes (**1** and **2**) were confirmed to be of high purity (>95%) by elemental analysis, UPLC, and HR-ESI-MS. The PDOs derived from normal and tumor tissues used were obtained from CRC patients during surgery at the Hospital da Luz, Lisboa, Portugal. Informed consents were obtained before any sample or data collection. The study was approved by the Ethics Committee of Hospital da Luz (CES/118/2022/ME; addenda CES/28/2025/IE). Anonymization of personal data was performed in accordance with GDPR. Detailed methodology for the general characterization of obtained Cu­(II) compounds and more information about the biological assays can be found in the Electronic Information File.

### Synthesis of [CuCl_2_(tmp-terpy)] **(1)**

CuCl_2_·2 H_2_O (0.10 g, 0.6 mmol) was dissolved in 3 mL of distilled water. The solution was added to the tmp-terpy (0.24 g, 0.6 mmol) dissolved in a methanol:chloroform mixture (5:1 v/v, 60 mL) and stirred at RT for 6 h. The green solution was filtered and allowed to evaporate at ambient conditions in a hood, producing green microcrystalline precipitate after several days. The X-ray quality crystals were obtained by recrystallization from acetonitrile:methanol (5:1 v/v) solution.

**[CuCl**_**2**_ **(tmp-terpy)] (1)**. **Yield:** 70%. **HR-ESI-MS:** calcd for C_24_H_21_N_3_O_3_ClCu^+^ [M–Cl]^+^ 497.0567 found 497.0569. **El**. **an**. calcd for C_24_H_21_N_3_O_3_Cl_2_Cu·0.25CH_3_CN·0.75H_2_O: C 52.77, H 4.20, N 8.16% found C 52.49; H 3.82; N 8.12%. **IR (cm** ^**–1**^ **, vs** – **very strong, s** – **strong, m** – **medium, w** – **weak)**: 3569 (m), 3525 (m), 3383 (m), 3104 (w), 3004 (m), 2985 (m), 2967 (m), 2944 (m), 2923 (m), 2837 (w), 2823 (w), 1648 (w), 1604 (s), 1589 (m), 1552 (m), 1509 (m), 1473 (s), 1407 (s), 1372 (m), 1318 (m), 1253 (m), 1188 (w), 1157 (w), 1125 (vs), 1097 (m), 1033 (m), 1021 (m), 993 (m), 904 (w), 856 (m), 841 (w), 791 (s), 748 (m), 733 (m), 718 (m), 678 (m), 655 (w), 636 (w), 589 (w), 570 (w), 525 (w), 420 (w). **UV**–**Vis (DMSO 12.5 μM/nm, (ε·10** ^**4**^ **/M** ^**–1**^ **·cm** ^**–1**^ **))**: 713 (0.011), 348 (2.3), 338 (2.4), 291 (2.5).

### Synthesis of {[CuCl­(tmp-terpy)]·PF_6_}_n_ **(2)**

CuCl_2_·2 H_2_O (0.10 g, 0.6 mmol) and NH_4_PF_6_ (0.10 g, 0.6 mmol) were dissolved in distilled water (10 mL) and heated under reflux for 1 h to obtain a clear blue solution. The tmp-terpy (0.24 g, 0.6 mmol) dissolved in methanol:chloroform (5:1 v/v, 60 mL) was then added dropwise, and the resulting mixture was refluxed for an additional 6 h. After that time, the blue-green solution was filtered hot and allowed to cool in a hood at room temperature. After several days, dark green microcrystalline solids were obtained. The X-ray quality crystals were grown by recrystallization from acetonitrile:methanol (5:1 v/v) solution.

**{[CuCl­(tmp**-**terpy)]·PF**_**6**_ **}**_**n**_ **(2)**. **Yield:** 55%. **HR-ESI-MS:** positive: calcd for C_24_H_21_N_3_O_3_ClCu^+^ [M–Cl]^+^ 497.0567 found 497.0585, negative: calcd for PF_6_^–^ 144.9642 found 144.9648. **El**. **an**. calcd for C_24_H_21_N_3_O_3_ClCuPF_6_: C 44.80, H 3.29, N 6.53% found C 44.68; H 3.16; N 6.75%. **IR (cm** ^–**1**^ **):** 3431 (w), 3118 (w), 3020 (w), 2967 (w), 2926 (w), 2830 (w), 1608 (s), 1588 (s), 1571 (m), 1557 (m), 1511 (m), 1476 (s), 1463 (m), 1407 (s), 1378 (m), 1320 (m), 1261 (m), 1241 (m), 1176 (w), 1156 (w), 1129 (vs), 1098 (m), 1025 (m), 1005 (m), 900 (m), 840 (vs), 788 (s), 736 (m), 716 (m), 681 (m), 643 (w), 589 (w), 557 (vs), 420 (w). **UV**–**Vis (DMSO 12.5 μM/nm, (ε·10** ^**4**^ **/M** ^**–1**^ **·cm** ^**–1**^ **)):** 712 (0.012), 348 (2.5), 338 (2.5), 291 (2.6).

## Supplementary Material









## Notes

Crystallographic data of complex **1**: 2494267 (**1**) (CIF)
Crystallographic data of complex **2**: 2494268 (**2**) (CIF)
Experimental details; X-ray crystallographic data; HR-ESI-MS; FT-IR spectra; molar conductivity values; UPLC traces; UV–Vis absorption spectra, cell viability of HCT116, HCT116-DoxR, and fibroblasts (2D cell lines and 3D spheroids) after exposure to different concentrations of the FF regimen, doxorubicin and cisplatin; clinical information on the patients included in the study and their PDOs; viability of the PDOs after exposure to different concentrations of the FF regimen; and percentage of viable, early apoptotic, late apoptotic, and necrotic HCT116-DoxR cells after exposure to complex **1**, FF, and their combinations (PDF)
Molecular formula strings (CSV)

## Abbreviations Used

ΔΨ_m_: mitochondrial membrane potential
2D: two-dimensional
3D: three-dimensional
5-FU: 5-fluorouracil
BID: BH3-interacting domain death agonist protein
CAM: chick chorioallantoic membrane
CAPIRI: capecitabine + irinotecan
CAPOX: capecitabine + oxaliplatin
CCDC: Cambridge Crystallographic Data Centre
Cis: cisplatin
CRC: colorectal cancer
DCF: 2′,7′-dichlorofluorescein
DMSO: dimethyl sulfoxide
Dox: doxorubicin
ECM: extracellular matrix
ESI: electrospray ionization
FA: folinic acid
FBS: fetal bovine serum
FF: FOLFOX regimen
FOLFIRI: folinic acid + 5-fluorouracil + irinotecan
FOLFOX: folinic acid + 5-fluorouracil + oxaliplatin
FT-IR: Fourier transform infrared spectroscopy
H_2_DCFDA: 2′,7′-dichlorodihydrofluorescein diacetate
HCT116: human colorectal carcinoma cell line HCT116
HCT116-DoxR: doxorubicin-resistant HCT116 cell line
HR-MS: high-resolution mass spectrometry
ILCT: intraligand charge transfer
JC-1: 5,5′,6,6′-tetrachloro-1,1′,3,3′-tetraethylbenzimidazolylcarbocyanine iodide
logP: logarithm of partition coefficient
MDR: multidrug resistance
MeCN: acetonitrile
MTS: (3-(4,5-dimethylthiazol-2-yl)-5-(3-carboxymethoxyphenyl)-2-(4-sulfophenyl)-2H-tetrazolium)
OX: oxaliplatin
PBS: phosphate-buffered saline
PDA UV–Vis: photodiode array ultraviolet–visible spectroscopy
PDOs: patient-derived organoids
PgP: P-glycoprotein
Ph-terpy: 4′-phenyl-substituted 2,2′:6′,2″-terpyridine
PI: propidium iodide
PS: phosphatidylserine
ROS: reactive oxygen species
R-terpy: 4′-substituted 2,2′:6′,2″-terpyridines
SEM: standard error of the mean
SI: selectivity index
SQ­(P): square-pyramidal geometry parameter
terpy: 2,2′:6′,2″-terpyridine
TME: tumor microenvironment
tmp-terpy: 4′-(3,4,5-trimethoxyphenyl)-2,2′:6′,2″-terpyridine
UPLC: ultraperformance liquid chromatography
UV–vis: ultraviolet–visible spectroscopy
τ_5_: angular structural index for five-coordinate metal centers
