# Supplementary material for: From 2D to 3D Colorectal Cancer Patient-Derived Organoids (PDOs) and In Vivo Models: Copper(II) Complexes as Promising New Antitumor Agents
Source: J Med Chem. 2026 Jul 31;69(15):19088–106. doi: 10.1021/acs.jmedchem.6c00941 (PMC13492366; doi:10.1021/acs.jmedchem.6c00941)
Supplement: Supplementary file 1 [file jm6c00941_si_003.pdf]

ELECTRONIC SUPPLEMENTARY INFORMATION

From 2D to 3D colorectal cancer patient-derived organoids (PDOs) and *in vivo* models: copper(II) complexes as promising new antitumor agents

Katarzyna Choroba[a,¥,*], Sandra Cordeiro [b,c,¥], Rita Sequeira [b,c,¥], Barbara Machura[a], Karol Erfurt[d], Ana Guedes[e,,f], Luís Mascarenhas-Lemos[g,h,i], Marília Cravo[g,j], Pedro V. Baptista[b,c,*] and Alexandra R. Fernandes [b,c,*]

[a] *Institute of Chemistry, University of Silesia, Szkolna 9, 40-006 Katowice, Poland*

[b] *Associate Laboratory i4HB - Institute for Health and Bioeconomy, NOVA School of Science and Technology, NOVA University Lisbon, 2819-516 Caparica, Portugal*

[c] *UCIBIO, Departamento de Ciências da Vida, NOVA School of Science and Technology, Campus de Caparica, 2829-516 Caparica, Portugal*

[d] *Department of Chemical Organic Technology and Petrochemistry, Silesian University of Technology, Krzywoustego 4, 44-100 Gliwice, Poland*

[e] *Hospital Beatriz Ângelo, Avenida Carlos Teixeira 3, 2674-514 Loures, Portugal*

[f] *Hospital da Luz Learning Health, Luz Saúde, Avenida Lusíada 100, 1500-650 Lisboa, Portugal*

[g] *Hospital da Luz, Avenida Lusíada 100, 1500-650 Lisboa, Portugal*

[h] *NOVA Medical School, Campo Mártires da Pátria 130, 1169-056 Lisboa, Portugal*

[i] *Catolica Medical School, Palma de Cima, 1649-023 Lisboa, Portugal*

[j] *Lisbon School of Medicine, Universidade de Lisboa, Avenida Professor Egas Moniz, 1649-028 Lisboa, Portugal*

*corresponding authors: katarzyna.choroba@us.edu.pl (KC), pmvb@fct.unl.pt (PVB), ma.fernandes@fct.unl.pt (ARF)

[¥] co-first authors

Table of Contents

Experimental Details 4

Table S1. Crystal data and structure refinement of **1** and **2**. 9

Table S2. Selected bond lengths [Å] and angles [°] of **1** and **2**. 11

Table S3. Short intra- and intermolecular hydrogen bonds detected in complexes **1** and **2**. .. 11

Table S4. Short π•••π interactions for complexes **1** and **2**. 12

Table S5. Y—X•••Cg(J)(π-ring) interactions for complexes **1** and **2**. 12

Table S6. X—H•••Cg(J)(π-ring) interactions for complexes **1** and **2**. 12

Table S7. Distortion of the coordination sphere of metal ions from ideal five-vertexes polyhedral (pentagon, vacant octahedron, trigonal bipyramid, spherical square pyramid, and Johnson trigonal bipyramid) calculated using the SHAPE program[5,6] and the angular structural index parameter τ_5[7] for complex **1**. 13

Table S8. Dihedral angles between mean planes of the aromatic rings of 4′-(3,4,5-trimethoxy-phenyl)-2,2′:6′,2″-terpyridine (tmp-terpy) coordinated to the central ion in the complexes **1** and **2**. 13

Figure S1. Intermolecular interactions of the complexes **1** and **2**. 15

Figure S2. HR-ESI-MS spectra of the complexes **1** and **2**. 17

Figure S3. FT-IR spectra of the complexes **1**, **2** and ligand tmp-terpy. 18

Table S9. Molar conductivity values Λ_M (S cm^2 mol^{-1}) of complexes **1** and **2** measured in MeCN and H_2O. (T = 25 °C). Concentration chosen depending on solubility of the compounds: 1 mM (**2** in MeCN), 0.5 mM (**1** and **2** in H_2O) or 0.25 mM (**1** in MeCN) 19

Figure S4. UPLC of the complexes **1** and **2**. Eluent: MeCN:H_2O (80:20 v:v), injection 2 μl, flow 0.30 ml/min, PDA detector. 19

Figure S5. Comparison of UV-Vis spectra of complexes **1** and **2** measured with spectrophotometer in MeCN and the spectra detected during UPLC measurements. 20

Figure S6. UV-Vis spectra of the complexes **1** and **2** and tmp-terpy ligand in DMSO, MeCN (c = 12.5 μM). In DMSO insert, absorption spectra in the 500–1000 nm region were also recorded in concentrated solutions (1 mM). 20

Figure S7. UV-Vis kinetic spectra of complexes **1** and **2** in MeCN, DMSO and PBS/DMSO mixture (90:10 v/v) (c = 12.5 μM). Spectra were measured every 4 h for 48 h. 21

Figure S8. Viability of HCT116, HCT116-DoxR**,** and fibroblasts cell lines after exposure to different concentrations of the FF regimen for 48 h. 22

Figure S9. Viability of HCT116 cancer cell line and fibroblasts after exposure to different concentrations of doxorubicin and cisplatin for 48 hours. 23

Figure S10. Viability of HCT116 and HCT116-DoxR 3D spheroids after exposure to different concentrations of FF regimen for 48 h. 24

Table S10. Clinical information of the patients included in the study and their respective PDOs. 24

Figure S11. Viability of 4-day-old PDO pairs after exposure to different concentrations of the FF regimen for 48 h. 25

Table S11. Percentage of viable, early apoptotic, late apoptotic, and necrotic HCT116-DoxR cells after 48 h exposure to complex **1** (IC_{50} in 2D models), FF (IC_{10} in 2D models), and their respective combinations. 25

Supplementary References 26

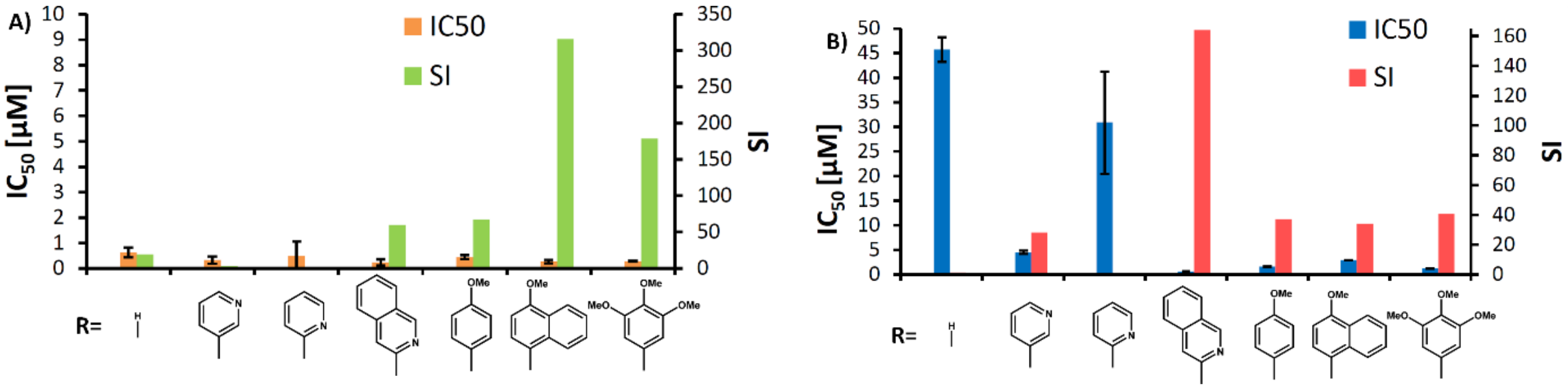

Scheme S1. IC_{50} (μM) and SI values of substituted terpyridine-containing coordination complexes [AuCl(R-terpy)]$(PF_6)_2$ (**A**) and [PtCl(R-terpy)](CF_3SO_3) (**B**) against the HCT116 cell line. Reproduced from ref. [1]. Copyright 2025 American Chemical Society.

Experimental Details

General Characterization

Single-crystal X-ray diffraction data were collected at room temperature on an Oxford Diffraction Gemini A Ultra diffractometer (Oxford Diffraction Ltd., Abingdon, U.K.) equipped with a CCD Atlas detector using graphite-monochromated Mo Kα radiation (λ = 0.71073 Å). Data collection, unit-cell refinement, and data reduction were carried out using the CrysAlisPro software package. Structure solution and refinement were performed with Olex2 (v1.5) employing the SHELXS-2014 and SHELXL-2014[2–4] programs and full-matrix least-squares refinement on F^2. All non-hydrogen atoms were refined anisotropically. Hydrogen atoms were placed in calculated positions and refined using a riding model with fixed isotropic displacement parameters: Uiso(H) = 1.2Ueq(C) (C–H = 0.93 Å) for aromatic hydrogen atoms and Uiso(H) = 1.5Ueq(C) (C–H = 0.96 Å) for methyl hydrogen atoms. Methyl groups were allowed to rotate freely about their local threefold axes. Molecular graphics and crystallographic analyses were performed using Mercury (v2021.2.0), PLATON, and SHAPE 2.0. Crystallographic data have been deposited with the Cambridge Crystallographic Data Centre under deposition numbers 2494267 and 2494268 for **1** and **2**, respectively. Copies of the data can be obtained free of charge from the CCDC via www.ccdc.cam.ac.uk/getstructures.

High-resolution mass spectra were acquired on a Xevo G2 Q-TOF mass spectrometer (Waters Corp., Milford, MA) equipped with an electrospray ionization (ESI) source. Compound 1 was analyzed in positive ion mode, whereas compound 2 was examined in both positive and negative ion modes. Full-scan spectra were collected over the m/z range 100–1000 with a scan time of 0.5 s. Data were acquired in centroid mode and mass accuracy was ensured by external calibration using a LockSpray™ leucine enkephalin reference solution ($[M + H]^+$, m/z 556.2771). Data processing and analysis were performed using MassLynx software (Waters).

Carbon, hydrogen, and nitrogen elemental analyses were carried out using a Vario EL Cube analyzer (Elementar Analysensysteme GmbH, Langenselbold, Germany).

Fourier-transform infrared (FT-IR) spectra were recorded using the KBr pellet technique on a Nicolet iS5 FT-IR spectrophotometer (Thermo Fisher Scientific, Waltham, MA) over the range 4000–400 cm^{-1}.

Electrical conductivity (κ, S cm^{-1}) measurements were performed using a CX-505 multifunction meter (ELMETRON, Zabrze, Poland) equipped with an EC-60 electrode. Freshly prepared solutions in acetonitrile and water were measured at 25.0 ± 0.1 °C and monitored for 48 h to assess temporal stability. Molar conductivity values (ΛM, S cm^2 mol^{-1}) were calculated according to the relation $\Lambda_M = \frac{\kappa}{c} \cdot 10^3$, where c is the molar concentration.

Ultra-performance liquid chromatography (UPLC) measurements were conducted using a Waters Acquity UPLC system (Waters Corp., Milford, MA) equipped with an autosampler and photodiode array (PDA) detector. Separations were achieved on a Waters Acquity UPLC™ BEH C8 column (2.1 × 100 mm, 1.7 µm particle size) maintained at 30 °C. The mobile phase consisted of acetonitrile–water (80:20, v/v) delivered at a flow rate of 0.30 mL min^{-1}. The injection volume was 2 µL, and the total run time was 3 min. Eluted compounds were monitored over the wavelength range 210–400 nm.

UV–visible absorption spectra were recorded on a Nicolet Evolution 220 spectrophotometer (Thermo Fisher Scientific, Waltham, MA) over the range 220–1000 nm. Measurements were carried out at room temperature in acetonitrile, dimethyl sulfoxide, and Dulbecco's Modified Phosphate Buffered Saline (PBS, pH 7.4, 130 mM NaCl). Compound stability was evaluated by recording spectra at 4 h intervals over a 48 h period.

Lipophilicity was evaluated for 1-octanol–PBS mixture (1:1 v:v) at RT through the shake-flask method. PBS and 1-octanol used for the experiments were saturated with each other through vigorous mixing for 24 h. The Cu(II) complexes were used for preparing of a set of dilutions (from 75 µM to 1 µM) in PBS or 1-octanol, and their UV-Vis absorbances were measured to obtain calibration curves. The Cu(II) complexes were dissolved in PBS and added to a 1:1 (v/v) mixture of PBS and 1-octanol so that the final concentration of the compound was 25 µM. The mixtures were shaken on an orbital shaker (Ohaus, Parsippany, NJ) for 12 h, allowed to separate, and the absorbances of organic and aqueous layers were measured. Using calibration curves, the concentrations of the complexes in each of the layers were obtained. The partition coefficient logP was calculated as $\log P = \log(c_{oct}/c_{PBS})$ where the c_{oct} and c_{PBS} are the concentrations of the Cu(II) complex in 1-octanol and PBS layer. Each experiment was repeated three times.

Biological assays

Cell culture and maintenance

HCT116 and neonatal human primary dermal fibroblast cell lines were acquired from the American Type Culture Collection (ATCC®, Manassas, VA, USA). HCT116-DoxR – resistance CRC cell line - was generated from parental HCT116 cells through stepwise exposure to increasing concentrations of Dox, following the protocol established by Pedrosa et al., (2018)[45]. All cell lines were maintained in Dulbecco's Modified Eagle Medium (DMEM) supplemented with 10% (v/v) FBS and 1% (v/v) Penicillin/Streptomycin. To maintain Dox resistance, 3.6 µM Dox was added to the culture medium of HCT116-DoxR cells. All media and supplements were obtained from Thermo Fisher Scientific (USA),

except Dox (Merck, Germany). Cells were grown in T-25 flasks (25 cm²) at 37 °C in a humidified 5% (v/v) CO_2 atmosphere (SANYO CO_2 Incubator, Electric Biomedical Co., Osaka, Japan).

Patient-derived organoids (PDOs) culture and maintenance

Data obtained from each CRC patient were related to tumor location, type of tumor, and TNM classification (**Table S10**). Human tumor and normal tissue samples were harvested into DMEM, maintained on ice, and processed within 4 h after surgery. The tissues were then washed with PBS supplemented with Primocin (0.1 mg/ml) and cut into small fragments. Later, the fragments were enzymatically digested in advanced DMEM/F-12 medium containing 5 mg/ml collagenase II (Sigma-Aldrich, Darmstadt, Germany), 0.25 mg/ml DNase I (Roche/Sigma, Darmstadt, Germany), for 1 h at 37°C under slow rotation. The lysate was filtered through a 70 µm cell strainer, centrifuged at 300 xg for 10 min, and then washed with PBS. Then, the pellet was mixed with Matrigel basement membrane (Corning/Sigma, Darmstadt, Germany) and seeded as drops in a 6-well plate. After 30 min at 37 °C, the Matrigel had polymerized, and CRC complete medium was added to the cells. CRC full medium included: 50% (v/v) advanced DMEM/F12, 50% (v/v) WRN conditioned media (media containing WNT3A, R-spondin and noggin proteins), glutamax (GIBCO, #35050061), 10mM HEPES, 0.1 mg/ml primocin, B27 supplement (GIBCO, #17504044), 1.25 mM n-acetylcysteine, 10 mM nicotinamide, 5 µM A83-01 (Sigma-Aldrich, Darmstadt, Germany), 50 ng/mL EGF, 1 µM prostaglandin E2 (Abcam, Cambridge, UK), and 10 µM y-27632 (MedChemExpress, Sollentuna, Sweden). Tumor- and normal tissue-derived PDOs' medium was changed every 2/3 days, and, when needed, organoids were passaged after dissociation with cold PBS.

Cell viability evaluation in 2D models

To evaluate the effects of the Cu(II) complexes, cell viability was analyzed in HCT116, HCT116-DoxR, and neonatal human primary dermal fibroblasts. Cells were plated in 96-well plates (0.75×10^5 cells/mL) and incubated for 24 h under the conditions described above. Fresh medium containing the Cu(II) complexes or FOLFOX (FF) at concentrations ranging from 0.001 to 100 µM was added to each well. For FF, 5-FU was dissolved in 100% (v/v) DMSO, oxaliplatin (OX) in 5% glucose solution, and folinic acid (FA) in colorless complete medium. Negative controls included DMSO at the same final concentration as in the complexes, while 0.4 µM Dox (for HCT116 and fibroblasts) and 12 µM Dox (for HCT116-DoxR) served as positive controls. Following 48 h of treatment, fresh medium containing MTS reagent (3-(4,5-dimethylthiazol-2-yl)-5-(3-carboxymethoxyphenyl)-2-(4-sulfophenyl)-2H-tetrazolium) was added (CellTiter 96® Aqueous One Solution Cell Proliferation Assay Kit (Promega, Madison, USA)). Cell viability was indirectly measured via absorbance measurement at 490 nm, which correlates with the proportion of viable cells[77–79], using a Tecan Infinite M200 microplate reader (Tecan, Männedorf, Switzerland). IC_{50} values (the concentration of complex that reduces cell viability by 50%) for each complex and FF in the different cell lines were determined based on the dose-response curves obtained from GraphPad Prism 8.0.1 Software. The SI was determined by dividing the relative IC_{50}'s of fibroblasts by the value obtained in each CRC cell line.

3D-spheroid and PDOs formation and cell viability assays

HCT116 and HCT116-DoxR cells at a density of 0.5×10^5 cells/mL were seeded in ultra-low adherence 96 well-plates (NunclonTM SpheraTM Microplate, Thermo Fisher Scientific, Waltham, MA, USA) and incubated at the conditions mentioned above for 6 days to allow spheroid formation and growth. For normal- and tumor-PDOs formation, 10 μL matrigel droplets containing 1×10^4 PDOs/mL were plated in 96-well plates and incubated under the same conditions for 4 days. Following these incubation periods, the PDOs and spheroids were treated with target concentrations of each complex or FF, and incubated for an additional 48 h. Subsequently, 50 μL of medium from each well was replaced with 50 μL of CellTiter-Glo® 3D reagent (CellTiter-Glo® 3D Luminescent Cell Viability Assay Kit, Promega, Madison, USA). Spheroid plates were incubated for 15 min with agitation, followed by 45 min without agitation, while PDOs plates were incubated for 5 min with agitation and an additional 25 min. Subsequently, 90 μL from each well was carefully transferred to a solid-bottom, opaque 96-well plate, and luminescence was recorded using a Tecan Spark® multimode microplate reader (Tecan, Männedorf, Switzerland). The resulting values were used to calculate cell viability, and the relative IC_{50} values for the complexes/FF in spheroids and organoids/tumoroids were obtained using GraphPad Prism 8.0.1 (GraphPad Software Inc., San Diego, USA).

Evaluation of apoptosis induction

Apoptosis induction in HCT116-DoxR cells was evaluated by flow cytometry using the Alexa Fluor 488 Annexin V Apoptosis Detection Kit (Thermo Fisher Scientific, Waltham, MA, USA). Cells' treatments included the 2D IC_{50} of the selected Cu(II) complex, the 2D IC_{10} of FF, or a combination of both. DMSO (at the same final percentage as in treatment solutions) and FF vehicles served as negative controls, while 6 μM Dox and 5 μM Cis were used as positive controls. After 48 h, TrypLE™ Express was used to detach the cells, which were later washed with PBS, and resuspended in Annexin V binding buffer. Following 15 min incubation at room temperature (RT) with Annexin V–Alexa Fluor 488 and 1 mg/mL propidium iodide (PI), apoptosis induction was quantified by flow cytometry using an Attune® Acoustic Focusing Flow Cytometer (Thermo Fisher Scientific, Waltham, MA, USA).

Mitochondrial membrane potential (ΔΨm) evaluation

The JC-1 Mitochondrial Membrane Potential Assay Kit (Abnova Corporation, Walnut, CA, USA) was used to assess mitochondrial membrane potential (ΔΨm) in HCT116-DoxR cells. 6-well plates were used to seed the cells at 1×10^5 cells/mL, and then they were incubated for 24 h. Cells' treatments involved the substitution of the medium to a fresh one containing the 2D IC_{50} of the Cu(II) complex, the 2D IC_{10} of FF, or a combination of both. DMSO (at the same final percentage as in the treatment solution) and FF vehicles served as negative controls, while 6 μM Dox and 5 μM Cis were included as positive controls. After a subsequent 48 h incubation, detachment of the cells was effected with TrypLE™ Express, then they were washed with PBS, and later incubated with JC-1 solution prepared in colorless medium containing 5% (v/v) FBS for 20 min at 37 °C. Subsequently, cells were resuspended

in fresh phenol red–free medium with 5% (v/v) FBS, and mitochondrial membrane potential was measured using an Attune® Acoustic Focusing Flow Cytometer (Thermo Fisher Scientific, Waltham, MA, USA).

Autophagy induction

The Autophagy Assay Kit (ab139484) (Abcam, Cambridge, UK) was utilized to evaluate the complex's capacity to induce cell death by autophagy in HCT116-DoxR cells. Cells were seeded in 6-well plates at a density of $1x10^5$ cells/mL and incubated for 24 h. Cells' treatments involved their exposure to the 2D IC_{50} of Cu(II) complex, the 2D IC_{10} of FF, or a combination of both. DMSO (at the same final percentage used in treatment solution) and FF vehicles were employed as negative controls, while 6 μM Dox and 5 μM Cis were used as positive controls. After a total treatment time of 48 h, rapamycin (1.5 μM) was added for the final 18 h, as an additional positive control for autophagy induction. Subsequently, the detachment of the cells with TrypLE™ Express was executed, then the cells were washed with colorless medium supplemented with 5% (v/v) FBS and incubated for 30 min with the Green Stain solution prepared in the same medium. After staining, the pellet was resuspended in assay buffer, and autophagic activity was quantified by flow cytometry using an Attune® Acoustic Focusing Flow Cytometer (Thermo Fisher Scientific, Waltham, MA, USA).

Evaluation of ROS production

HCT116-DoxR cells were plated in 6-well plates at a density of $1x10^5$ cells/mL and incubated for 24 h. After exposure to medium supplemented with the 2D IC_{50} of the Cu(II) complex, the 2D IC_{10} of FF, or a combination of both. DMSO (at the same final concentration used in the complex solution) and FF vehicles were considered negative controls, while 6 μM Dox, 5 μM Cis, and 50 μM H_2O_2 were used as positive controls. Next to 48 h of treatment, cells were detached with TrypLE™ Express, washed with PBS, and incubated with 10 μM 2′,7′-dichlorodihydrofluorescein diacetate (H_2DCF-DA; Thermo Fisher Scientific, Waltham, MA, USA) in PBS for 20 min. Reactive oxygen species (ROS) production was then assessed using an Attune® Acoustic Focusing Flow Cytometer (Thermo Fisher Scientific, Waltham, MA, USA).

Cell cycle progression

HCT116-DoxR cells were seeded in 6-well plates at a density of $1x10^5$ cells/mL and incubated for 8 h at 37 °C in a humidified atmosphere with 5% (v/v) CO_2. Cell cycle synchronization at the early S-phase was achieved by treating cells with 2 mM thymidine (Merck, Germany) for 16 hours. The thymidine-containing medium was replaced with fresh medium, and after 8 hours, a second thymidine block was applied under identical conditions. Following the second 16 h incubation, cells were exposed to the 2D IC_{50} of the selected complex, the 2D IC_{10} of FF, or their combination. DMSO (at the same final concentration used in treatment solution) and FF vehicles were included as negative controls, while 6 μM Dox served as a positive control. Cells were collected at 9, 18, and 24 h post-treatment. At each time point, cells were detached using TrypLE™ Express, centrifuged at 650 xg for 5 min at 4 °C, washed with cold PBS, and centrifuged again at 3000 xg for 5 min at 4°C. Pellets

were resuspended in cold PBS and centrifuged again at 3000 xg for 5 min at 4°C. Pellets were then resuspended in cold PBS and fixed by slow addition of 80% (v/v) ethanol, followed by storage at 4 °C for at least 16 h. After fixation, pellets were centrifuged (7500 xg for 10 min at 4°C) and treated with RNase (50 µg/mL). Following an incubation at 37°C for 30 min, they were stained with 25 µg/mL PI. DNA content was analyzed using an Attune® Acoustic Focusing Flow Cytometer (Thermo Fisher Scientific, Waltham, MA, USA).

Ex-ovo Chick Chorioallantoic Membrane (CAM) assay

To evaluate the selected complex's potential to modulate angiogenesis, the *ex-ovo* CAM assay was performed. Fertilized chicken eggs were incubated at 37 °C under humidified conditions for 72 h. Following embryo stabilization, sterile O-rings were placed on the CAM surface, and PBS solutions containing the 3D IC_{50} of the complex, the 3D IC_{10} of FF, or 0.1% DMSO as a vehicle control were added to them. Images were acquired after 0, 24 and 48 h of incubation at 37 °C using a digital USB microscope camera (Opti-Tekscope OT-V1). Newly formed vessels were manually quantified using ImageJ software. All procedures were conducted in accordance with Directive 2010/63/EU of the European Parliament on the protection of animals used for scientific purposes.

Statistical analysis

Data are expressed as mean ± SEM out of a minimum of two to three independent biological replicates, with technical duplicates in each one unless stated otherwise. GraphPad Prism 8.0.1 (GraphPad Software Inc., San Diego, CA, USA) was used for data analysis, using either one-way ANOVA or Student's t-test, as appropriate, with a statistical significance of $p < 0.05$.

Table S1. Crystal data and structure refinement of **1** and **2**.

Complex	1	2
**Empirical formula**	$C_{24}H_{21}Cl_2CuN_3O_3$	$C_{24}H_{21}ClCuF_6N_3O_3P$
**Formula weight**	533.88	643.40
**Temperature [K]**	293(2)	293(2)
**Wavelength [Å]**	0.71073	0.71073
**Crystal system**	Monoclinic	Monoclinic
**Space group**	C2/*c*	$P2_1$/n
**Unit cell dimensions [Å,°]**	a = 14.4387(10)	a = 14.2726(6)
	b = 22.4308(11)	b = 8.1331(5)
	c = 14.7823(8)	c = 22.6477(11)
	β = 107.806(7)	β = 100.807(4)
**Volume [Å^3]**	4558.2(5)	2582.3(2)
**Z**	8	4
**Density (calculated) [Mg/m^3]**	1.556	1.655
**Absorption coefficient [mm-1]**	1.225	1.088
**F(000)**	2184	1300
**Crystal size [mm]**	0.38 × 0.06 × 0.05	0.61 × 0.26 × 0.05
**θ range for data collection [°]**	3.40 to 25.05	3.57 to 25.05

**Index ranges**	$-15 \le h \le 17$<br>$-26 \le k \le 22$<br>$-17 \le l \le 15$	$-17 \le h \le 14$<br>$-9 \le k \le 9$<br>$-26 \le l \le 26$
**Reflections collected**	9876	12294
**Independent reflections**	4025 (R_{int} = 0.0353)	4561(R_{int} = 0.0450)
**Completeness to 2θ**	99.7%	99.7%
**Min. and max. transm.**	0.279 and 1.000	0.338 and 1.000
**Data / restraints / parameters**	4025 / 0 / 301	4561 / 0 / 355
**Goodness-of-fit on F^2**	1.037	1.059
**Final R indices [I>2σ(I)]**	R_1 = 0.0430<br>wR_2 = 0.1164	R_1 = 0.0543<br>wR_2 = 0.1372
**R indices (all data)**	R_1 = 0.0564<br>wR_2 = 0.1257	R_1 = 0.0698<br>wR_2 = 0.1460
**Largest diff. peak and hole [e $Å^{-3}$]**	0.66 and -0.50	0.55 and -0.86
**CCDC number**	2494267	2494268

Table S2. Selected bond lengths [Å] and angles [°] of **1** and **2**.

**1**		**2**	
**Bond lengths**			
**Cu(1)–N(1)**	2.042(2)	**Cu(1)–N(1)**	2.031(3)
**Cu(1)–N(2)**	1.959(2)	**Cu(1)–N(2)**	1.940(2)
**Cu(1)–N(3)**	2.019(2)	**Cu(1)–N(3)**	2.017(3)
**Cu(1)–Cl(1)**	2.3752(8)	**Cu(1)–Cl(1)^{eq}**	2.2212(9)
**Cu(1)–Cl(2)**	2.2960(8)	**Cu(1)–Cl(1)^{ax}**	2.857(1)
		**Cu(1)–O(2)**	2.657(3)
**Bond angles**			
**N(1)–Cu(1)–N(2)**	78.56(8)	**N(1)–Cu(1)–N(2)**	79.60(10)
**N(1)–Cu(1)–N(3)**	157.40(9)	**N(1)–Cu(1)–N(3)**	159.09(11)
**N(2)–Cu(1)–N(3)**	78.88(9)	**N(2)–Cu(1)–N(3)**	80.48(11)
**N(1)–Cu(1)–Cl(1)**	94.25(7)	**N(1)–Cu(1)–Cl(1)^{eq}**	100.09(8)
**N(2)–Cu(1)–Cl(1)**	115.22(7)	**N(2)–Cu(1)–Cl(1)^{eq}**	178.37(8)
**N(3)–Cu(1)–Cl(1)**	96.57(7)	**N(3)–Cu(1)–Cl(1)^{eq}**	99.64(8)
**N(1)–Cu(1)–Cl(2)**	98.28(7)	**N(1)–Cu(1)–Cl(1)^{ax}**	96.31(8)
**N(2)–Cu(1)–Cl(2)**	138.29(7)	**N(2)–Cu(1)–Cl(1)^{ax}**	90.30(8)
**N(3)–Cu(1)–Cl(2)**	97.58(7)	**N(3)–Cu(1)–Cl(1)^{ax}**	90.05(8)
**Cl(1)–Cu(1)–Cl(2)**	106.48(3)	**Cl(1)^{eq}–Cu(1)–Cl(1)^{ax}**	91.33(3)
		**Cl(1)^{eq}–Cu(1)–O(2)**	98.46(6)
		**Cl(1)^{ax}–Cu(1)–O(2)**	169.86(6)
		**N(1)–Cu(1)–O(2)**	84.6(1)
		**N(2)–Cu(1)–O(2)**	85.7(1)
		**N(3)–Cu(1)–O(2)**	79.91(9)

Cl(1)^{eq} – the Cl(1) atom in the equatiorial position of the coordianation sphere, Cl(1)^{ax} – the Cl(1) atom in the axial position of the coordianation sphere

Table S3. Short intra- and intermolecular hydrogen bonds detected in complexes **1** and **2**.

**D–H•••A**	**D–H [Å]**	**H•••A [Å]**	**D–A [Å]**	**D–H•••A [°]**
**1**				
C(2)–H(2)•••O(3)[a]	0.93	2.55	3.441(4)	159.00
C(4)–H(4)•••Cl(2)[b]	0.93	2.78	3.545(3)	140.00
C(7)–H(7)•••Cl(2)[b]	0.93	2.79	3.591(3)	145.00
C(12)–H(12)•••Cl(1)[c]	0.93	2.72	3.585(3)	156.00
C(24)–H(24C)•••Cl(1)[c]	0.96	2.71	3.527(4)	143.00
**2**				
C(2)–H(2)•••F(5)[d]	0.93	2.49	3.276(6)	143.00
C(4)–H(4)•••F(5)[e]	0.93	2.49	3.281(5)	144.00
C(9)–H(9)•••F(1)[f]	0.93	2.53	3.428(4)	162.00
C(12)–H(12)•••F(1)[f]	0.93	2.52	3.450(4)	175.00
C(14)–H(14)•••F(6)	0.93	2.48	3.201(6)	134.00
C(15)–H(15)•••Cl(1)	0.93	2.83	3.388(4)	120.00

symmetry codes: (a) = x,y,-1+z; (b) = 1/2-x,3/2-y,1-z; (c) = 1-x,y,3/2-z; (d) = 1-x,1-y,1-z; (e) = 1/2+x,3/2-y,1/2+z; (f) = -x,1-y,1-z

Table S4. Short π•••π interactions for complexes **1** and **2**.

Cg(I)•••Cg(J)	Cg(I)•••Cg(J) [Å]	α [°]	β [°]	γ [°]	Cg(I)-Perp [Å]	Cg(J)-Perp [Å]
**1**						
Cg(1)•••Cg(2)[b]	3.4884(16)	4.30(14)	15.18	18.43	-3.3094(12)	-3.3667(11)
Cg(1)•••Cg(3)[c]	3.5908(18)	9.12(15)	25.29	17.68	3.4212(12)	3.2466(14)
Cg(2)•••Cg(2)[c]	3.4334(16)	0	9.19	9.19	3.3894(11)	3.3893(11)
Cg(4)•••Cg(4)[c]	3.672(2)	1	19.50	19.50	3.4610(15)	3.4610(15)
**2**						
Cg(2)•••Cg(3)[g]	3.6707(19)	16.09(15)	6.6	20.5	3.4376(13)	3.6464(13)

symmetry codes: (b) = 1/2-x,3/2-y,1-z; (c) = 1-x,y,3/2-z; (g) = 1/2-x,-1/2+y,3/2-z
α = dihedral angle between *Cg*(I) and *Cg*(J); *Cg*(I)-Perp = Perpendicular distance of *Cg*(I) on ring J; *Cg*(J)-Perp = perpendicular distance of *Cg*(J) on ring I; β = angle *Cg*(I)→*Cg*(J) vector and normal to ring I; γ = angle *Cg*(I) →*Cg*(J) vector and normal to plane J. Cg(1) is the centroid of atoms = N1/C1/C2/C3/C4/C5; Cg(2) is the centroid of atoms = N2/C6/C7/C8/C9/C10; Cg(3) is the centroid of atoms = C16/C17/C18/C19/C20/C21; Cg(4) is the centroid of atoms = N3/C11/C12/C13/C14/C15

Table S5. Y—X•••Cg(J)(π-ring) interactions for complexes **1** and **2**.

Y—X•••Cg(J)	X(I)•••Cg(J) [Å]	X-Perp [Å]	γ [°]	Y(I)—X•••Cg(J) [°]
**1**				
no interactions detected				
**2**				
P(1)—F(2)•••Cg(4)[h]	3.563(4)	3.351	19.82	139.22(19)

symmetry codes: (h) = x,-1+y,z. γ = angle X(I)→Cg(J) vector and normal to plane J. Cg(4) is the centroid of atoms = N3/C11/C12/C13/C14/C15;

Table S6. X—H•••Cg(J)(π-ring) interactions for complexes **1** and **2**.

X—H•••Cg(J)	X•••Cg(J) [Å]	H–Perp [Å]	γ [°]	X(I)—H•••Cg(J) [°]
**1**				
C(24)—H(24A)•••Cg(3)[i]	3.704(5)	-2.80	16.18	140
**2**				
no interactions detected				

symmetry codes: (i) = 1/2-x,3/2-y,2-z. γ = angle X(I)→Cg(J) vector and normal to plane J. Cg(3) is the centroid of atoms = C16/C17/C18/C19/C20/C21.

Table S7. Distortion of the coordination sphere of metal ions from ideal five-vertexes polyhedral (pentagon, vacant octahedron, trigonal bipyramid, spherical square pyramid, and Johnson trigonal bipyramid) calculated using the SHAPE program[5,6] and the angular structural index parameter τ_5[7] for complex **1**.

**Compound**	**SHAPE v2.0 Continuous Shape Measures**					**τ_5**
	**Pentagon PP-5 (D5h)**	**Vacant octahedron vOC-5 (C4v)**	**Trigonal bipyramid TBPY-5 (D3h)**	**Spherical square pyramid SPY-5 (C4v)**	**Johnson trigonal bipyramid J12 JTBPY-5 (D3h)**	
**1**	31.688	5.288	**2.938**	**3.034**	7.313	0.3185

Parameter τ_5[7] is calculated with use of the eq. $\tau_5 = \frac{\beta - \alpha}{60°}$, where α and β are the two greatest valence angles, α < β.

Table S8. Dihedral angles between mean planes of the aromatic rings of 4′-(3,4,5-trimethoxy-phenyl)-2,2′:6′,2″-terpyridine (tmp-terpy) coordinated to the central ion in the complexes **1** and **2**.

**Dihedral angle**	**1**	**2**
**pyridine**$_{central(N2)}$–**pyridine**$_{distal(N1)}$	4.29°	11.43°
**pyridine**$_{central(N2)}$–**pyridine**$_{distal(N3)}$	2.77°	13.31°
**pyridine**$_{distal(N1)}$–**pyridine**$_{distal(N3)}$	6.04°	7.00°
**pyridine**$_{central(N2)}$–Ph	6.17°	46.58°
**terpy**$_{plane}$–**Ph**	4.80°	48.45°

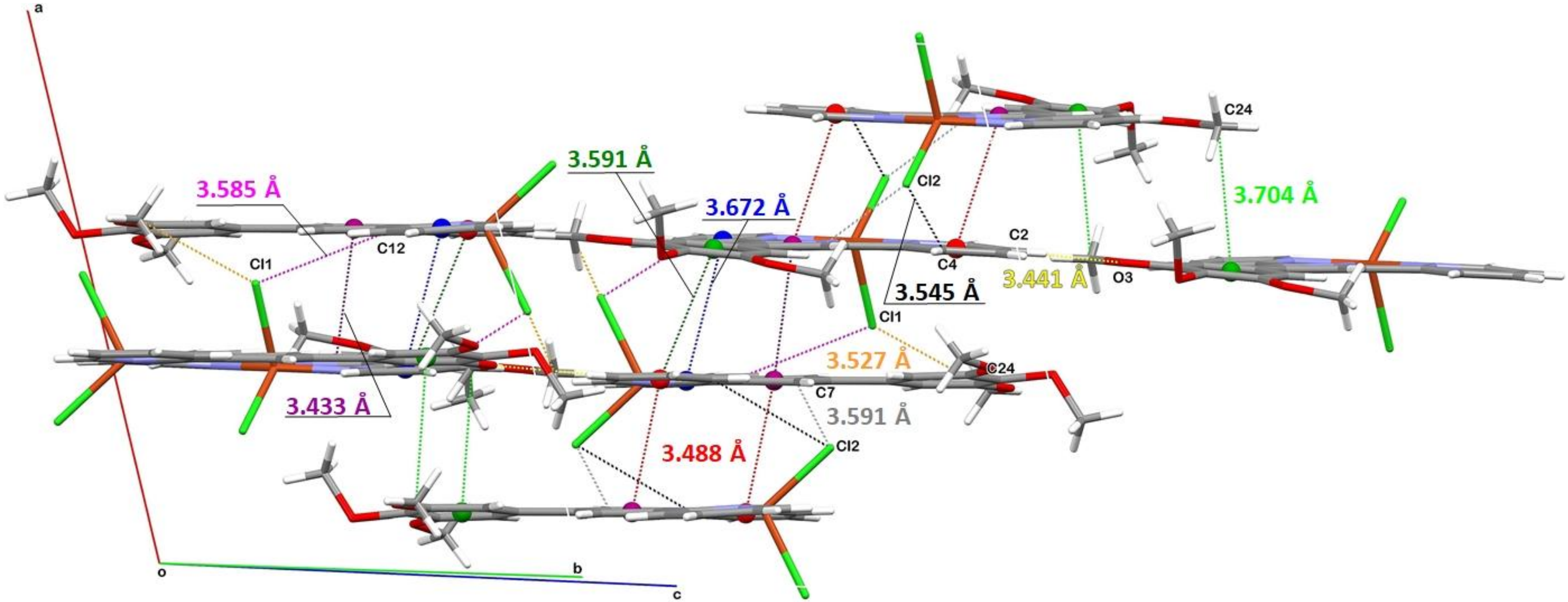
a
3.591 Å
3.585 Å
3.672 Å
C24
Cl2
3.704 Å
C2
Cl1
C12
C4
3.545 Å
3.441 Å
O3
Cl1
3.527 Å
C24
C7
3.433 Å
3.591 Å
Cl2
3.488 Å
o
b
c

1

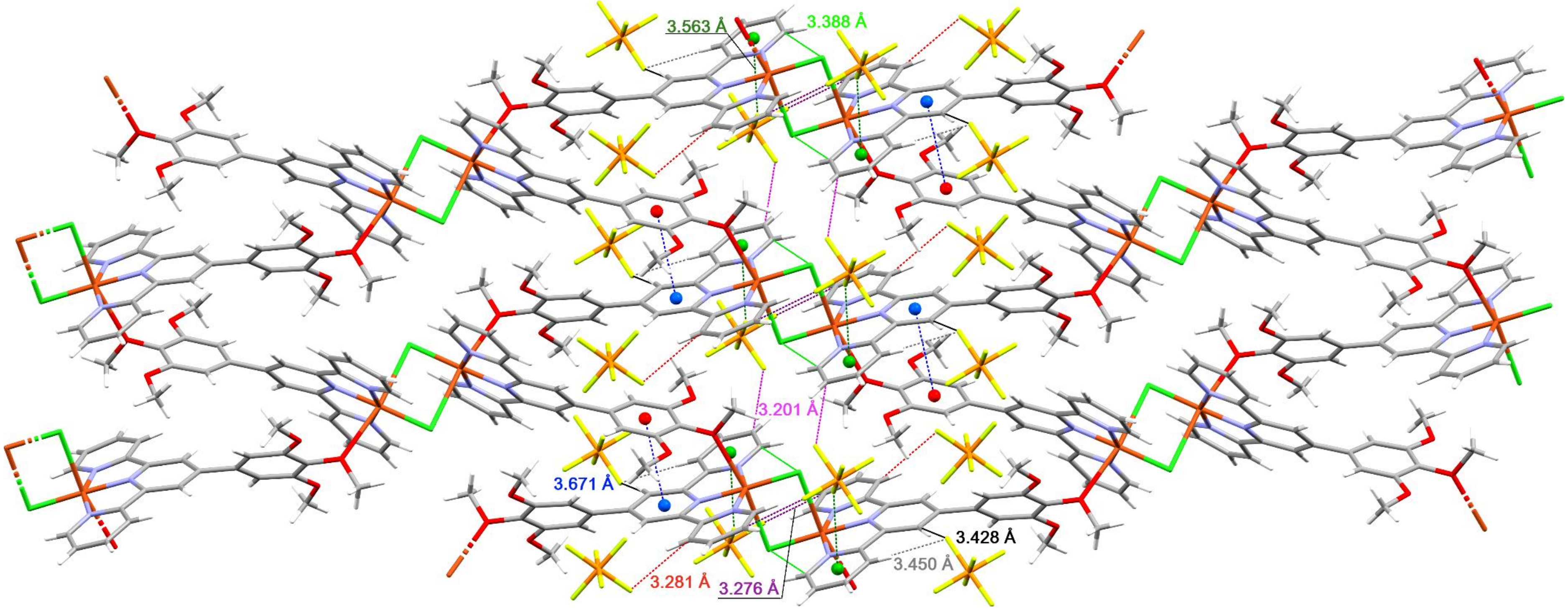

2

Figure S1. Intermolecular interactions of the complexes **1** and **2**.

Multiple Mass Analysis: 2 mass(es) processed
Tolerance = 50.0 PPM / DBE: min = -1.5, max = 50.0
Element prediction: Off
Number of isotope peaks used for i-FIT = 3
Monoisotopic Mass, Even Electron Ions
18 formula(e) evaluated with 4 results within limits (up to 2 best isotopic matches for each mass)
Elements Used:

Mass	RA	Calc. Mass	mDa	PPM	DBE	Formula	i-FIT	i-FIT Norm	Fit Conf %	C	H	N	O	Cl	Cu
497.0569	100.00	497.0567	0.2	0.4	15.5	C24 H21 N3 O3 Cl Cu	478.9	0.000	100.00	24	21	3	3	1	1
		497.0334	23.5	47.3	11.5	C21 H22 N3 O3 Cl2 Cu	489.7	10.832	0.00	21	22	3	3	2	1
499.0547	79.44	499.0491	5.6	11.2	10.5	C21 H24 N3 O3 Cl2 Cu	446.0	0.534	58.61	21	24	3	3	2	1
		499.0724	-17.7	-35.5	14.5	C24 H23 N3 O3 Cl Cu	446.4	0.882	41.39	24	23	3	3	1	1

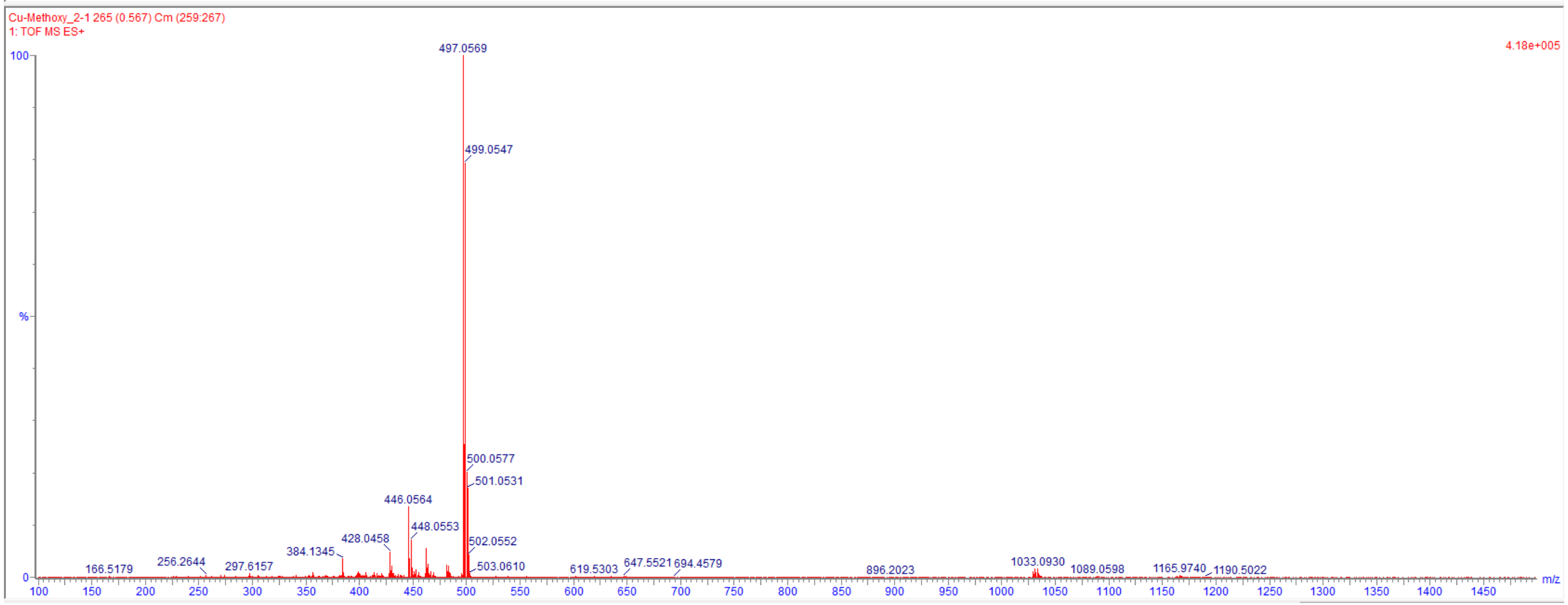

1

HRMS (ESI): calcd for $C_{24}H_{21}N_3O_3ClCu^+$ 497.0567 found 497.0569

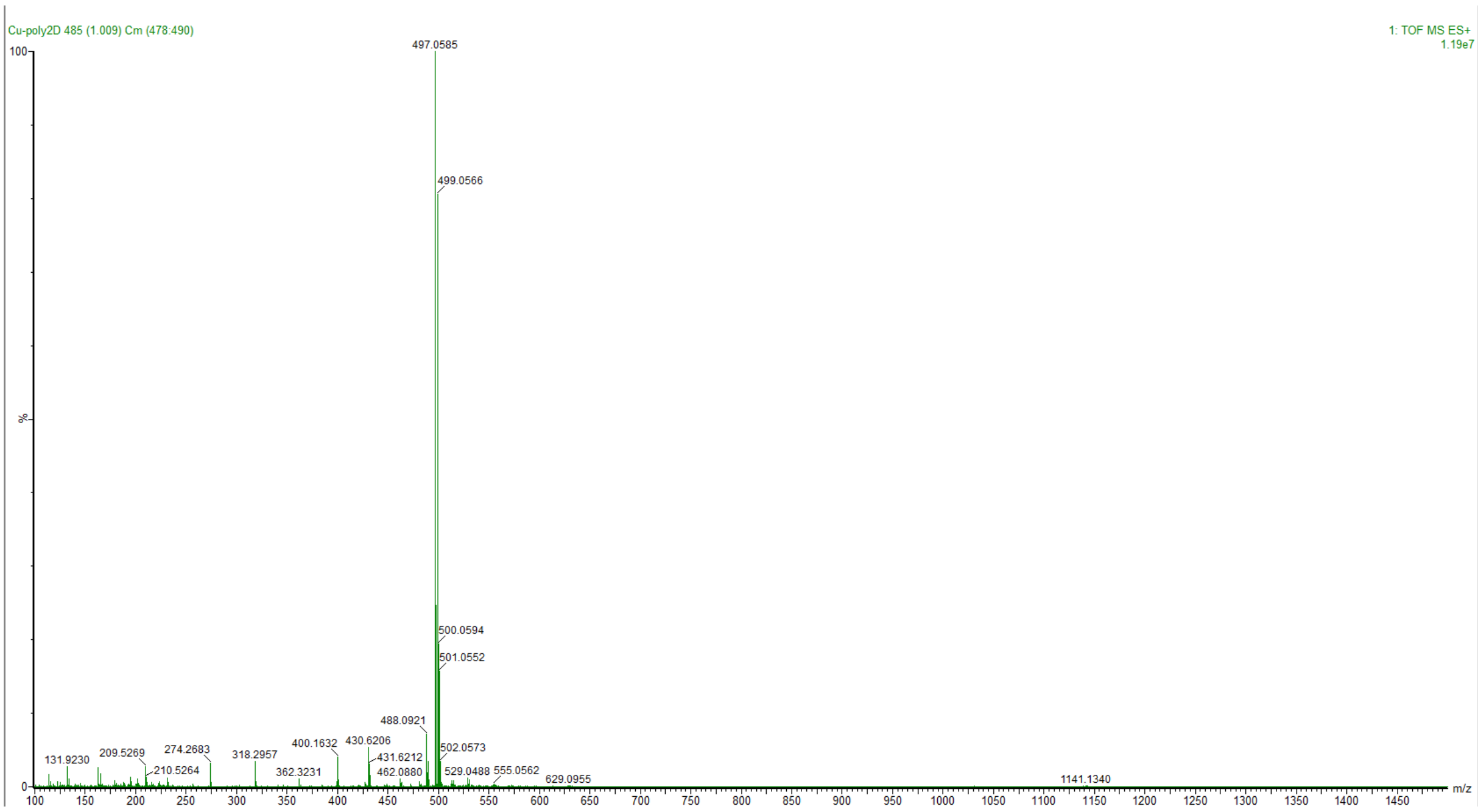

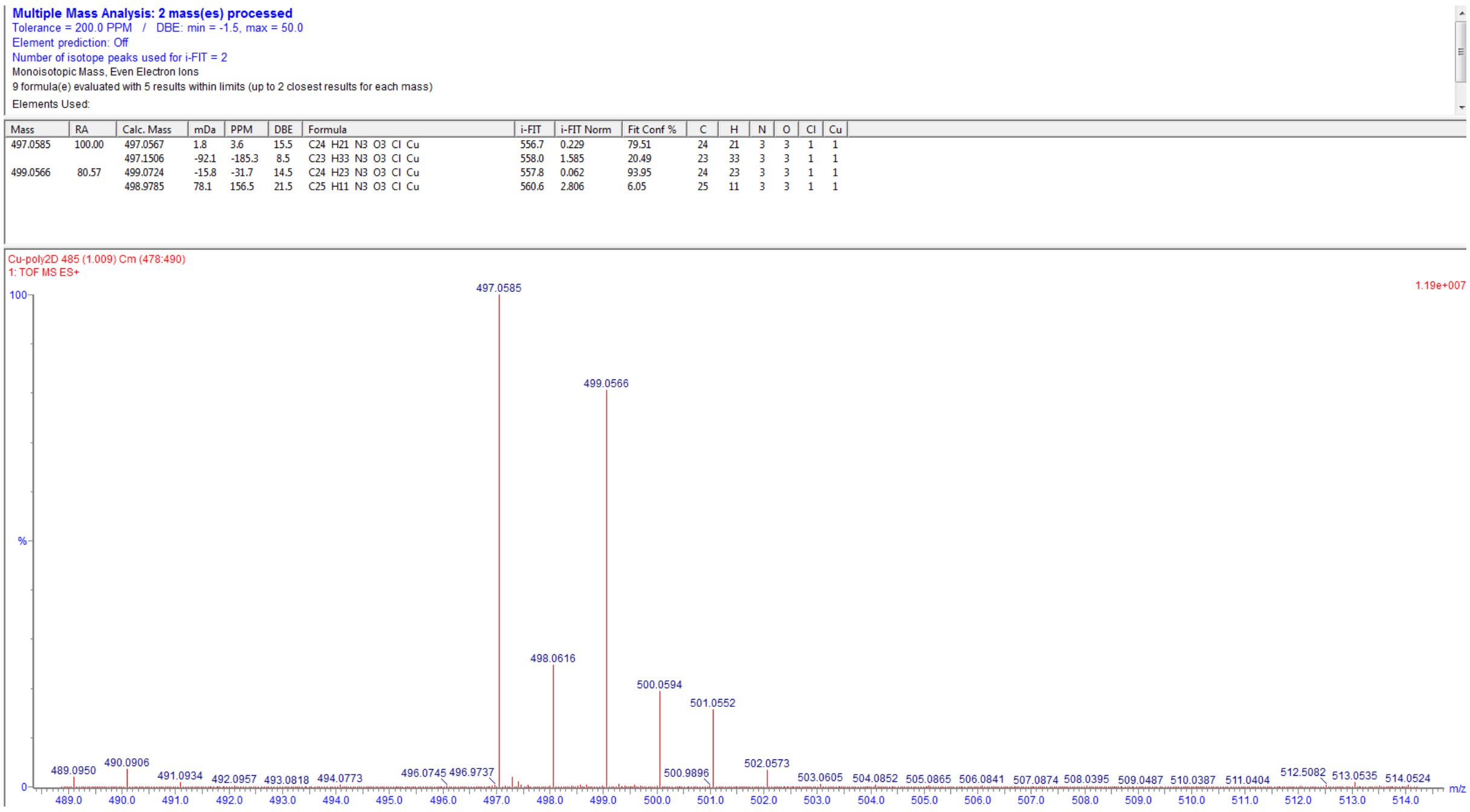

2 (*positive mode*)

HRMS (ESI): calcd for $C_{24}H_{21}N_3O_3ClCu^+$ 497.0567 found 497.0585

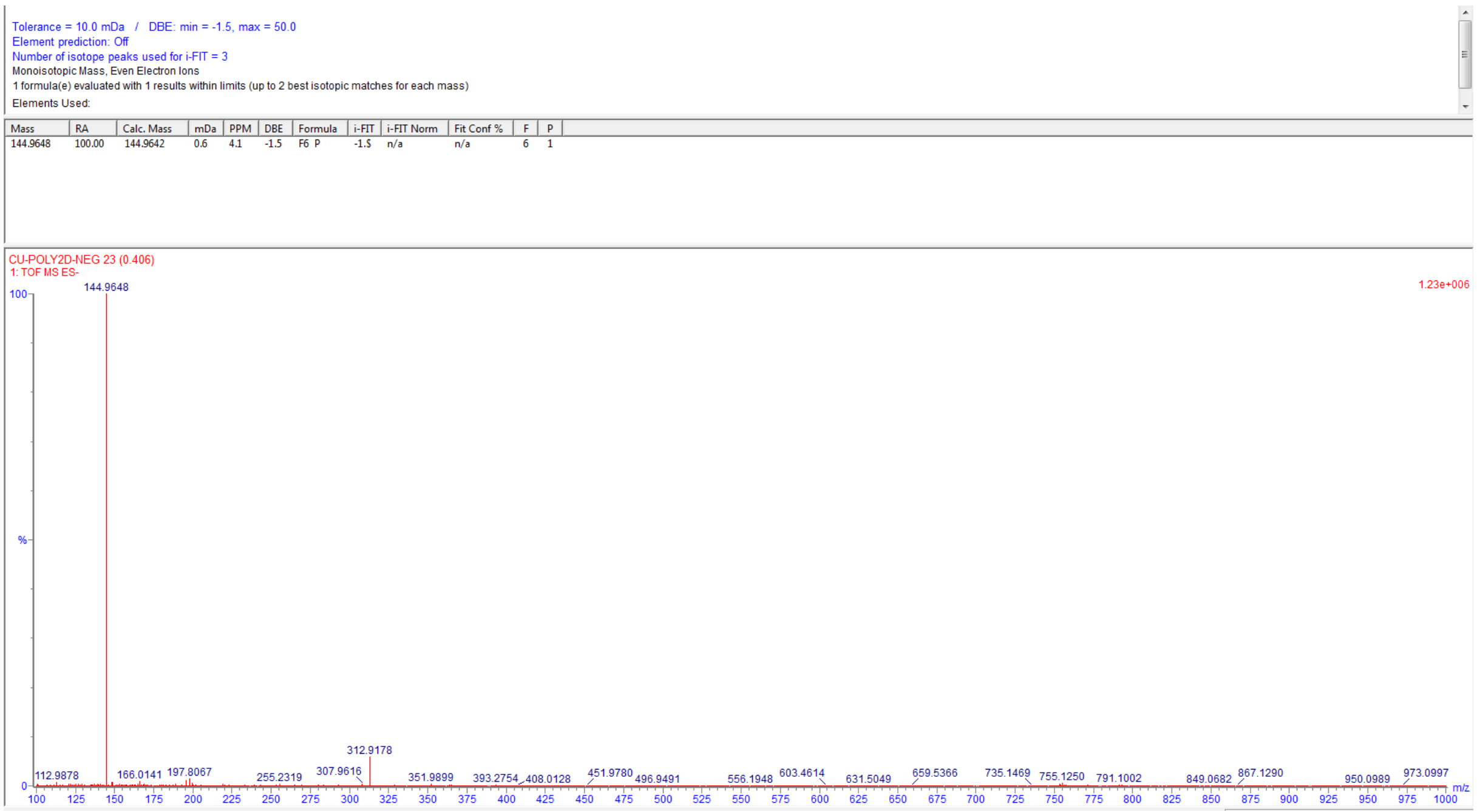

2 (*negative mode*)

HRMS (ESI): calcd for PF_6^- 144.9642 found 144.9648

Figure S2. HR-ESI-MS spectra of the complexes **1** and **2**.

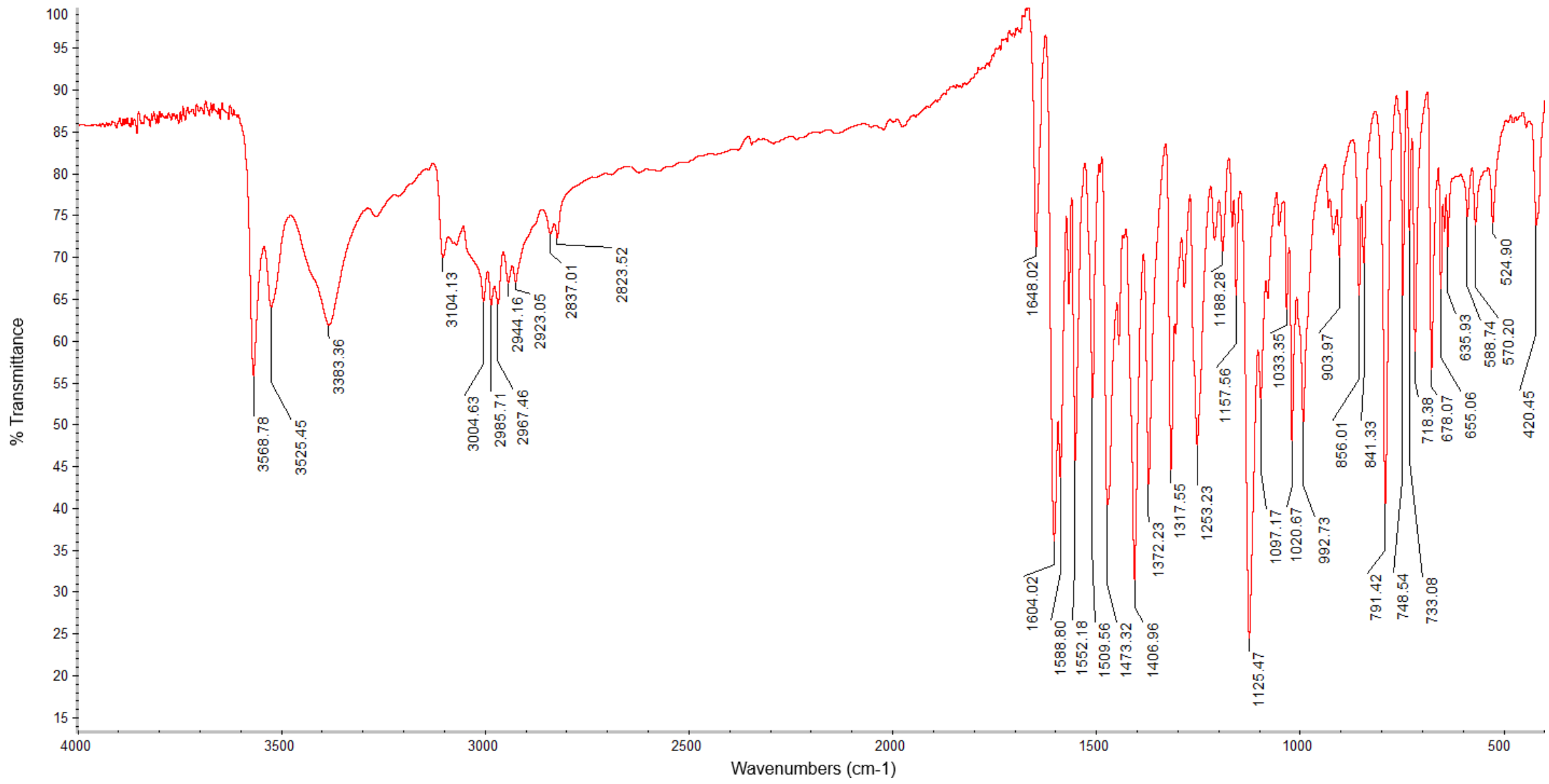

1

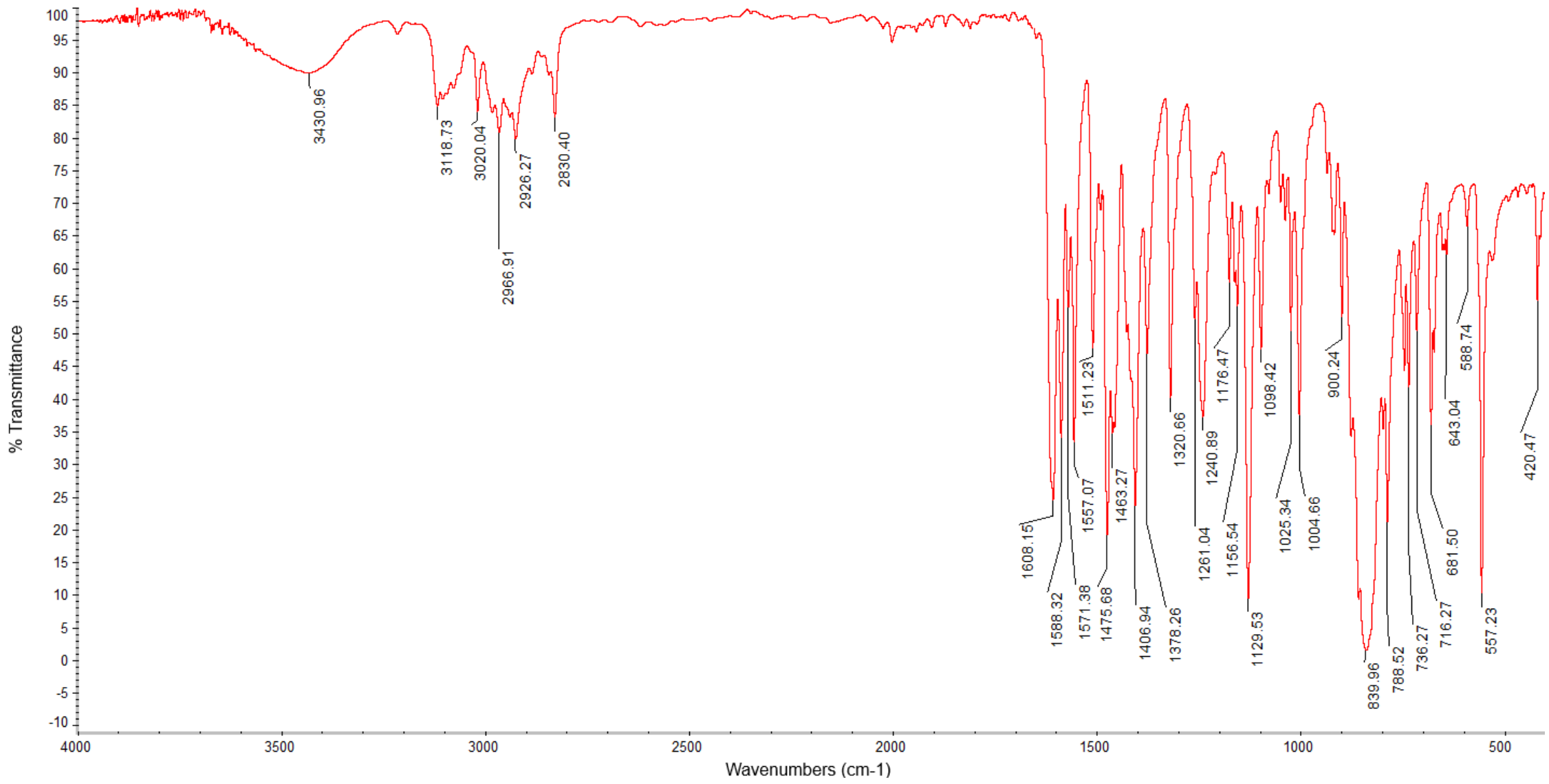

2

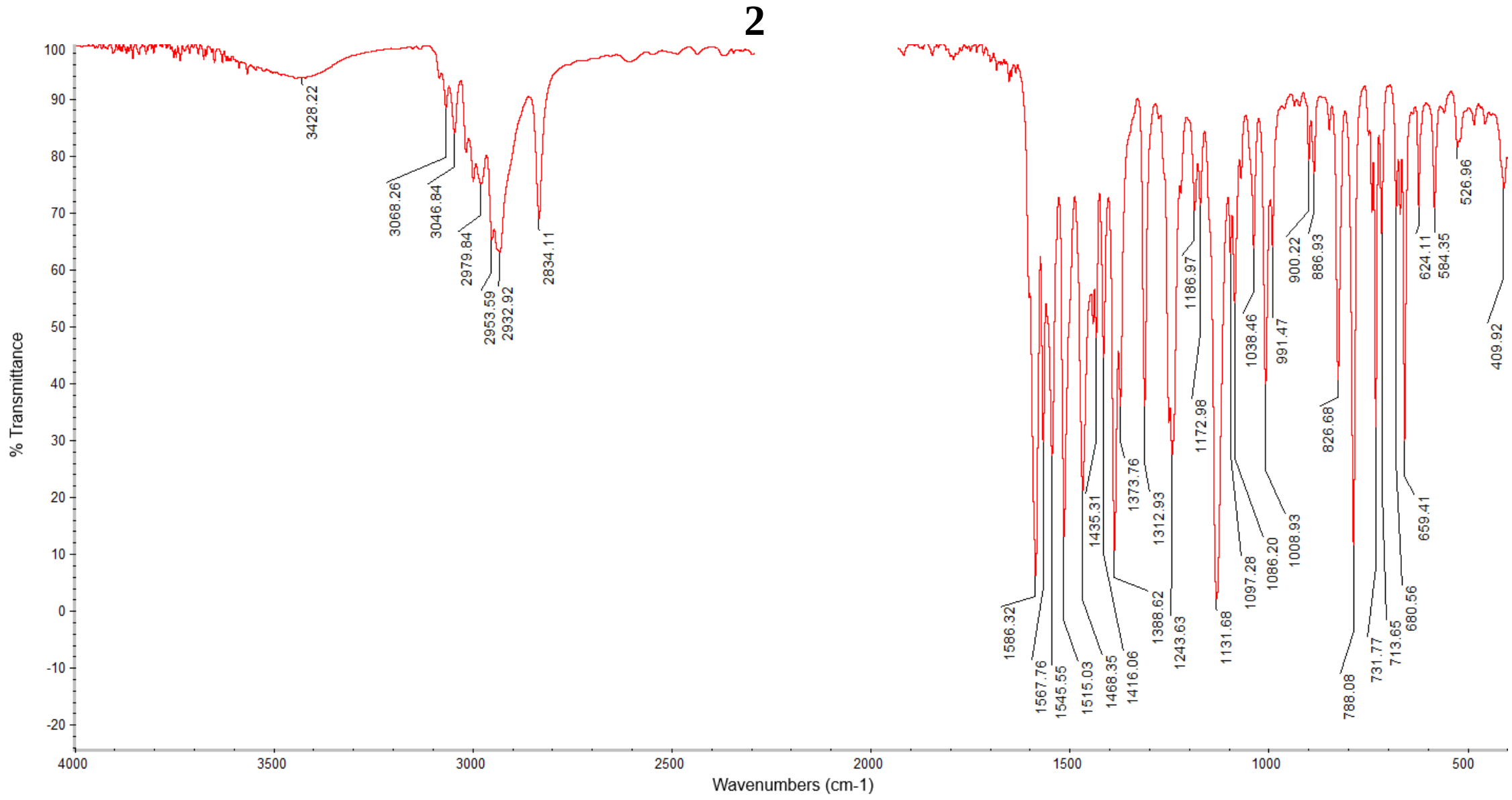

tmp-terpy

Figure S3. FT-IR spectra of the complexes **1**, **2** and ligand tmp-terpy.

Table S9. Molar conductivity values Λ_M (S cm^2 mol^{-1}) of complexes **1** and **2** measured in MeCN and H_2O. (T = 25 °C). Concentration chosen depending on solubility of the compounds: 1 mM (**2** in MeCN), 0.5 mM (**1** and **2** in H_2O) or 0.25 mM (**1** in MeCN)

	**Λ_M (S cm^2 mol^{-1})**					
**Complex**	**1**			**2**		
**time**	**0h**	**24h**	**48h**	**0h**	**24h**	**48h**
**MeCN**	168.5	160.4	168.3	125.9	126.7	132.9
**H_2O**	147.2	147.2	148.1	107.9	106.1	107.6

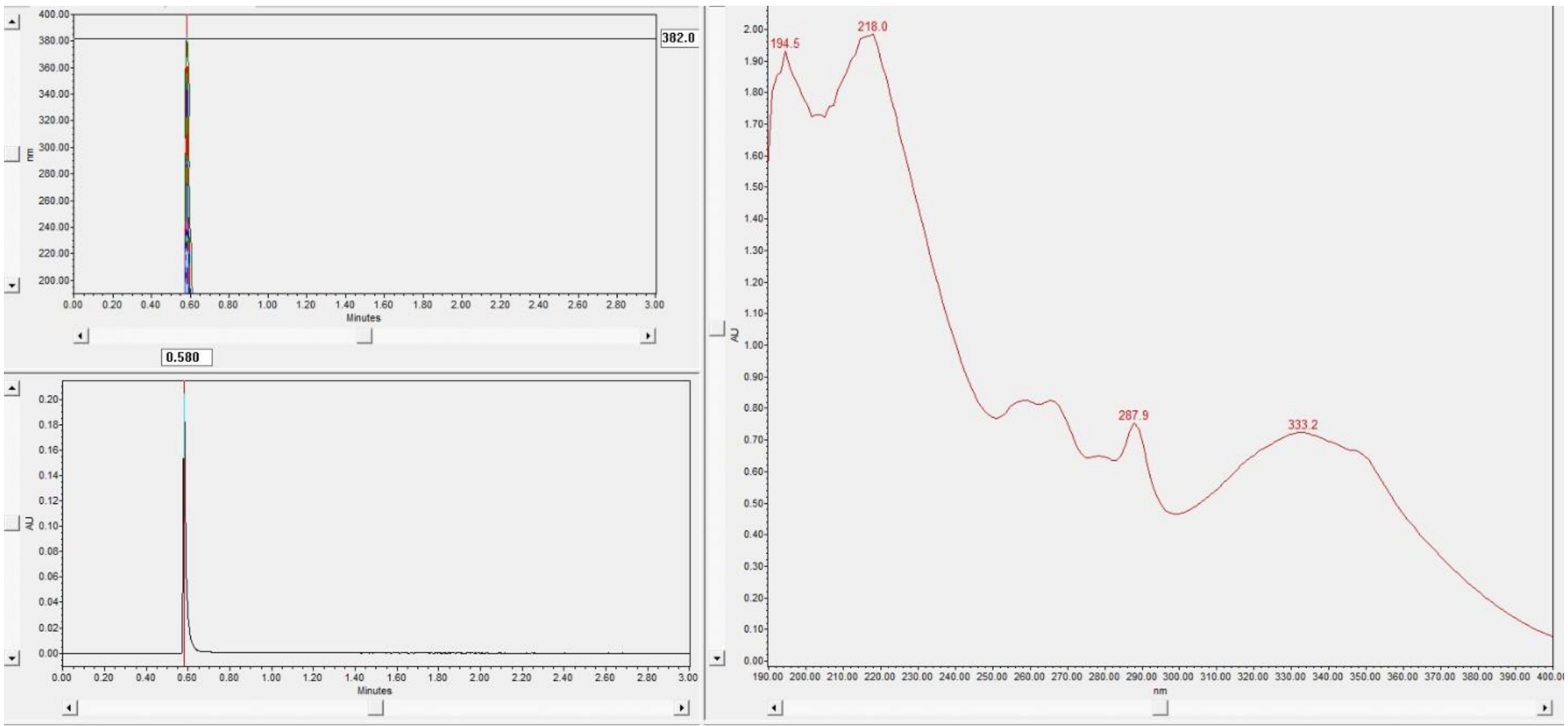

1

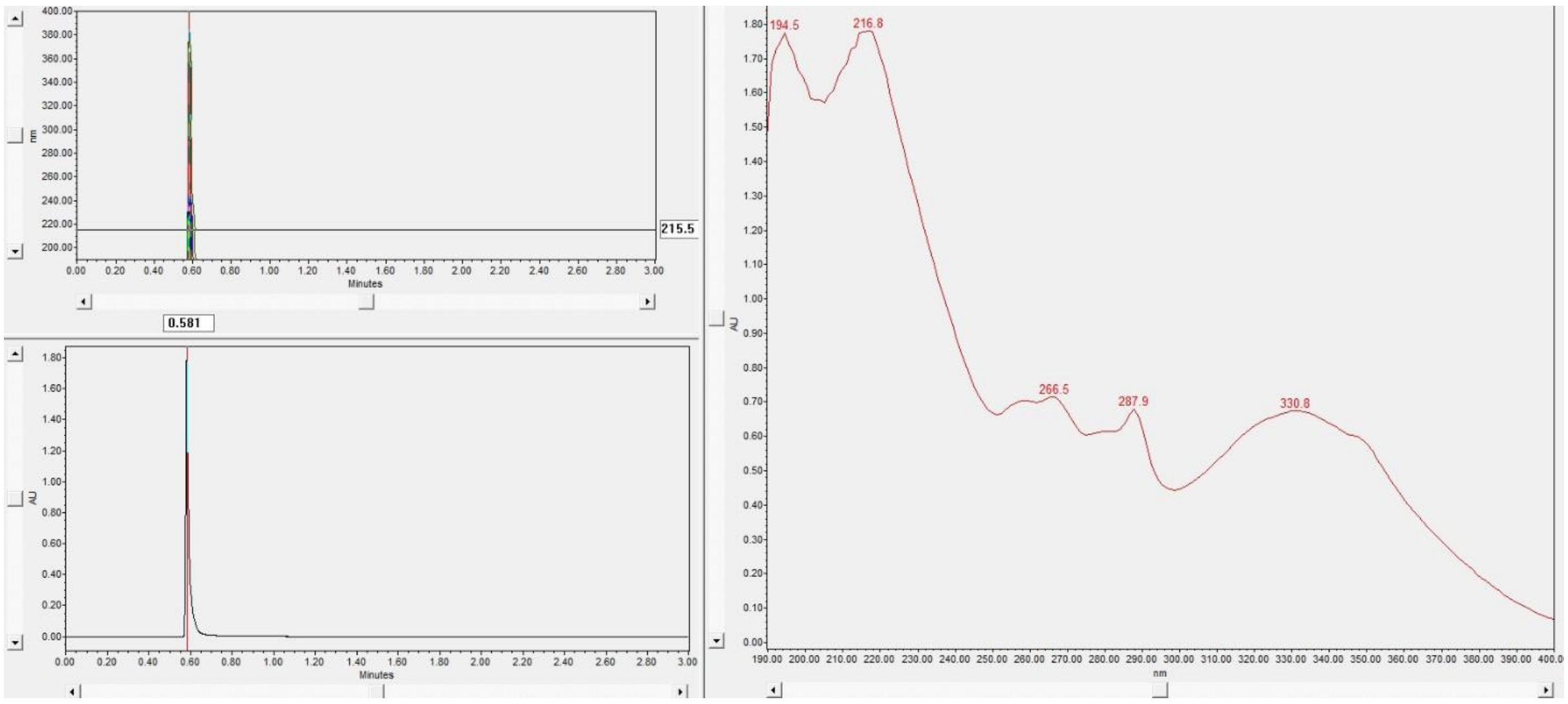

2

Figure S4. UPLC of the complexes **1** and **2**. Eluent: MeCN:H_2O (80:20 v:v), injection 2 µl, flow 0.30 ml/min, PDA detector.

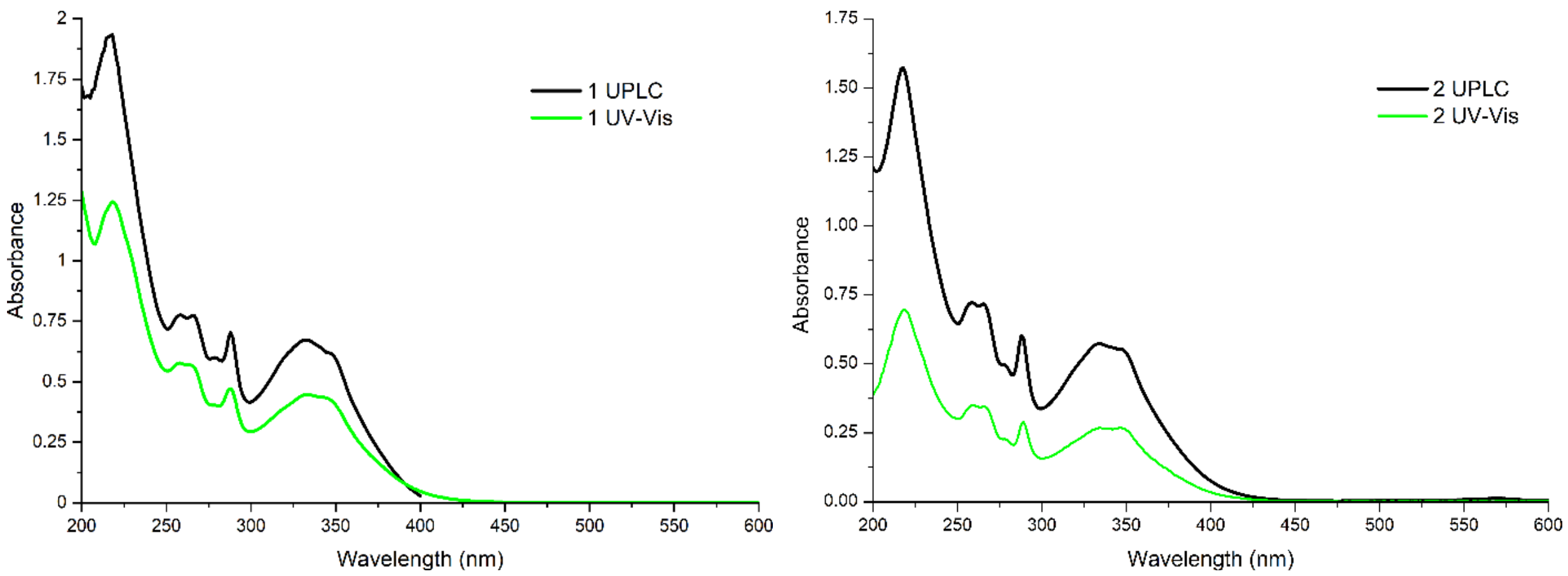

1 **2**

Figure S5. Comparison of UV-Vis spectra of complexes **1** and **2** measured with spectrophotometer in MeCN and the spectra detected during UPLC measurements.

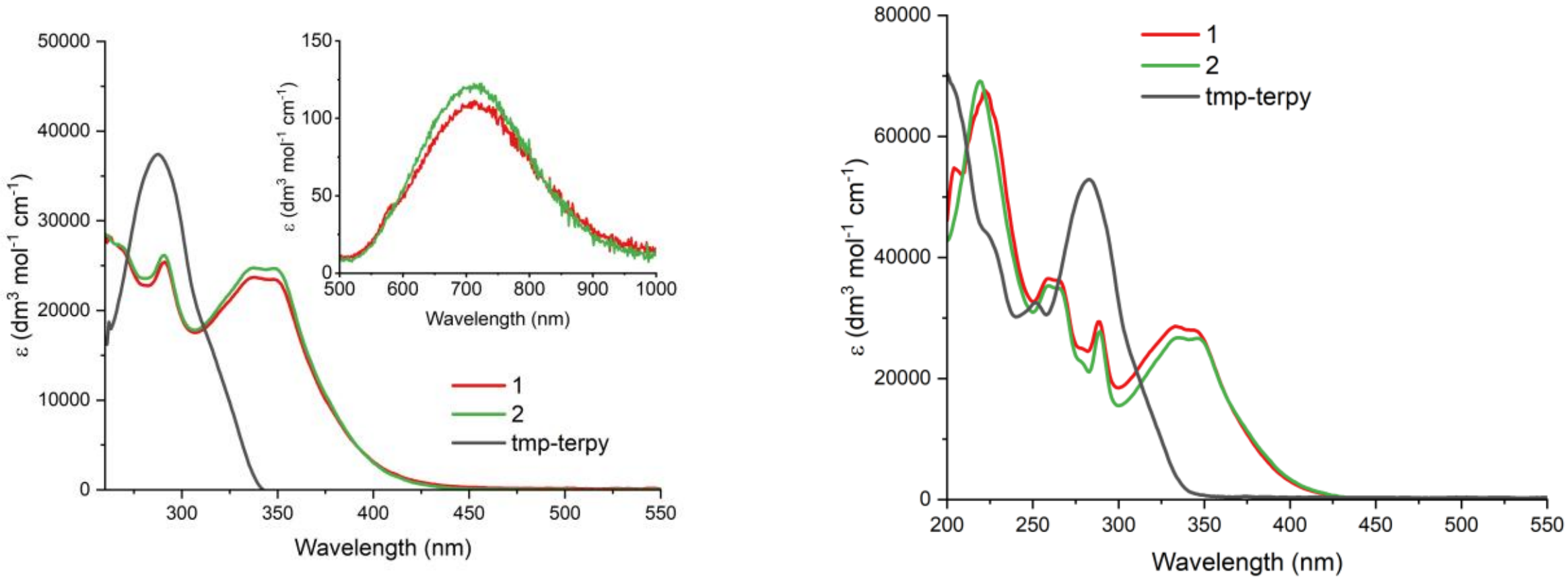

DMSO **MeCN**

Figure S6. UV-Vis spectra of the complexes **1** and **2** and tmp-terpy ligand in DMSO, MeCN (c = 12.5 μM). In DMSO insert, absorption spectra in the 500–1000 nm region were also recorded in concentrated solutions (1 mM).

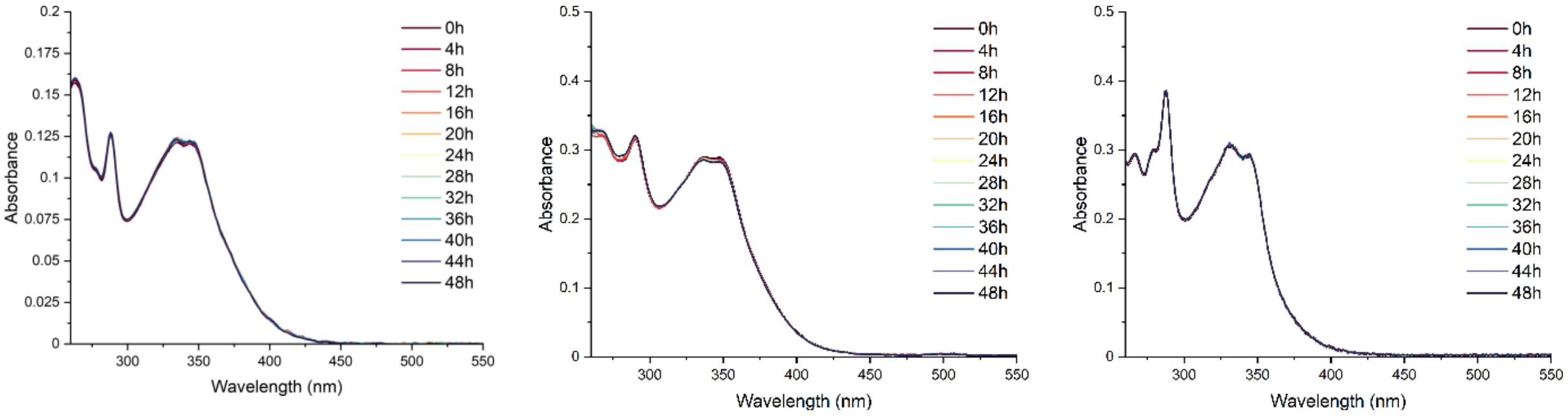

MeCN DMSO PBS

1

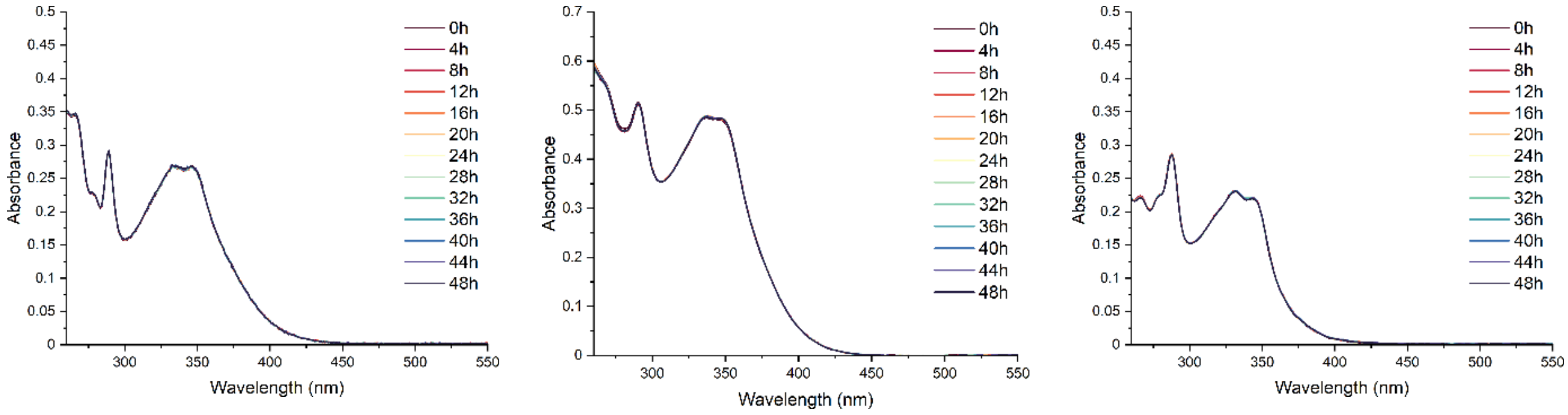

MeCN DMSO PBS

2

Figure S7. UV-Vis kinetic spectra of complexes **1** and **2** in MeCN, DMSO and PBS/DMSO mixture (90:10 v/v) (c = 12.5 μM). Spectra were measured every 4 h for 48 h.

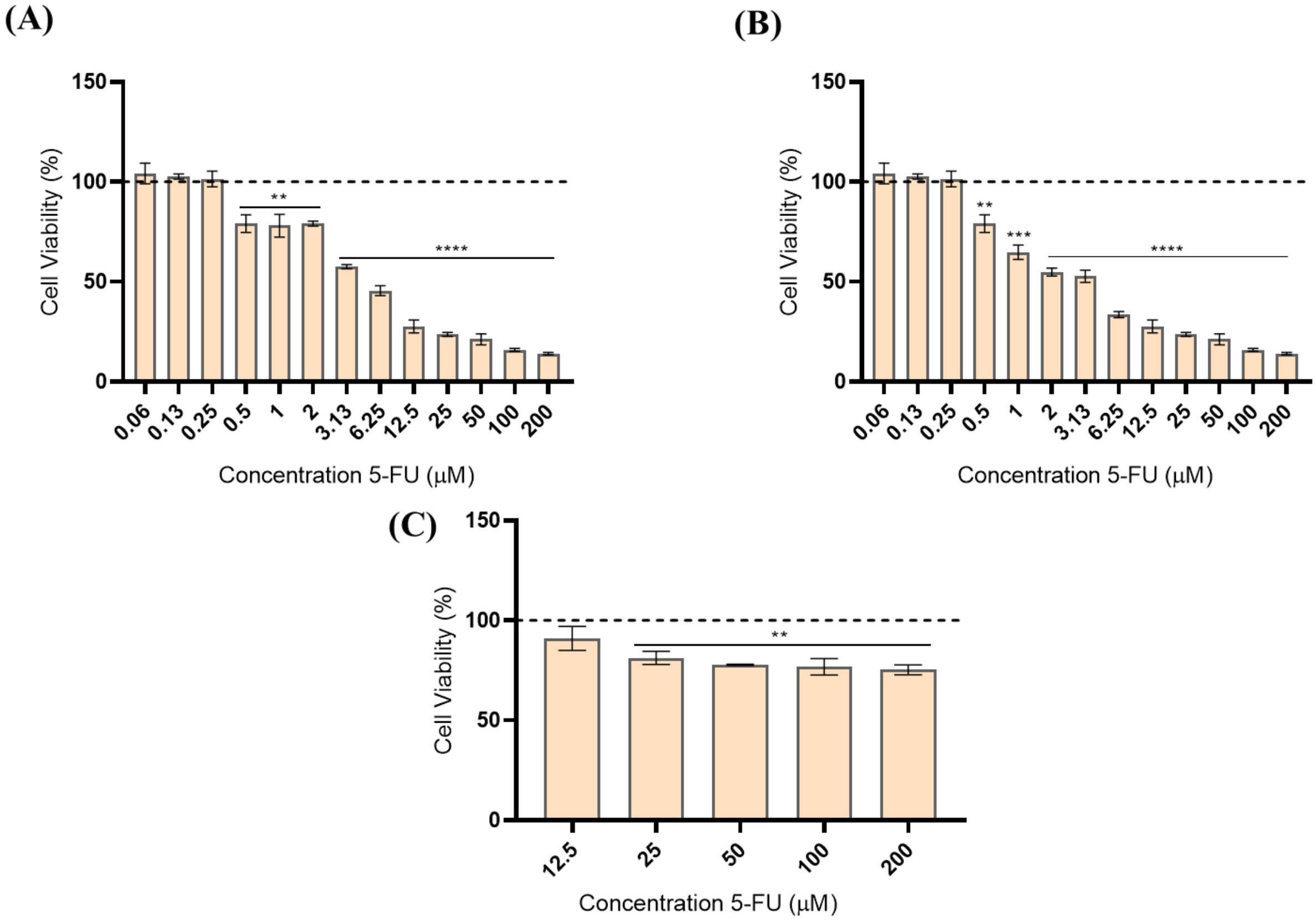

Figure S8. Viability of HCT116 **(A)**, HCT116-DoxR **(B),** and fibroblasts **(C)** cell lines after exposure to different concentrations of the FF regimen for 48 h. The FF regimen was prepared with the ratios 1 5-FU: 0.042 FA: 0.012 OX, and the concentrations expressed in the graphic are based on the 5-FU concentration. The dashed line represents the DMSO control at the same final concentration used for the complexes. Data are presented as mean ± SEM from at least two independent biological replicates. Statistical significance relative to the DMSO control was determined using one-way ANOVA (**$p < 0.01$; ***$p < 0.001$; ****$p < 0.0001$).

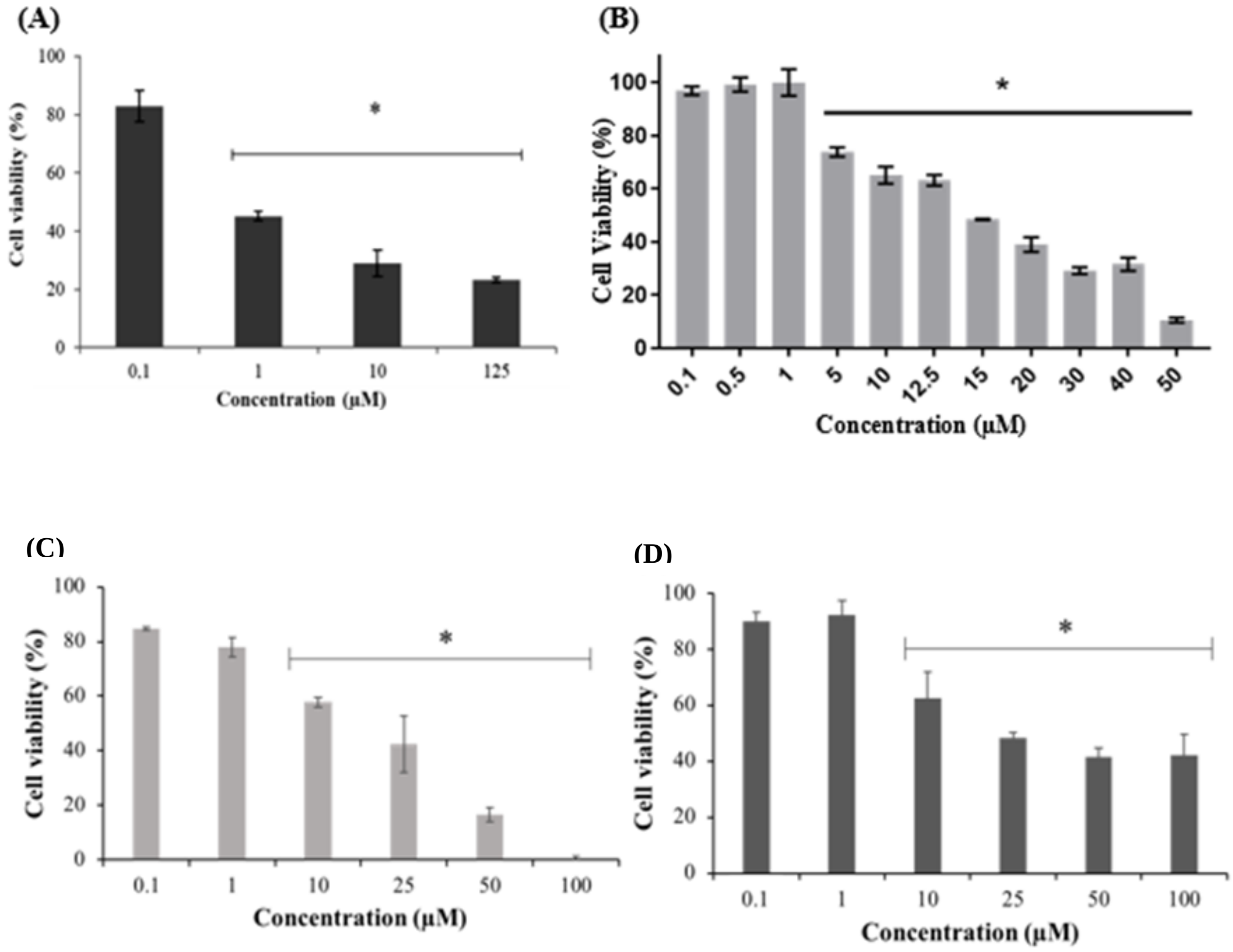

Figure S9. Viability of HCT116 cancer cell line after exposure to different concentrations of doxorubicin **(A)** and cisplatin **(B)** for 48 hours. Viability of Fibroblasts after exposure to different concentrations of doxorubicin **(C)** and cisplatin **(D)**. 0.1% (v/v) DMSO was used as the vehicle control. Data are expressed as the mean ± SEM of three biological assays. Statistical significance relative to the DMSO control was determined using one-way ANOVA (*$p < 0.05$).

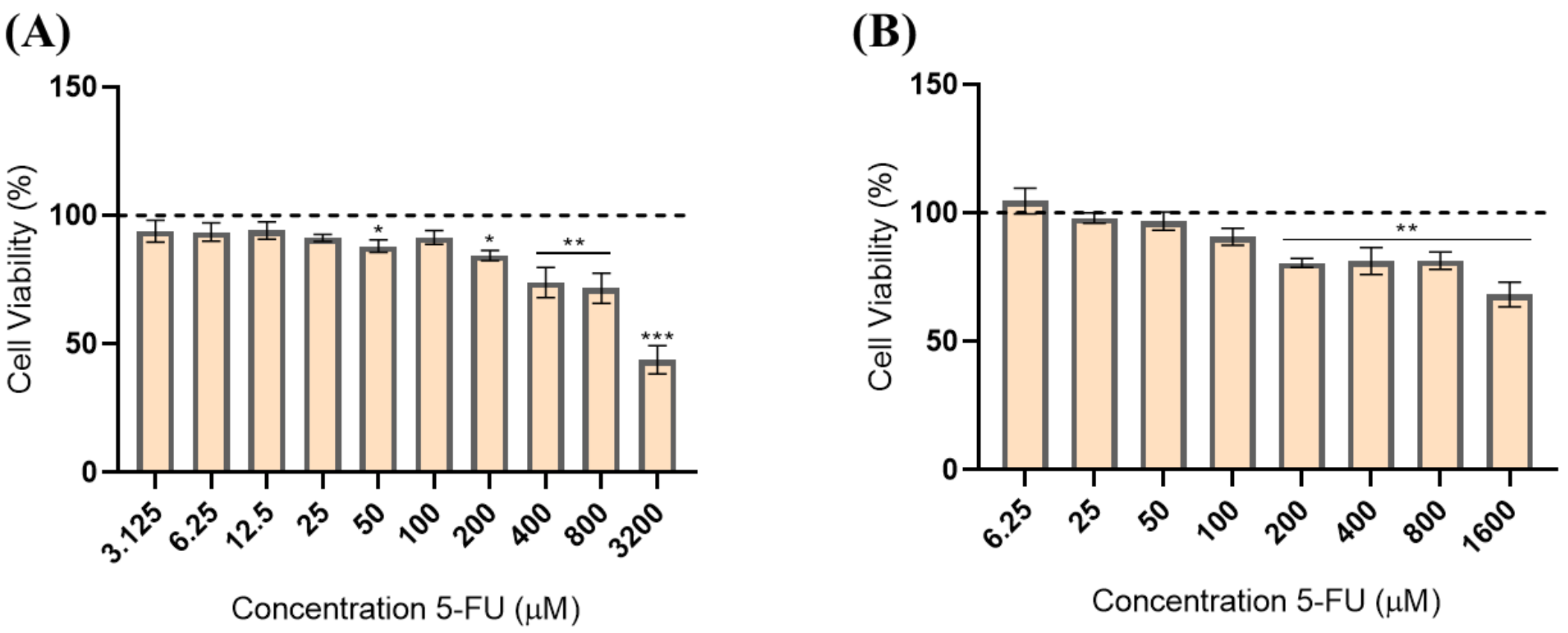

Figure S10. Viability of HCT116 **(A)** and HCT116-DoxR **(B)** 3D spheroids after exposure to different concentrations of FF regimen for 48 h. FF regimen was prepared with the ratios 1 5-FU: 0.042 FA: 0.012 OX, and the concentrations expressed in the graphic are based on the 5-FU concentration. The dashed line represents the DMSO control at the same final concentration used for the complexes. Data are presented as mean ± SEM from at least two independent biological replicates. Statistical significance relative to the DMSO control was determined using one-way ANOVA (**$p < 0.01$; ***$p < 0.001$; ****$p < 0.0001$).

Table S10. Clinical information of the patients included in the study and their respective PDOs; Tumor localtion; Tumor type; TNM (Tumor, nodule, and metastasis) scale for tumors staging; Biomarkers analysed - microsatellite instability by analysis of DNA repair genes *MLH1*, *PMS2*, *MSH2*, and *MSH6*, where MSS - Microsatellite Stable Tumors; MSI – microsatellite instable tumors; DPYD wt - dihydropyrimidine dehydrogenase wild-type – no mutation related with 5-FU metabolism. Neoadjuvant treatment (before surgery) – not applied in these patients; Adjuvant treatment for patient 3 – oral 5-FU. In the observations, mutated oncoproteins (BRAF, KRAS and/or NRAS) are depicted.

Patient ID	PDOs	Tumor location	Tumor type	TNM
**1**	1	Sigmoid	Mucinous adenocarcinoma	T4aN2bM1c
**2**	2	Transverse	Low-grade adenocarcinoma	T3N0M0
**3**	3	Cecum	Mucinous adenocarcinoma	T4bN2bM0
**4**	4 – Normal tissue-derived PDOs	Ascending	Low-grade adenocarcinoma	T2N0M0
**5**	4 – Tumor-derived PDOs	Ascending	Low-grade adenocarcinoma	T3N0M0

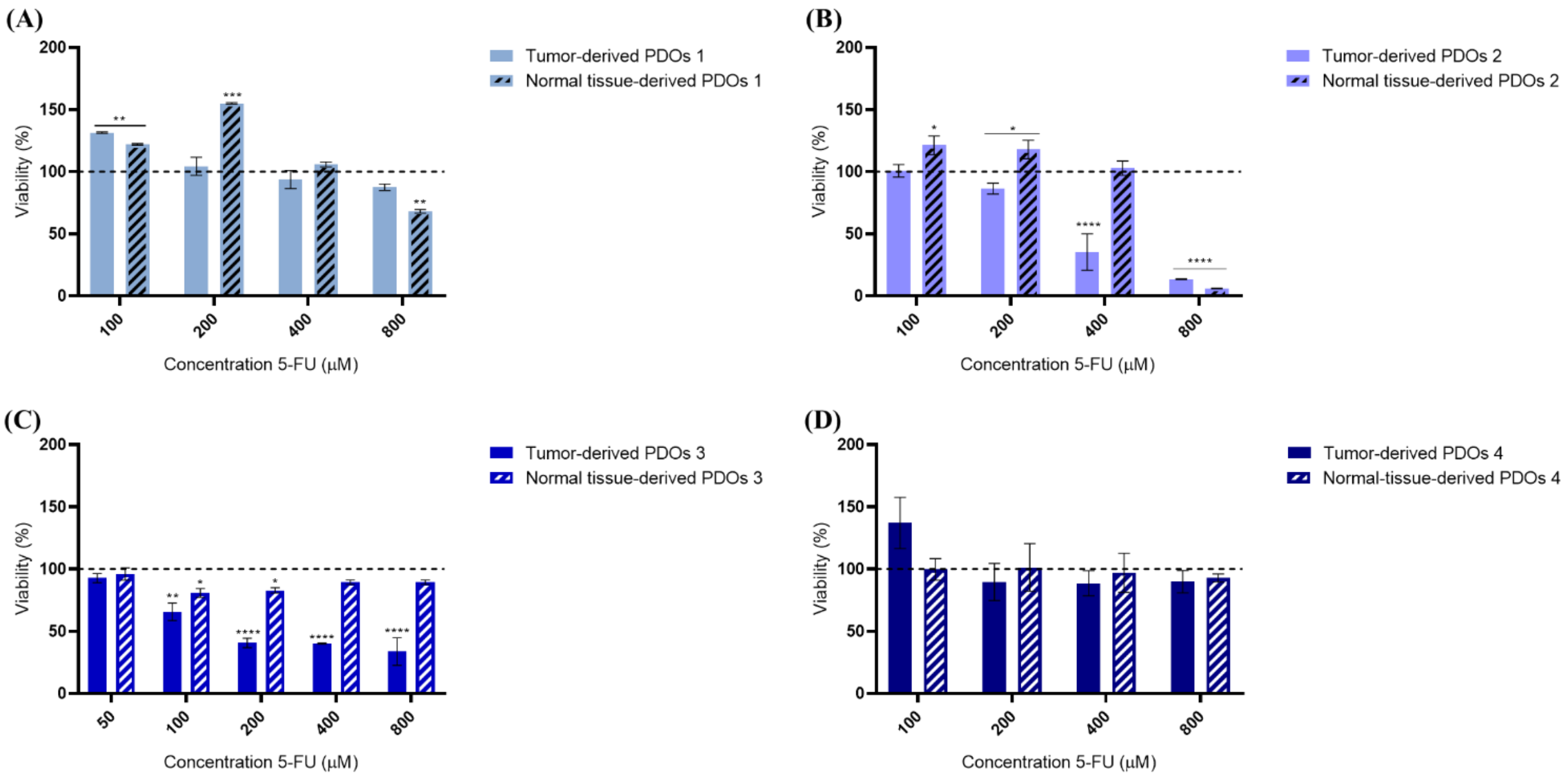

Figure S11. Viability of 4-day-old PDO pairs (1 – **A**; 2 – **B**; 3 – **C**; and 4 – **D**) after exposure to different concentrations of the FF regimen for 48 h. The FF regimen was prepared with the ratio 1 (5-FU):0.042 (FA):0.012 (OX), and the concentrations expressed in the graphic are based on the 5-FU concentration. The dashed line represents the vehicle control (DMSO + glucose (5-FU is dissolved in DMSO, and OX is dissolved in a 5% glucose solution)), at the same final concentrations used. Data are presented as mean ± SEM from at least two independent biological replicates. Statistical significance relative to the DMSO control was determined using one-way ANOVA (**$p < 0.01$; ***$p < 0.001$; ****$p < 0.0001$).

Table S11. Percentage of viable, early apoptotic, late apoptotic, and necrotic HCT116-DoxR cells after 48 h exposure to complex **1** (IC_{50} in 2D models), FF (IC_{10} in 2D models), and their respective combinations. DMSO and the corresponding FF vehicle controls were used as negative controls, while Cis served as positive control.

Sample	Cells (%)			
	Viable cells	Early apoptosis	Late apoptosis	Necrosis
**DMSO vehicle**	50.12 ± 2.51	48.19 ± 2.39	0.68 ± 0.11	0.86 ± 0.05
**FF + DMSO vehicles**	47.41 ± 2.26	50.95 ± 2.23	0.61 ± 0.05	1.00 ± 0.09
**Cis**	35.73 ± 2.76	58.69 ± 1.29	1.24 ± 0.17	1.72 ± 0.12
**FF IC_{10}**	31.62 ± 1.86	66.56 ± 2.32	0.55 ± 0.01	1.33 ± 0.42
**Complex 1**	23.74 ± 0.12	74.07 ± 0.65	0.89 ± 0.36	1.31 ± 0.40
**Complex 1 + FF**	11.17 ± 0.31	87.97 ± 0.30	0.53 ± 0.02	0.34 ± 0.02

Supplementary References

(1) Choroba, K.; Machura, B.; Penkala, M.; Erfurt, K.; Cordeiro, S.; Araújo, V.; Leopoldo, E.; Lima, R.; Afonso, D.; Baptista, P. V.; Fernandes, A. R. Therapeutic Potential of 2,2′:6′,2″-Terpyridine-Based Au(III), Pt(II), and Mn(II) Complexes against 2D and 3D Human Colorectal Resistant Cancer Cells. *J. Med. Chem.* **2025**, *68* (21), 23220–23246. https://doi.org/10.1021/acs.jmedchem.5c02066.

(2) Sheldrick, G. M. A Short History of SHELX. *Acta Cryst A* **2008**, *64* (1), 112–122. https://doi.org/10.1107/S0108767307043930.

(3) Sheldrick, G. M. Crystal Structure Refinement with SHELXL. *Acta Cryst C* **2015**, *71* (1), 3–8. https://doi.org/10.1107/S2053229614024218.

(4) Sheldrick, G. M. SHELXT – Integrated Space-Group and Crystal-Structure Determination. *Acta Cryst A* **2015**, *71* (1), 3–8. https://doi.org/10.1107/S2053273314026370.

(5) Llunell, M.; Casanova, D.; Cirera, J.; Alemany, P.; Gómez-Ruiz, S. SHAPE Program.

(6) Casanova, D.; Cirera, J.; Llunell, M.; Alemany, P.; Avnir, D.; Alvarez, S. Minimal Distortion Pathways in Polyhedral Rearrangements. *J. Am. Chem. Soc.* **2004**, *126* (6), 1755–1763. https://doi.org/10.1021/ja036479n.

(7) W. Addison, A.; Nageswara Rao, T.; Reedijk, J.; Rijn, J. van; C. Verschoor, G. Synthesis, Structure, and Spectroscopic Properties of Copper(II) Compounds Containing Nitrogen–Sulphur Donor Ligands; the Crystal and Molecular Structure of Aqua[1,7-Bis(N -Methylbenzimidazol-2′-Yl)-2,6-Dithiaheptane]Copper(II) Perchlorate. *Journal of the Chemical Society, Dalton Transactions* **1984**, *0* (7), 1349–1356. https://doi.org/10.1039/DT9840001349.
